# Ecdysteroid Derivatives that Reverse P-Glycoprotein-Mediated Drug Resistance

**DOI:** 10.1021/acs.jnatprod.0c00334

**Published:** 2020-08-13

**Authors:** Roberta Bortolozzi, Andrea Luraghi, Elena Mattiuzzo, Alessandro Sacchetti, Alessandra Silvani, Giampietro Viola

**Affiliations:** † Dipartimento di Salute della Donna e del Bambino, Laboratorio di Oncoematologia, Università degli Studi di Padova, Via Giustiniani 2, Padova, 35128, Italy; ‡ Istituto di Ricerca Pediatrica (IRP) Corso Stati Uniti 4, Padova, 35129, Italy; § Dipartimento di Chimica, Università di Milano Via Golgi 19, Milano, 20133, Italy; ∥ Dipartimento di Chimica, Materiali ed Ingegneria Chimica “Giulio Natta”, Politecnico di Milano, Piazza Leonardo da Vinci 32, Milano, 20133, Italy

## Abstract

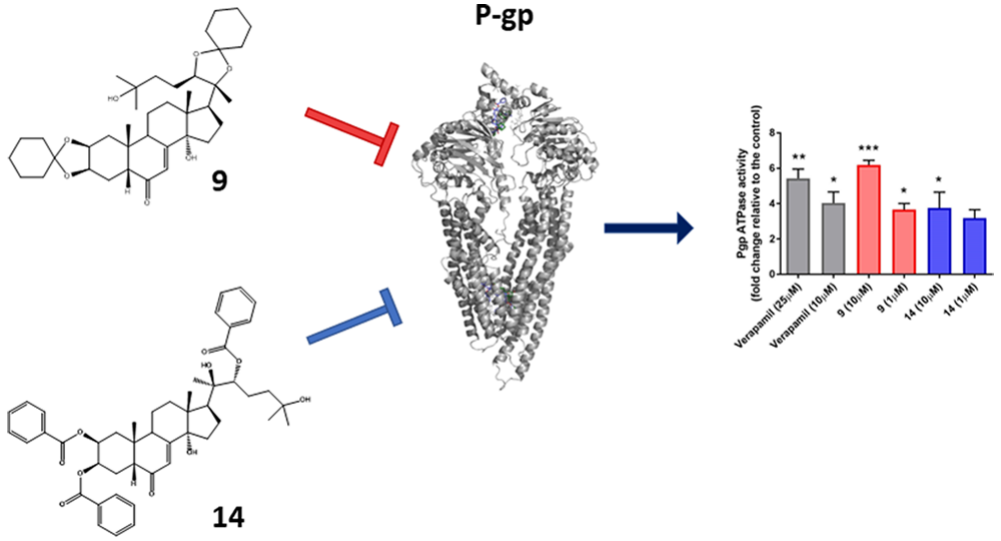

The expression of multidrug resistance P-glycoprotein (P-gp) by cancer cells represents one of the major drawbacks to successful cancer therapy. Accordingly, the development of drugs that inhibit the activity of this transporter remains a major challenge in cancer drug discovery. In this context, several new ecdysteroid derivatives have been synthesized and evaluated as P-gp inhibitors. Two of them (compounds **9** and **14**) were able to resensitize CEM^Vbl100^ and LoVo^Doxo^ resistant cell lines to vinblastine and doxorubicin, respectively. Indeed, both compounds **9** and **14** increased the cellular accumulation of rhodamine 123 in cells expressing P-gp and stimulated basal P-glycoprotein-ATPase activity at a 1 μM concentration, demonstrating their interference with the transport of other substrates in a competitive mode. Moreover, in a medulloblastoma cell line (DAOY), compounds **9** and **14** reduced the side population representing cancer stem cells, which are characterized by a high expression of ABC drug transporters. Further, in DAOY cells, the same two compounds synergized with cisplatin and vincristine, two drugs used commonly in the therapy of medulloblastoma. Molecular docking studies on the homology-modeled structure of the human P-glycoprotein provided a rationale for the biological results, validating the binding mode within the receptor site, in accordance with lipophilicity data and observed structure–activity relationship information. Altogether, the present results endorse these derivatives as promising P-gp inhibitors, and they may serve as candidates to reverse drug resistance in cancer cells.

Despite successful advances in cancer therapeutic strategies, multidrug resistance represents one of the primary causes of therapy failure.^1^ Biological membranes present a significant permeation barrier and thus play a critical role in the protection of pharmacokinetic compartments. Conversely, the activity of a drug ultimately depends on the ability of the compound to reach its target, as regulated by the basic physical characteristics of the drugs, as well as by their interactions with membrane transporters. A common mechanism shared by the majority of cancers is the overexpression of ATP-binding cassette (ABC) efflux transporters, including P-glycoprotein (P-gp), multidrug resistance proteins (MRPs), and breast cancer resistance protein (BCRP).^2^ ABC transporters are active components of biological membranes, but they act as a shield for drug-resistant cancer cells. Functional ABC transporters are large integral membrane proteins containing two transmembrane domains (TMDs) and two nucleotide-binding domains (NBDs).^2,3^ The molecular mechanism of transport is fueled by the energy of ATP hydrolysis, which results in a series of conformational changes, sweeping through the molecule from the cytoplasmic ATP-binding units to the TMD helices forming the transmembrane pore. ATP binding and hydrolysis regulate the association and disassociation of the NBD dimers, which is, in turn, coupled to a change in substrate binding affinity and transport.^4^ These membranous efflux pumps are able to extrude chemotherapeutics from cancer cells, preventing their uptake and the access to their cellular target. They promote the extrusion of structurally and functionally different chemotherapeutics, such as alkaloids, taxanes, topoisomerase inhibitors, and antimetabolites. The activity of ABC transporters has been associated with a poor prognosis, treatment failure, and reduced survival rate in many types of cancer, such as hematological malignancies, medulloblastoma, breast cancer, and pancreatic and colon carcinoma.^5^

Over the last few decades, one of the major challenges in cancer drug discovery has been the development of substances able to modulate/inhibit ABC efflux transporters. However, no compounds have been approved for cancer therapy, due to either their recurrent high intrinsic toxicity, pharmacokinetics interactions with anticancer drugs, with consequent increased toxicity of the anticancer drugs, or failure in demonstrating improvement in therapeutic efficacy.^6^

Medulloblastoma, the most frequent childhood primary malignant brain tumor, is generally treated with a combination therapy that includes etoposide, methotrexate, cisplatin, lomustine, cyclophosphamide, and vincristine.^7^ Recently, it has been demonstrated that the high frequency of recurrence and therapy failure in medulloblastoma is associated with a high expression of P-gp. This is crucial in children under three years that are treated with chemotherapy alone, to minimize the adverse effect of radiotherapy on the developing brain.^8^ In particular, ABCB1 is overexpressed in more than 40% of patient samples and is associated significantly with high risk and poor survival. Moreover, ABC transporters are highly expressed by the specialized endothelial cells that form the blood–brain barrier, and this constitutes a further obstacle to therapy success in brain tumors. In this context, strategies aimed at overcoming/inhibiting P-gp, thus enhancing the efficacy of chemotherapy, are still warranted.

Ecdysteroids represent a large family of steroid hormones, playing a crucial role in arthropod physiology.^9^ The most abundant representative of these compounds, 20-hydroxyecdysone, regulates the reproduction, embryogenesis, diapause, and molting of arthropods. Their role in plants is still to be fully understood, but it has been suggested that they have importance in several plants as defensive agents against nonadapted herbivores. An estimated 5–6% of terrestrial plant species accumulate detectable levels of ecdysteroids.^10^ Their common chemical skeleton retains the cholesterol-originated side chain, contains typically 27–29 carbon atoms, and is substituted with 4–8 hydroxy groups. The A/B ring junction is usually *cis*, and a characteristic 7-en-6-one (α,β-unsaturated ketone) functional group is present in ring B.

Due to their significantly different structure as compared to vertebrate steroid hormones, ecdysteroids have no hormonal effects in humans.^11^ Rather, it has been shown that ecdysteroids are nontoxic in mammals.^12^ An oral LD_50_ value of higher than 6 g/kg in mice and a wide range of beneficial health effects (including adaptogenic, anabolic, antihyperglycemic, hepatoprotective, immunoprotective, and wound healing) were described,^9^ which has encouraged the production and worldwide marketing of many food supplements, mainly containing 20-hydroxyecdysone.

Moving to medicinal chemistry studies, it has been demonstrated by Martins and co-workers^13^ that semisynthetic derivatives of ecdysteroids are able to inhibit the ABCB1 transporter and to revert resistance to doxorubicin in mammalian cancer cells expressing the human ABCB1 transporter.^13,14^ Their studies have identified lipophilicity as the key feature for the in vitro activity of the compounds, showing how the inactive natural compound 20-hydroxyecdysone may become a promising lead compound, after transformation to the corresponding diacetonide derivative.

Recently, an efficient multicomponent synthetic protocol has been developed by our group, starting from the easily available 20-hydroxyecdysone and allowing access to different kinds of peptide–ecdysteroid conjugates, which were evaluated for their ability to inhibit the ABCB1 pump.^15^ Progressing in this interest in ecdysteroids as potential multidrug resistance modulators,^16^ we aimed to investigate more deeply the role of the steroidal skeleton on bioactivity, also evaluating rarer and more expensive ecdysteroids. For this purpose, natural compounds **1**–**5** were selected by considering their structural diversification in terms of the number and position of the hydroxy groups present. In fact, this distinctive feature allows the design of a more varied library of functional derivatives, both esters and ketals, in order to go deeper into the role of lipophilicity and steric encumbrance, related to biological activity.

In the present study, the synthesis and structural characterization of new ecdysteroids derivatives are reported, as well as the evaluation of their ability to modulate P-gp-mediated drug efflux in multidrug resistant (MDR) cell lines. For the most active compounds, their ability to modulate Pgp-ATPase activity was also investigated, in an effort to assess their potential as new drug candidates to treat multi-drug-resistant cancers.

## Results and Discussion

### Synthesis of Ecdysteroid Derivatives (**6**–**28**)

**Figure 1:**
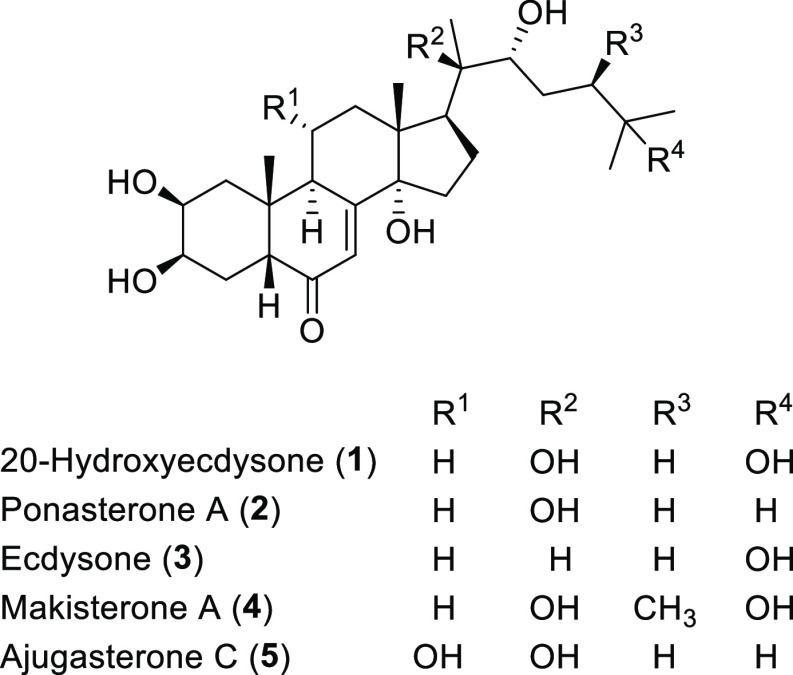
Natural ecdysteroids used as starting materials.

Starting from ecdysteroids **1**–**5** (Figure 1) and exploiting different chemical modifications of their hydroxy functional groups, two small families of derivatives, namely, 2,3–20,22 bis-ketals and various polyesters, were synthesized.

**Scheme 1:**
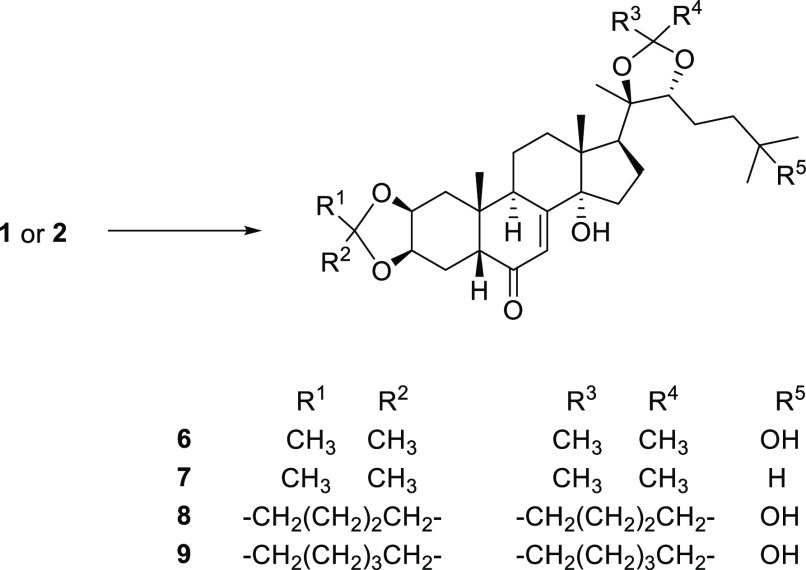
Synthesis of Ecdysteroid 2,3–20,22 Bis-Ketals (**6**–**9**) Reagents. General procedure A: camphosulfonic acid (0.01 mmol), **1** or **2** (0.1 mmol), appropriate anhydrous ketone (1.6 mL), 25 °C, 24–72 h.

The 2,3–20,22 bis-ketals **6**–**9** were synthesized by adapting typical ketalization procedures (Scheme 1). Briefly, the reaction was carried out employing the appropriate ketone (acetone, cyclopentanone, or cyclohexanone) as solvent and camphosulfonic acid as catalyst and afforded the products in variable yields (67–97%).

**Scheme 2:**
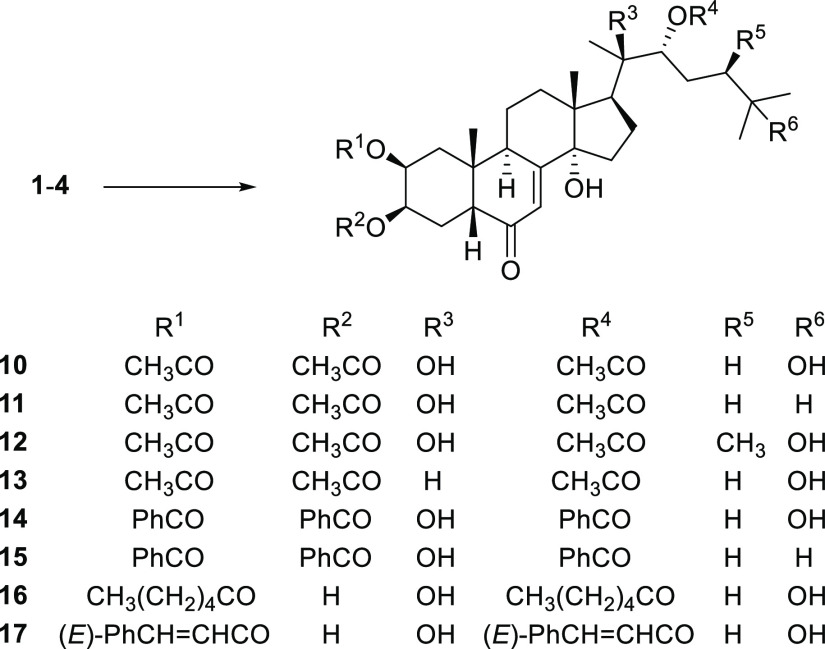
Synthesis of Ecdysteroid Esters (**10**–**17**) Reagents. General Procedure B: **1**–**4** (0.1 mmol), appropriate carboxylic acid anhydride or chloride (0.5 mmol), pyridine (2.5 mL), 0 °C, 8–24 h.

For the preparation of ester derivatives, two different protocols were considered. In the case of nonbulky esters, such as acetates and benzoates, standard conditions were applied. Employing the appropriate carboxylic acid anhydride or chloride in pyridine as solvent, the 2,3,22-triesters **10**–**15** were achieved in good yields. In such conditions, bulkier acylating agents (hexanoyl chloride and cinnamoyl chloride) gave exclusively the 2,22-diesters **16** and **17** in comparable yields (Scheme 2).

**Scheme 3:**
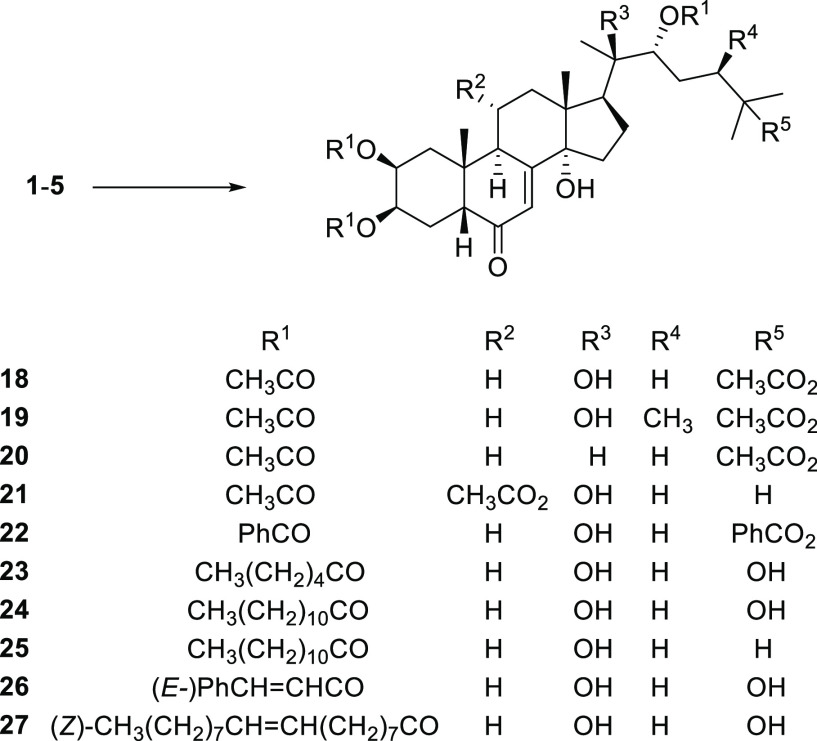
Synthesis of Ecdysteroid Esters (**18**–**27**) Reagents. General Procedure C: **1**–**5** (0.1 mmol), *N,N*′-dimethylaminopyridine (0.4 mmol), triethylamine (0.4 mmol), appropriate carboxylic acid chloride (0.44 mmol), CH_2_Cl_2_ (2.5 mL), 0 °C, 1–4 days.

Aiming to investigate compounds at different degrees of lipophilicity, also the synthesis of tetra-ester derivatives was pursued. Preparation of tetraacetates **18**–**21** and tetrabenzoate **22** required modified reaction conditions, namely, the catalysis of *N,N′*-dimethylaminopyridine/triethylamine in dichloromethane and longer reaction times (up to 96 h) (Scheme 3). Under these conditions, 2,3,22-triester derivatives incorporating bulky acidic residues were obtained, such as 20-hydroxyecdysone trihexanoate (**23**) and trilaurate (**24**), ponasterone A trilaurate (**25**), and 20-hydroxyecdysone tricinnamate (**26**) and trioleate (**27**), all in acceptable yields.

**Scheme 4:**
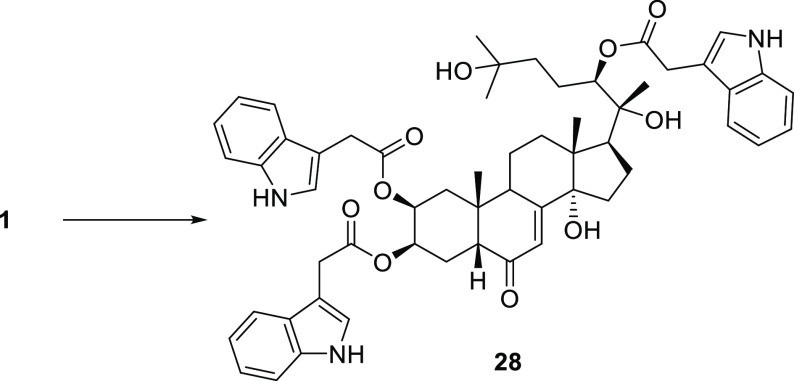
Synthesis of 20-Hydroxyecdysone-2,3,22-tri(2-(1*H*-indol-3-yl) Acetate (**28**) Reagents. 2-(1*H*-indol-3-yl)acetic acid (0.7 mmol), DCC (0.7 mmol), dry dioxane (3 mL), 1 h; then urea was filtered off and the filtrate added to **1** (0.1 mmol), *N,N*′-dimethylaminopyridine (0.05 mmol), dry dioxane (3 mL), 40 °C, 22 h.

Finally, reacting 20-hydroxyecdysone (**1**) with indolyl-3-acetic acid anhydride (generated in situ by treatment of indolyl-3-acetic acid with dicyclohexylcarbodiimide (DCC) in anhydrous dioxane), the 2,3,22-tris(2-(1*H*-indol-3-yl) acetate derivative **28** was obtained in 83% yield (Scheme 4).

### Compounds **9** and **14** Induce Significant Accumulation of Rhodamine 123 in CEM^Vbl100^ and Lovo^Doxo^ ABCB1 Overexpressing Cell Lines

To assess the capability of the novel synthesized ecdysteroids to modulate the ABCB1 multidrug resistance pump, the CEM^Vbl100^ and LoVo^Doxo^ cell lines were exposed to rhodamine 123 (Rho123), a fluorescent compound known as a good substrate of P-gp.^17^ The mean fluorescence intensity (MFI) was evaluated by flow cytometry. Compounds **24**, **25**, and **27** were not evaluated due to their very low solubility in aqueous media.

**Figure 2:**
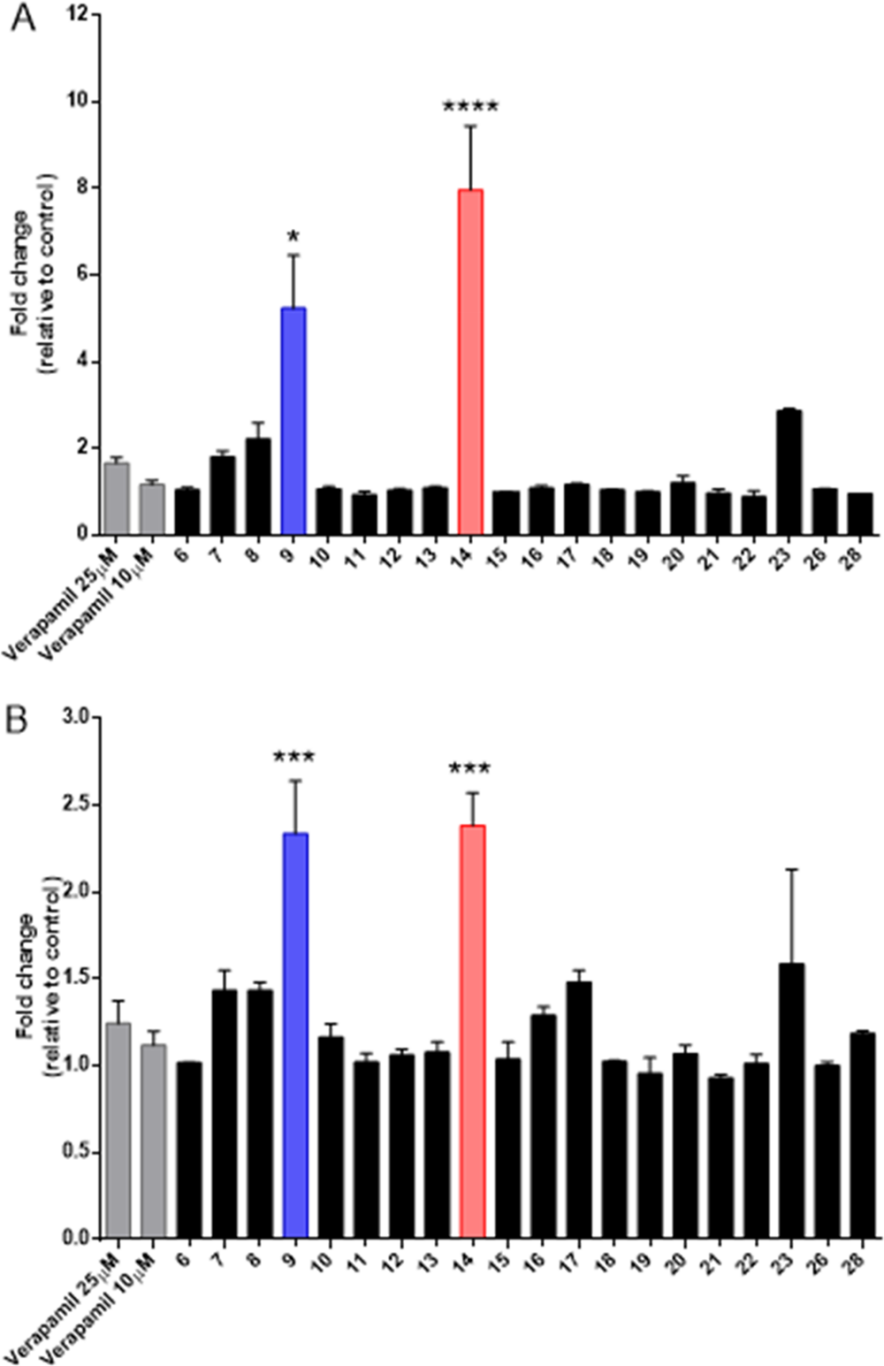
Quantification presented by fold change in Rho123 fluorescence after a 2 h treatment of CEM^Vbl100^ cells (panel A) and LoVo^Doxo^ cells (panel B), compared to untreated cells. Each compound was used at a concentration of 10 μM. Verapamil was used at concentrations of 25 and 10 μM. Data are represented as means ± SEM of three independent experiments. Statistical significance was determined using ANOVA with Newman–Keuls or Bonferroni correction. Asterisks indicate a significant difference between the new compounds and verapamil at 25 μM. *p < 0.05, **p < 0.01, ***p < 0.001, ****p < 0.0001.

Cells were treated for 2 h with compounds at the concentration of 10 μM and with verapamil at 25 and 10 μM, as a positive control. As depicted in Figure 2, compounds **9** and **14**, in CEM^Vbl100^ cells, prevented the efflux of Rho123 more than verapamil (fold change of MFI relative to the control of 5.2 ± 1.2 and 8.0 ± 1.5 vs 1.6 ± 0.12). Similar results, although less pronounced, were obtained in LoVo^Doxo^ cells.

The data obtained indicated compounds **9** and **14** as the most promising ones and suggest that a possible structure–activity relationship (SAR) can be proposed considering the lipophilic profile of these examined compounds. Both active compounds **9** and **14** are derivatives of 20-OH ecdysone **1**. Among bis-ketals, compound **9** is the most lipophilic, due to the presence of two spiro-cyclohexane rings. On only slightly reducing the spiro-ring size, such as in the corresponding spiro-cyclopentane derivative **8**, the activity dramatically decreased, as well as in the acetone ketal derivatives **6** and **7**. Moving to ester derivatives, the tribenzoic ester **14** displayed the highest activity among all compounds tested.

Since it was known^13^ that less polar ecdysteroids are endowed with a better activity profile in comparison to those of higher polarity, the cLogP was calculated for all new derivatives (Table 1). The two most active compounds, **9** and **14**, have cLogP values of 5.75 and 6.65, respectively, representing the optimal range for activity. As was observed, all other compounds have higher or lower cLogP values, with the only exceptions being derivatives **7** (cLogP 6.06) and **28** (cLogP 6.27). However, for these two compounds, some considerations on their specific structural features can be made. Compound **7** is characterized by a penalizing low lipophilicity in the region of ring A, due to the presence of the acetone ketal. If compared with its analogue **6** (cLogP 3.85), the increased cLogP of **7** is only due to the lack of the OH-25 group. However, from previous SAR studies, the role of this portion of the molecule seems not to be crucial for receptor interaction and activity.^14^ In the case of compound **28**, the optimal value of cLogP is likely counteracted by the presence of the bulky indolyl-3-acetic residues, which hinders the correct interaction with the small M-site located on the transmembrane domain of the P-gp (see below for molecular modeling discussion).^18^

**Table 1:** Lipophilic Profile of Ecdysteroid Derivatives **6**–**28**

compound	cLogP	compound	cLogP	compound	cLogP
**6**	3.85	**13**	2.85	**20**	3.79
**7**	6.06	**14**	6.65	**21**	2.50
**8**	4.12	**15**	8.85	**22**	9.51
**9**	5.75	**16**	5.31	**23**	7.79
**10**	1.44	**17**	4.86	**26**	7.90
**11**	3.65	**18**	2.39	**28**	6.27
**12**	1.84	**19**	2.78		

When cLogP becomes too high, other phenomena related to poor pharmacodynamics can lead to a low activity. This is the case of inactive compound **15**, which is quite similar to active **14**, but shows a cLogP of 8.85, due to the lack of the OH-25 group.

Given the great difference in activity of compounds **9** and **14** compared to all the other test compounds, a subsequent more in-depth assessment of the biological profile, including the P-gp inhibitory behavior, was carried out only on these two derivatives.

### Effect of Compounds **9** and **14** on ABCB1-Mediated Resistance to Vinblastine and Doxorubicin in ABCB1-Overexpressing Drug-Selected Cell Lines

**Figure 3:**
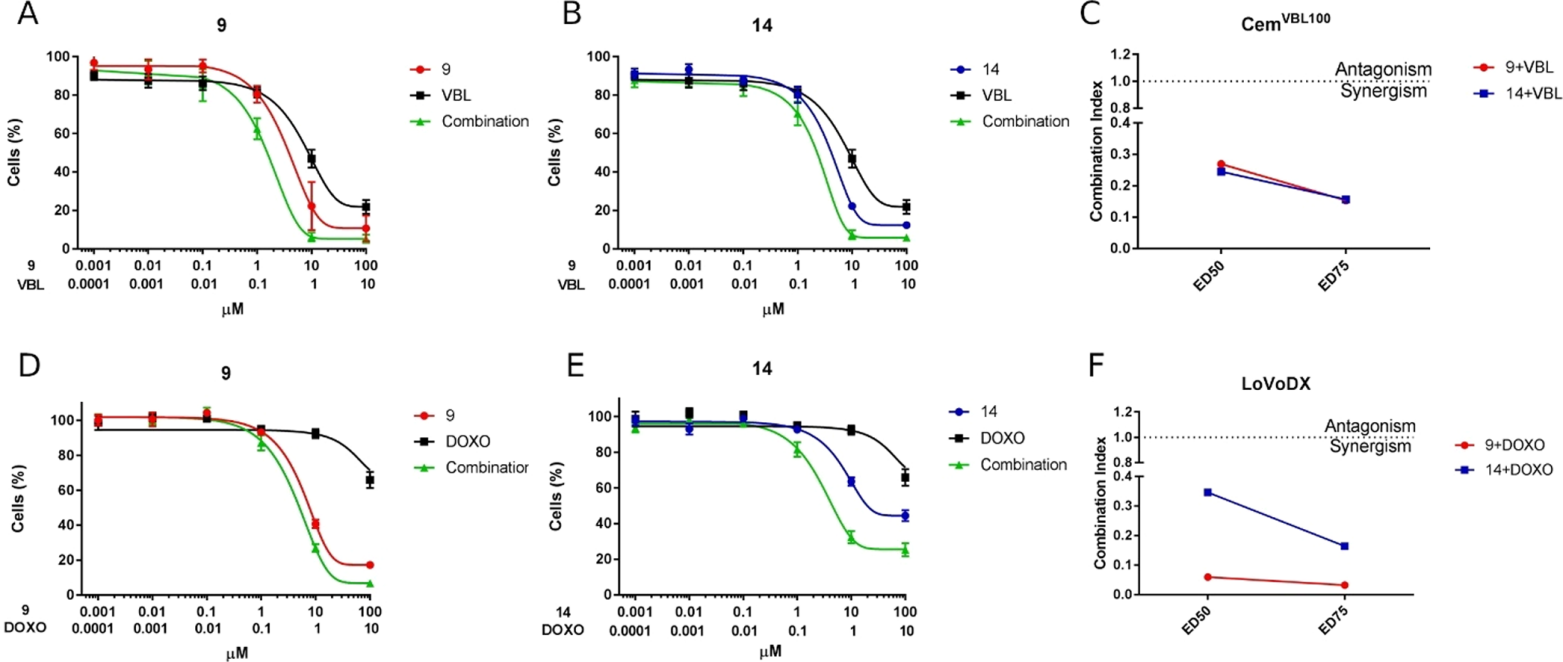
Effect of compounds **9** and **14** alone and in combination with vinblastine in CEM^Vbl100^ (panels A and B) and doxorubicin in LoVo^Doxo^ (panels D and E) cells. Cells were treated at the indicated concentrations and fixed combination ratios, and viability was assessed by the MTT test after 48 h of incubation. Data are expressed as means ± SEM of three independent experiments. Combination indexes (CI) are calculated at the ED_50_ and ED_75_ for vinblastine (panel C) and doxorubicin (panel F) combination, where synergism is defined by CI < 1.

Compounds **9** and **14** were investigated further for their ability to enhance the activity of vinblastine in the CEM^Vbl100^ cell line and of doxorubicin in the LoVo^Doxo^ cell line. Cells were treated with vinblastine in combination with **9** and **14** at a fixed concentration ratio (1:10), and cell viability was analyzed by an MTT assay after 48 h. As depicted in Figure 3, both compounds induced a significant increase in the cytotoxicity of vinblastine in a synergistic way, as demonstrated by combination indexes (CI) < 1, as calculated according to Chou et al.^19,20^

Moreover, compounds **9** and **14** were also able to increase the cytotoxicity of doxorubicin in ABCB1-overexpressing LoVo^Doxo^ cells. Also in this case, combination indexes calculated after 48 h of treatment indicated a synergistic effect between doxorubicin and compound **9** or **14**.

### Effects of Compounds **9** and **14** in Human Peripheral Blood Lymphocytes

Aiming to obtain a preliminary indication of their cytotoxic potential in normal human cells, new ecdysteroids derivatives **9** and **14** were evaluated in vitro against peripheral blood lymphocytes (PBLs) from healthy donors. As depicted in Table 2, compound **9** showed a low toxicity both in quiescent and in proliferating lymphocytes in the presence of the mitogenic stimulus phytohematoaglutinin (PHA), having a GI_50_ of 61.7 and 42.8 μM, respectively. On the other hand, compound **14** was practically devoid of activity in quiescent lymphocytes, while it showed a weak cytotoxic activity in proliferating lymphocytes.

**Table 2:** Cytotoxicity of **9** and **14** in Human Peripheral Blood Lymphocytes (PBLs)

	GI_50_ (μM)^a^	
compound	PBLs_resting_^b^	PBLs_PHA_^c^
**9**	61.7 ± 9.6	42.8 ± 16.1
**14**	>100	19.9 ± 6.7

a Compound concentration required to inhibit cell growth by 50%.

b PBLs not stimulated with PHA.

c PBLs stimulated with PHA.

### Effect of Compounds **9** and **14** on the Basal ATPase Activity of P-gp

**Figure 4:**
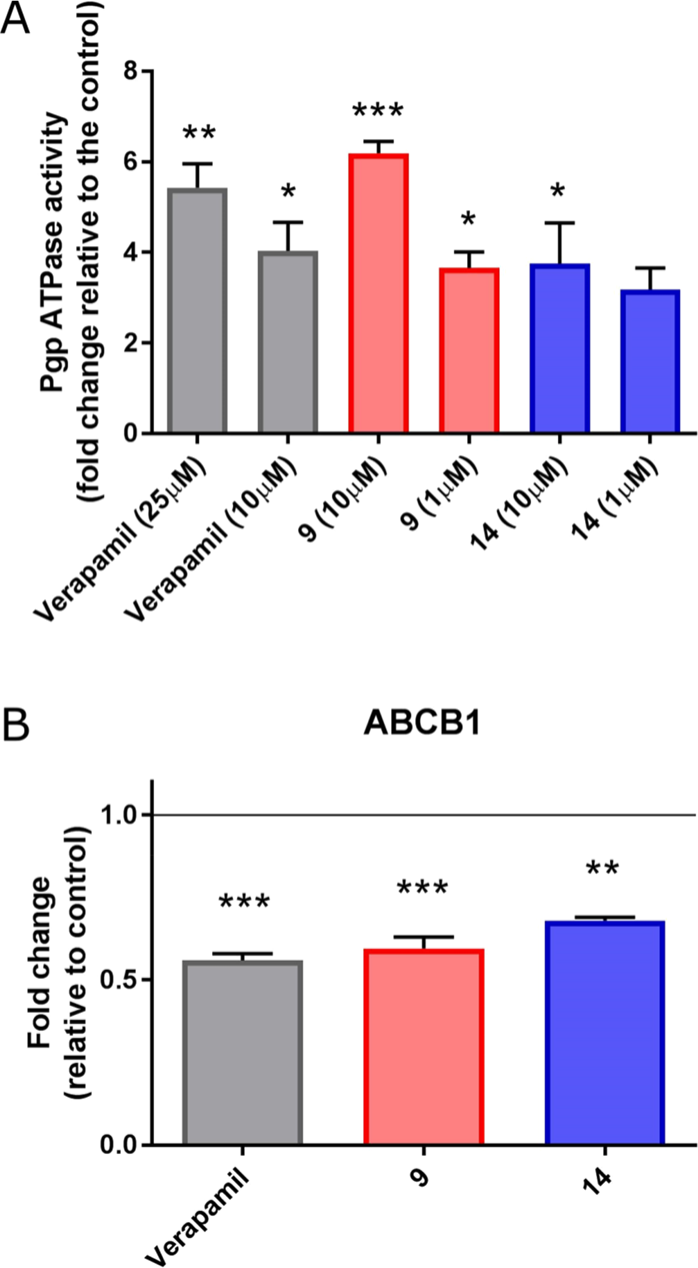
(A) Effects of compounds **9** and **14** on the ATPase activity of human P-gp. Each compound was tested at the concentrations of 1 and 10 μM, and verapamil at concentrations of 10 and 25 μM. The P-gp ATPase activity was expressed as fold changes, compared to untreated controls. (B) RT-PCR analysis of P-gp expression level on the CEM^Vbl100^ cell line, after exposure of compounds **9** and **14** at a concentration of 10 μM for 24 h. Verapamil was used at a concentration of 25 μM. Data are expressed as means ± SEM of three independent experiments. *p < 0.05; **p < 0.01; ***p < 0.001.

To clarify further the mode of action of compounds **9** and **14**, their effect on basal P-glycoprotein-ATPase activity was evaluated on recombinant human P-gp in a cell membrane fraction. Verapamil, a competitive inhibitor of P-gp activity that stimulates ATPase activity, was used as a positive control. At concentrations of 1 and 10 μM, compounds **9** and **14** stimulated basal P-glycoprotein-ATPase activity by 6-fold and by 4-fold, respectively, whereas verapamil, at 25 and 10 μM concentrations, stimulated the ATPase activity by about 6- and 4-fold, respectively (Figure 4, panel A). These data demonstrate that compounds **9** and **14** are potent stimulators of ATPase activity of P-gp, and thus, in the same manner of verapamil, both compounds interfere with P-gp transport activity, in a competitive way with another substrate, as described above.

### Compounds **9** and **14** Decrease P-Glycoprotein Expression in the Multi-Drug-Resistant CEM^Vbl100^ Cell Line

Muller and co-workers^21^ demonstrated previously that another approach to reverse multidrug resistance can be the modulation of transcriptional regulation of ABCB1 by pharmacological agents. In particular, they determined that verapamil treatment induces a decrease in the mRNA expression level of the mdr1 gene, through a transcriptional or post-transcriptional mechanism. In this context, the effects of compounds **9** and **14** on the mdr1/P-gp expression level were measured on the CEM^Vbl100^ cell line, by real-time reverse transcriptase polymerase chain reaction (RT-PCR). As shown in Figure 4 (panel B), a significant decrease in mdr1 mRNA expression was observed after 24 h of treatment, with **9** or **14** at a concentration of 10 μM. Treatment with 25 μM verapamil was performed as a positive control.

### Compounds **9** and **14** Reduce the Side Population Subset and Sensitize the Medulloblastoma DAOY Cell Line to Vincristine and Cisplatin

A particular characteristic of stem cells is the high expression level of specific ABC drug transporters.^22^ Like normal stem cells, also a subpopulation of cancer stem cells, known as “side population” (SP) cells, highly express ABC transporters and may be analyzed by flow cytometry, owing to their ability to extrude Hoechst 33342 dye.^23^ SP cells are pluripotent and show resistance to many cytotoxic drugs, unlike non-SP cells.^24^ SP cells have been identified successfully in a wide range of solid tumors, including breast, lung, prostate, ovarian, glioma, and pancreatic cancers^25−30^ and have been described as playing a critical role in tumor initiation, maintenance, progression, and relapse.^31^

**Figure 5:**
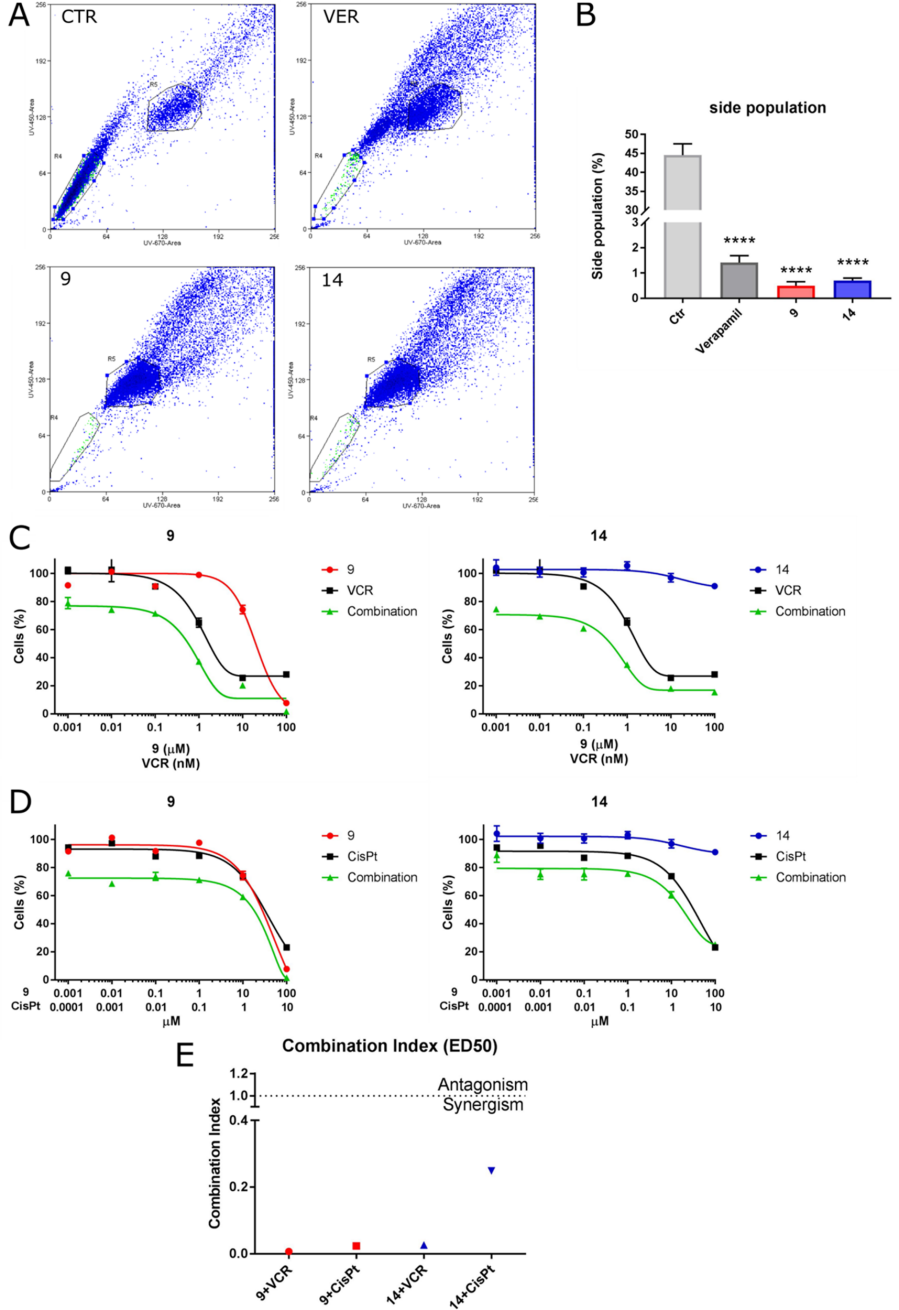
(A) Flow cytometric analysis of SP cells in a medulloblastoma cell line (DAOY). Representative histograms obtained after 2 h of treatment with compounds **9** and **14** at a concentration of 10 μM. Verapamil (25 μM) was used as a positive control. (B) Quantification of SP cells under the conditions described in panel A. Data are expressed as means ± SEM. ****p < 0.0001 vs ctr. (C) MTT cell viability assay in the DAOY cell line treated with compounds **9** and **14** in combination with vincristine (VCR) or cisplatin (cisPt) for 72 h. The percentages of cell viability were normalized to untreated cells. Data are represented as the means ± SEM of at least three independent experiments. (E) The combination index (CI) calculated at the ED_50_ for VCR with the cisplatin synergism is defined by CI < 1.

To evaluate the ability of **9** and **14** to target SP cells by inhibition of an ABCB1 transporter, medulloblastoma DAOY cells were treated with **9**, **14**, or verapamil for 2 h, and the capacity of cells to extrude Hoechst dye was measured by flow cytometry. Compounds **9** and **14** were able to inhibit the ABCB1 transporter reducing SP population, as shown in Figure 5 (panels A and B).

In order to assess if ecdysteroid derivatives **9** and **14** could enhance the efficacy of drugs commonly used in medulloblastoma therapy protocols, DAOY cells were treated with cisplatin or vincristine in the presence or absence of **14** or **9**, at a fixed molar combination. As depicted in Figure 5 (panels C–E), both compounds synergized with the chemotherapy used, as confirmed by the low CI value, as calculated by Chou’s method.^19,20^ Such results therefore show that the pharmacological inhibition of the P-gp activity, carried out by **9** and **14**, significantly increases the cell death effects induced by the treatment with the conventional chemotherapeutic agents.

### Molecular Modeling

In order to elucidate the possible interaction of the active ecdysteroid derivatives with the receptor, a docking study was performed, using a homology-modeled structure of the human P-glycoprotein. This receptor has been recognized to have two main binding sites, the cytosolic nucleotide-binding domain and the transmembrane domain, formed by six helices.^32,33^ In the latter, three different sites have been described according to the drug bonded, namely, the R-site, the M-site, and the H-site.^18^ The M-site site has the smallest volume if compared with the other two sites. Docking calculations were run for the two most promising molecules, **9** and **14**, and both the NMD and TMD binding sites were explored. The resulting conformations were clustered according to a heavy atom RMSD < 5 Å. Besides taking into account the lowest energy pose, for each cluster the energy spread (average and standard deviation) was also evaluated. The results are reported in Table 3. For both compounds, the lowest binding energies were obtained from the interaction with the TMD site. The lower number of clusters found for **9** could be ascribed to the lower flexibility of such a derivative, likely due to the presence of two spiro-cyclohexane rings. The lowest energy poses have similar energy values (−13.15 and −13.30 kcal/mol, for **9** and **14**, respectively). Cluster analysis apparently indicates a preference for **14** (1 member in the lowest energy cluster; −13.30 ± 0.00 kcal/mol) over **9** (32 members in the lowest energy cluster; −11.94 ± 0.81 kcal/mol). Thus, if the binding energy of the first most populated cluster is considered, **9** (−11.94 ± 0.81 kcal/mol) showed a more favorable interaction with the receptor than **14** (−9.10 ± 0.96 kcal/mol). Similar considerations were made for the NBD site, where again **9** displayed the lowest binding energy. In summary, from this analysis, it appears that compounds **9** and **14** preferentially bind at the TMD site of the P-glycoprotein with almost equal efficiency, in accordance with biological data.

**Table 3:** Results from Docking Studies on the Human P-Glycoprotein Model Receptor

	transmembrane domain (TMD)				nucleotide binding domain (NMD)			
	no. of clusters	lowest bind. *E*^a^	lowest bind. *E*^b^	binding energy of the first most populated cluster	no. of clusters	lowest bind. *E*^a^	lowest bind. *E*^b^	binding energy of the first most populated cluster
**9**	20	–13.15	–11.94 ± 0.81	–11.94 ± 0.81	18	–10.26	–9.13 ± 0.73	–9.02 ± 0.67
**14**	43	–13.30	–13.30 ± 0.00	–9.10 ± 0.96	54	–8.66	–7.69 ± 0.00	–6.33 ± 0.48

a Global minimum (kcal/mol).

b After clustering (kcal/mol).

Within the TMD binding site, the ligands are in contact with the side-chain residues as a result of hydrophobic and π–π interactions. A detailed analysis of the main interacting residues (Met 68, Met 67, Phe 336, Ile340, Tyr 953, Phe 978, Met 986) established that the ligands are located preferentially in the M-site of the receptor. These findings are in agreement with a previously reported study on the action of cardiotonic steroids as P-glycoprotein inhibitors.^34^

**Figure 6:**
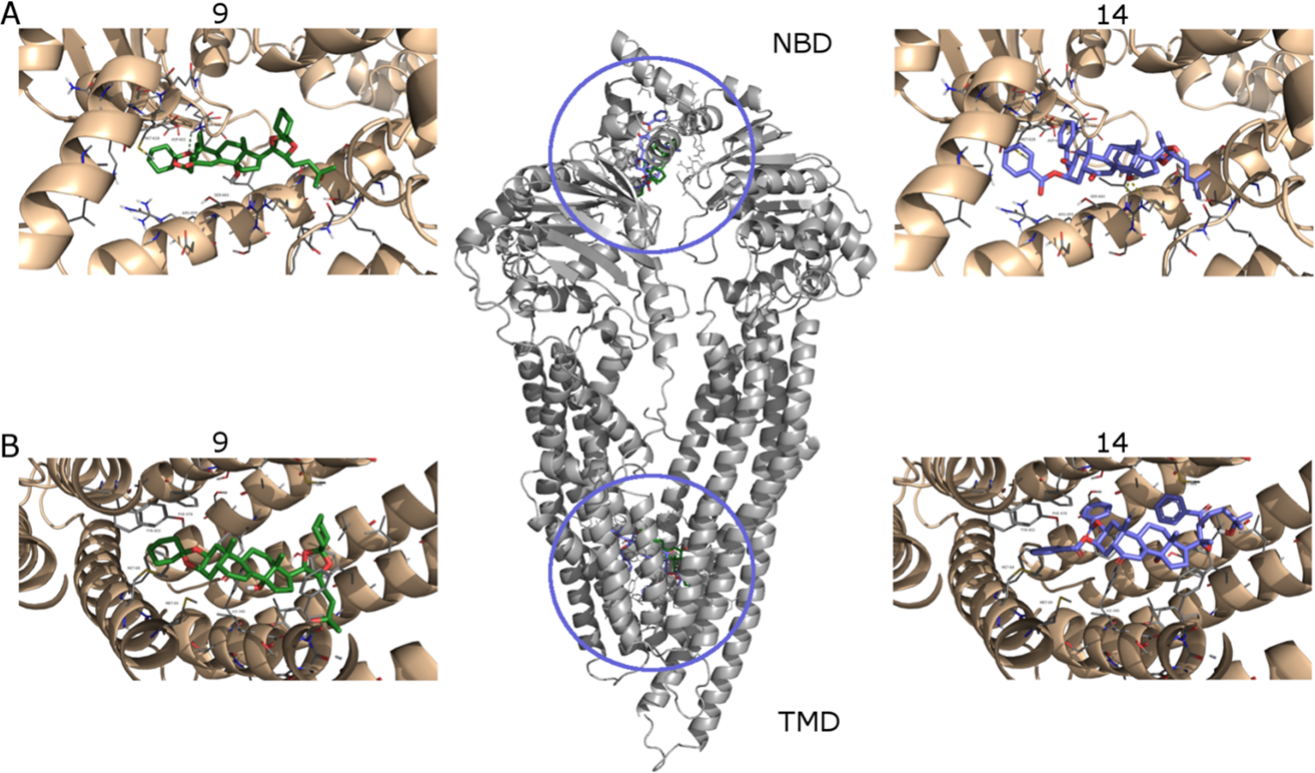
Binding modes for compound **9** (left, green color) and **14** (right, blue color) in the NMD (A, upper) and TMD (B, lower) domains, as obtained from docking simulations.

In summary, starting from natural ecdysteroids **1**–**5** and exploiting the reactivity of their hydroxy functional groups, a series of derivatives was synthesized with the aim of modulating their P-gp inhibitory activity. From the Rh123 assay, compounds **9** and **14** were identified, among the series, as the most promising compounds as potential P-gp inhibitors. Such derivatives are endowed with very low toxicity, as demonstrated by the high values of GI_50_ evaluated in human PBL. Concerning the capacity to reverse P-gp activity in CEM^Vbl100^ cells, compounds **9** and **14** at a concentration of 10 μM were approximately 4- and 7- fold, respectively, more potent than verapamil used at the same concentration. Less pronounced, but still significant, is the effect of **9** and **14** in LoVo^Doxo^ cells, another P-gp-overexpressing cell line, where both compounds ware observed as 1.5-fold more potent than verapamil.

From a mechanistic point of view, both compounds **9** and **14** induce P-gp ATPase activity, suggesting that they behave as substrates for transport, thereby inhibiting P-gp activity by competition with other substrates. Interestingly, derivatives **9** and **14** are able to reduce significantly the percentage of the side population in the medulloblastoma cell line DAOY. As it is well known, the side population represents that fraction of cells, also known as cancer stem cells or tumor-initiating cells, that are characterized by the presence of a high level of drug-efflux pumps. In this context, the fact that these two new compounds are capable of inducing a synergistic effect when combined with cisplatin or vincristine, two drugs used in the treatment of medulloblastoma, is particularly relevant.

In the present work, it was found also that the preferred binding site for the most active compounds within the P-gp is the M-site, in agreement with the literature for the binding of steroids on P-gp.^18^ Due to an increased presence of aromatic residues (Phe and Tyr), the M-site provides a more hydrophobic environment, compared to the H-site and the R-site. From docking experiments, it appears that the presence of highly lipophilic residues on the A ring is beneficial to activity, since this part of the molecule is buried in the most hydrophobic region of the M-site. Moreover, the presence of aromatic rings in this region of the ligand enhances the site interaction, through π–π contacts with aromatic residues.

In conclusion, with compounds **9** and **14**, the best possible arrangement of functional groups within the active binding site takes place, making these ecdysteroid derivatives the most promising candidates as P-gp inhibitors. Relying on a considerable amount of favorable biological data, they have the potential to be used in association with conventional chemotherapies in the treatment of cancers affected by P-gp-mediated MDR.

## Experimental Section

### General Experimental Procedures

All solvents and reagents were purchased from commercial sources and used without further purification. All reactions were carried out under a dry nitrogen atmosphere and were monitored by thin-layer chromatography (TLC) on precoated silica gel 60 F254; spots were visualized with UV light or by treatment with a 1% aqueous KMnO_4_ solution. Optical rotations were determined on a JASCO P-1030 polarimeter using the sodium D line (λ = 589 nm) at 20 °C in MeOH. ^1^H and ^13^C NMR spectra were recorded in CDCl_3_ on a Bruker Advance 400 spectrometer (400 and 101 MHz) with TMS as internal standard. Chemical shifts are reported in parts per million relative to the residual solvent. Multiplicities in ^1^H NMR are reported as follows: s = singlet, d = doublet, t = triplet, m = multiplet, br = broad. HRMS were measured in ESI mode on a Waters Q-Tof Micromass instrument equipped with a TOF mass analyzer. Products were purified by flash chromatography (FC) on Merck silica gel 60 (230–400 mesh).

### General Procedure A for Ketalization of Ecdysteroids (GP-A)

Camphosulfonic acid (2.3 mg, 0.01 mmol) was added to a stirred suspension of the appropriate ecdysteroid (0.1 mmol), in the appropriate anhydrous ketone (1.6 mL) under a nitrogen atmosphere. The reaction mixture was stirred at room temperature for 24–72 h until the conversion was found to be complete by TLC analysis and then diluted with ethyl acetate (5 mL) and quenched with a 5% aqueous solution of NaHCO_3_ (5 mL). The organic phase was washed with saturated aqueous NaCl, dried over anhydrous Na_2_SO_4_, filtered, and concentrated under reduced pressure to dryness. The resulting crude product was purified by FC on SiO_2_ as described below.

### General Procedure B for Esterification of Ecdysteroids (GP-B)

To a stirred solution of the appropriate ecdysteroid (0.1 mmol) in anhydrous pyridine (2.5 mL), under a nitrogen atmosphere at 0 °C, was slowly added over 5 min the appropriate carboxylic acid anhydride or chloride (0.5 mmol), and the resulting mixture was stirred at room temperature for 8–24 h until the conversion was found to be complete by TLC analysis. The resulting solution was dropped in 10 mL of 5% aqueous solution of H_3_PO_4_ and extracted twice with ethyl acetate (2 × 5 mL). The combined organic layers were washed with a 5% aqueous solution of NaHCO_3_ (2 × 5 mL), dried over anhydrous Na_2_SO_4_, filtered, and concentrated under reduced pressure to dryness. The resulting crude product was purified by FC on SiO_2_, as described below.

### General Procedure C for Esterification of Ecdysteroids (GP-C)

To a stirred solution of the appropriate ecdysteroid (0.1 mmol), *N,N′*-dimethylaminopyridine (49.2 mg, 0.4 mmol), and triethylamine (56 μL, 0.4 mmol), in anhydrous dichloromethane (2.5 mL) under a nitrogen atmosphere at 0 °C, was slowly added the appropriate carboxylic acid chloride (0.44 mmol) over a 5 min period. The resulting mixture was stirred at room temperature for 1–4 days, until the conversion was found to be complete by TLC analysis. The solution was dropped in 10 mL of a 5% aqueous H_3_PO_4_ solution and extracted twice with ethyl acetate (2 × 5 mL). The combined organic layers were dried over anhydrous Na_2_SO_4_, filtered, and concentrated under reduced pressure to dryness. The resulting crude product was purified by FC on SiO_2_ as described below.

### 20-Hydroxyecdysone-2,3,20,22-diacetonide (**6**)

This was prepared according to GP-A using 20-hydroxyecdysone (**1**) and acetone; FC: *n*-hexane–EtOAc, 1:1; yield: 53.2 mg, 97%; white solid. Spectroscopic data were in accordance with the literature.^13^

### Ponasterone-2,3,20,22-diacetonide (**7**)

This was prepared according to GP-A using ponasterone A (**2**) and acetone; FC: *n*-hexane–EtOAc, 1.5:1; yield: 52.0 mg, 95%; white solid. Spectroscopic data are in accordance with the literature.^13^

### 20-Hydroxyecdysone-2,3,20,22-dicyclopentyl-ketal (**8**)

This was prepared according to GP-A using 20-hydroxyecdysone (**1**) and cyclopentanone; FC: *n*-hexane–EtOAc, 1:1; yield: 41.0 mg, 67%; white solid; mp 108.3–109.5 °C; [α]^20^_D_ +44.8 (*c* 0.98, MeOH); ^1^H NMR (400 MHz, CDCl_3_) δ 5.83 (1H, d, br, *J* = 2.1 Hz), 4.20 (1H, m, br), 4.13 (1H, m, br), 3.55 (1H, d, br, *J* = 6.9 Hz), 2.83 (1H, t, br, *J* = 8.5 Hz), 2.80–240 (1H, m, br), 2.34 (1H, dd, *J* = 12.8 and 4.7 Hz), 2.25 (1H, m), 2.21–1.28 (32H, m), 1.25 (3H, s), 1.24 (3H, s), 1.19 (1H, m), 1.14 (3H, s, br), 0.98 (3H, s), 0.80 (3H, s); ^13^C NMR (75 MHz, CDCl_3_) δ 203.1, 168.4, 121.4, 118.1, 117.0, 84.9, 82.3, 76.6, 72.1, 71.6, 70.4, 50.8, 48.8, 47.4, 41.3, 38.4 (2C), 38.1, 37.9 (2C), 37.0, 34.2, 31.6, 30.9, 30.1, 29.2, 26.6, 23.9, 23.6, 23.4 (2C), 23.3 (2C), 21.2, 21.1, 20.4, 17.1; (+)-HRESIMS *m*/*z* 635.3911 [M + Na]^+^ (calcd for C_37_H_56_O_7_, 635.3918).

### 20-Hydroxyecdysone-2,3,20,22-dicyclohexyl-ketal (**9**)

This was prepared according to GP-A using 20-hydroxyecdysone (**1**) and cyclohexanone; FC: *n*-hexane–EtOAc, 1:1; yield: 59.5 mg, 93%; white solid; mp 265.6–266.9 °C; [α]^20^_D_ +34.0 (*c* 1.05, MeOH); ^1^H NMR (400 MHz, CDCl_3_) δ 5.82 (1H, d, br, *J* = 2.1 Hz), 4.25 (1H, m, br), 4.23 (1H, m, br), 3.68 (1H, d, br, *J* = 7.6 Hz), 2.82 (1H, t, br, *J* = 8.4 Hz), 2.35 (1H, dd, *J* = 12.8 and 4.7 Hz), 2.22 (1H, dd, *J* = 9.1 and 7.9 Hz), 2.15–1.94 (5H, m), 1.89–1.46 (28H, m), 1.45–1.29 (4H, m, br), 1.25 (3H, s), 1.24 (3H, s), 1.22 (1H, m), 1.16 (3H, s, br), 0.96 (3H, s), 082 (3H, s); ^13^C NMR (75 MHz, CDCl_3_) δ 202.9, 165.0, 121.5, 109.0, 107.6, 83.9, 81.8, 71.7, 77.0, 71.2, 70.3, 50.9, 49.0, 37.9, 41.5, 38.6 (2C), 38.5, 38.0, 36.1, 35.6, 34.3, 31.9, 30.0, 28.9, 26.8, 31.0, 25.2, 25.1, 24.2, 24.1, 23.8 (3C), 23.5, 20.5, 21.3, 22.2, 17.1; (+)-HRESIMS *m*/*z* 663.4222 [M + Na]^+^ (calcd for C_39_H_60_O_7_, 663.4231).

### 20-Hydroxyecdysone 2,3,22-triacetate (**10**)

This was prepared according to GP-B using 20-hydroxyecdysone (**1**) and acetic anhydride; FC: *n*-hexane–EtOAc, 1:9; yield: 48.5 mg, 80%; white solid. Spectroscopic data are in accordance with the literature.^13^

### Ponasterone A-2,3,22-triacetate (**11**)

This was prepared according to GP-B using ponasterone A (**2**) and acetic anhydride; FC: *n*-hexane–EtOAc, 1.5:1; yield: 46.0 mg, 78%; white solid; mp 156.1–157.0 °C; [α]^20^_D_ +54.3 (*c* 1.01, MeOH); ^1^H NMR (400 MHz, CDCl_3_) δ 5.90 (1H, d, br, *J* = 2.7 Hz), 5.39 (1H, m, br), 5.09 (1H, dt, br, *J* = 11.7 and 3.9 Hz), 4.86 (1H, dd, *J* = 10.6 and 2.1 Hz), 3.14 (1H, t, br, *J* = 8.8 Hz), 2.42 (1H, dd, *J* = 13.3 and 4.4 Hz), 2.39 (1H, t, *J* = 9.2 Hz), 2.22–1.30 (17H, m, methylene and OH protons), 2.14 (3H, s), 2.13 (3H, s), 2.04 (3H, s), 1.27 (3H, s), 1.19 (2H, q, br, *J* = 7.3 Hz), 1.06 (3H, s), 0.92 (3H, d, *J* = 6.5 Hz), 0.91 (3H, d, *J* = 6.5 Hz), 0.88 (3H, s); ^13^C NMR (101 MHz, CDCl_3_) δ 202.6, 173.1, 171.1, 170.8, 165.1, 122.2, 85.2, 80.0, 77.6, 69.2, 67.6, 51.5, 50.1, 48.1, 39.0, 36.3, 34.6, 34.2, 32.4, 31.6, 29.8, 28.6, 28.4, 24.4, 23.6, 22.8, 21.7 (4C), 21.1, 21.0, 18.1; (+)-HRESIMS *m*/*z* 613.3353 [M + Na]^+^ (calcd for C_33_H_50_O_9_, 613.3347).

### Makisterone A-2,3,22-triacetate (**12**)

This was prepared according to GP-B using makisterone A (**4**) and acetic anhydride; FC: *n*-hexane–EtOAc, 1:1; yield: 44.6 mg, 72%; white solid; mp 219.7–220.6 °C; [α]^20^_D_ +66.4 (*c* 1.00, MeOH); ^1^H NMR (400 MHz, CDCl_3_) δ 5.89 (1H, d, br, *J* = 2.4 Hz), 5.36 (1H, m, br), 5.08 (1H, dt, br, *J* = 11.6 and 3.7 Hz), 4.98 (1H, d, br, *J* = 10.1 Hz), 3.14 (1H, t, br, *J* = 8.2 Hz), 2.40 (1H, dd, *J* = 13.6 and 4.2 Hz), 2.36 (1H, t, *J* = 9.0 Hz), 2.26–1.14 (18H, m, methylene, methyne and OH protons), 2.13 (3H, s), 2.12 (3H, s), 2.02 (3H, s), 1.27 (3H, s), 1.21 (3H, s), 1.18 (3H, s), 1.05 (3H, s), 0.96 (3H, d, *J* = 6.6 Hz), 0.88 (3H, s); ^13^C NMR (75 MHz, CDCl_3_) δ 201.9, 172.6, 170.5, 170.2, 164.4, 121.6, 84.6, 77.3, 77.2, 72.9, 68.6, 67.0, 51.0, 49.4, 47.6, 40.6, 38.4, 34.1, 33.6, 32.0, 31.8, 31.1, 29.3, 27.8, 26.2, 23.8, 21.1 (3C), 21.0, 20.4 (2C), 17.5, 14.5; (+)-HRESIMS *m*/*z* 643.3446 [M + Na]^+^ (calcd for C_34_H_52_O_10_, 643.3453).

### Ecdysone 2,3,22-triacetate (**13**)

This was prepared according to GP-B using ecdysone (**3**) and acetic anhydride; FC: *n*-hexane–EtOAc, 1:1.5; yield: 52.0 mg, 88%; white solid; mp 121.3–122.1 °C; [α]^20^_D_ +58.5 (*c* 1.02, MeOH); ^1^H NMR (400 MHz, CDCl_3_) δ 5.89 (1H, d, br, *J* = 2.4 Hz), 5.37 (1H, m, br), 5.08 (1H, dt, br, *J* = 12.0 and 3.6 Hz), 4.90 (1H, dt, br, *J* = 9.7 and 2.6 Hz), 3.13 (1H, t, br, *J* = 8.2 Hz), 2.40 (1H, dd, *J* = 12.9 and 4.1 Hz), 2.24–1.30 (20H, m, methylene, methyne and OH protons), 2.12 (3H, s), 2.07 (3H, s), 2.02 (3H, s), 1.25 (3H, s), 1.24 (3H, s), 1.04 (3H, s), 0.96 (3H, d, *J* = 6.7 Hz), 0.68 (3H, s); ^13^C NMR (75 MHz, CDCl_3_) δ 202.8, 171.7, 171.2, 170.9, 165.1, 122.1, 85.1, 77.9, 71.4, 69.3, 67.7, 51.6, 48.0, 47.6, 41.1, 39.7, 39.1, 34.7, 34.4, 32.7, 31.2, 30.6, 29.9, 29.6, 26.1, 24.5, 22.6, 22.1, 21.8, 21.7, 21.1, 16.4, 14.1; (+)-HRESIMS *m*/*z* 613.3339 [M + Na]^+^ (calcd for C_33_H_50_O_9_, 613.3347).

### 20-Hydroxyecdysone 2,3,22-tribenzoate (**14**)

This was prepared according to GP-B using 20-hydroxyecdysone (**1**) and benzoyl chloride; FC: *n*-hexane–EtOAc, 1:1.5; yield: 69.0 mg, 87%; white solid; mp 148.5–149.0 °C; [α]^20^_D_ +15.7 (*c* 1.13, MeOH); ^1^H NMR (400 MHz, CDCl_3_) δ 8.10 (4H, d, br, *J* = 7.6 Hz), 7.88 (2H, d, br, *J* = 7.4 Hz), 7.64–7.55 (2H, m), 7.53–7.43 (5H, m), 7.33 (2H, t, br, *J* = 7.8 Hz), 5.99 (1H, d, br, *J* = 1.9 Hz), 5.73 (1H, m, br), 5.51 (1H, dt, br, *J* = 12.2 and 3.2 Hz), 5.19 (1H, d, br, *J* = 10.3 Hz), 4.05–2.99 (3H, m, br), 3.38 (1H, t, br, *J* = 7.9 Hz), 2.59 (1H, dd, *J* = 12.8 and 4.7 Hz), 2.53 (1H, m), 2.36–1.49 (16H, m), 1.45 (3H, s), 1.24 (3H, s), 1.23 (3H, s), 1.15 (3H, s, br), 0.93 (3H, s); ^13^C NMR (101 MHz, CDCl_3_) δ 202.4, 167.7, 165.9, 165.7, 165.2, 133.3 (2C), 133.1, 130.2, 130.1, 129.9, 129.8 (2C), 129.7 (2C), 129.6 (3C), 128.6 (2C), 128.4, 128.3 (2C), 121.6, 84.6, 80.6, 77.3, 70.8, 69.8, 68.2, 51.5, 49.7, 47.7, 40.3, 38.6, 34.6, 33.8, 31.3 (2C), 30.4, 29.4, 28.3, 25.0, 24.0, 21.6, 20.6 (2C), 17.5; (+)-HRESIMS *m*/*z* 815.3754 [M + Na]^+^ (calcd for C_48_H_56_O_10_, 815.3766).

### Ponasterone A-2,3,22-tribenzoate (**15**)

This was prepared according to GP-B using ponasterone A (**2**) and benzoyl chloride; FC: *n*-hexane–EtOAc, 1:1; yield: 56.5 mg, 73%; white solid; mp 118.8–119.7 °C; [α]^20^_D_ +18.3 (*c* 0.90, MeOH); ^1^H NMR (400 MHz, CDCl_3_) δ 8.15–8.06 (4H, m), 7.91 (2H, d, br, *J* = 7.8 Hz), 7.66–7.57 (2H, m), 7.54–7.44 (5H, m), 7.36 (2H, t, br, *J* = 7.8 Hz), 5.97 (1H, d, *J* = 2.2 Hz), 5.75 (1H, m, br), 5.48 (1H, dt, br, *J* = 12.0 and 3.3 Hz), 5.17 (1H, dd, br, *J* = 10.5 and 2.2 Hz), 3.34 (1H, t, br, *J* = 7.9 Hz), 2.60 (1H, dd, *J* = 13.2 and 3.9 Hz), 2.54–2.47 (1H, m), 2.35–1.50 (16H, m), 1.40 (3H, s), 1.33–1.22 (3H, m), 1.15 (3H, s, br), 0.93 (3H, s), 0.90 (6H, d, *J* = 6.6 Hz); ^13^C NMR (101 MHz, CDCl_3_) δ 201.9, 167.7, 165.9, 165.7, 164.7, 133.3, 133.2 (2C), 130.2 (2C), 129.9, 129.7 (4C), 129.6 (2C), 128.6 (2C), 128.5 (2C), 128.4 (2C), 121.7, 84.7, 80.0, 77.4, 69.7, 68.1, 51.4, 49.7, 47.6, 38.7, 35.7, 34.6, 33.8, 31.9, 31.2, 29.4, 28.2, 27.8, 24.0, 23.0, 22.2, 21.5, 20.6, 20.5, 17.6; (+)-HRESIMS *m*/*z* 799.3812 [M + Na]^+^ (calcd for C_48_H_56_O_9_, 799.3817).

### 20-Hydroxyecdysone 2,22-dihexanoate (**16**)

This was prepared according to GP-B using 20-hydroxyecdysone (**1**) and hexanoyl chloride; FC: *n*-hexane–EtOAc, 1:1.5; yield: 52.0 mg, 77%; white solid; mp 101.4–102.7 °C; [α]^20^_D_ +37.3 (*c* 1.02, MeOH); ^1^H NMR (400 MHz, CDCl_3_) δ 5.88 (1H, d, br, *J* = 2.4 Hz), 5.04 (1H, dt, br, *J* = 11.5 and 3.8 Hz), 4.88 (1H, dd, *J* = 10.3 and 2.2 Hz), 4.14 (1H, m, br), 3.13 (1H, t, br, *J* = 8.1 Hz), 2.53 (1H, dd, *J* = 13.5 and 4.1 Hz), 2.45–2.32 (5H, m), 2.21–1.21 (41H, m), 1.01 (3H, s), 0.95–089 (6H, m), 0.87 (3H, s); ^13^C NMR (101 MHz, CDCl_3_) δ 203.0, 175.3, 173.1, 164.3, 122.4, 84.7, 79.1, 76.5, 70.7, 68.6, 65.8, 49.9, 49.6, 47.5, 40.4, 39.2, 34.5 (2C), 33.7, 33.0, 31.7, 31.3 (2C), 31.1, 30.1, 29.4, 28.8, 24.8, 24.7, 24.5, 23.9, 22.3 (2C), 21.2, 20.6, 20.3, 17.5, 13.9 (2C); (+)-HRESIMS *m*/*z* 699.4449 [M + Na]^+^ (calcd for C_39_H_64_O_9_, 699.4443).

### 20-Hydroxyecdysone 2,22-dicinnamate (**17**)

This was prepared according to GP-B using 20-hydroxyecdysone (**1**) and cinnamoyl chloride; FC: *n*-hexane–EtOAc, 3:7; yield: 63.0 mg, 85%; white solid; mp 161.8–162.5 °C; [α]^20^_D_ +6.6 (*c* 0.77, MeOH); ^1^H NMR (400 MHz, CDCl_3_) δ 7.74 (1H, d, *J* = 15.9 Hz), 7.73 (1H, d, *J* = 15.9 Hz), 7.60–7.51 (4H, m), 7.45–7.38 (6H, m), 6.51 (1H, d, *J* = 15.9 Hz), 6.49 (1H, d, *J* = 15.9 Hz), 5.92 (1H, d, br, *J* = 2.4 Hz), 5.18 (1H, dt, br, *J* = 11.2 and 3,5 Hz), 5.04 (1H, dd, br, *J* = 10.3 and 1.7 Hz), 4.24 (1H, m, br), 3.21 (1H, t, br, *J* = 8.3 Hz), 2.58 (1H, dd, *J* = 13.2 and 3.9 Hz), 2.46 (1H, m), 2.21 (1H, dt, *J* = 13.2 and 4.4 Hz), 2.16–1.41 (19H, m), 1.37 (3H, s), 1.26 (3H, s), 1.24 (3H, s), 1.05 (3H, s, br), 0.91 (3H, s); ^13^C NMR (101 MHz, CDCl_3_) δ 203.1, 168.2, 166.3, 164.5, 145.7, 145.6, 134.3, 134.2, 130.5, 130.4, 129.0 (2C), 128.9 (2C), 128.2 (4C), 121.9, 117.9, 117.8, 84.7, 79.9, 77.2, 71.6, 70.7, 65.7, 50.0, 49.7, 47.6, 40.3, 38.6, 33.6, 33.1, 31.7, 31.3 (2C), 30.3, 28.7, 25.0, 23.8, 21.5, 20.6, 20.4, 17.5; (+)-HRESIMS *m*/*z* 763.3826 [M + Na]^+^ (calcd for C_45_H_56_O_9_, 763.3817).

### 20-Hydroxyecdysone 2,3,22,25-tetraacetate (**18**)

This was prepared according to GP-C using 20-hydroxyecdysone (**1**) and acetyl chloride; FC: *n*-hexane–EtOAc, 1:9; yield: 43.5 mg, 67%; white solid; mp 103.3–103.5 °C; [α]^20^_D_ +54.4 (*c* 1.05, MeOH); ^1^H NMR (400 MHz, CDCl_3_) δ 5.89 (1H, d, br, *J* = 2.4 Hz), 5.37 (1H, m, br), 5.09 (1H, dt, br, *J* = 11.8 and 3.7 Hz), 4.83 (1H, dd, br, *J* = 9.9 and 2.0 Hz), 3.14 (1H, t, br, *J* = 8.4 Hz), 2.40 (1H, dd, *J* = 13.4 and 4.2 Hz), 2.38 (1H, t, *J* = 8.8 Hz), 2.17 (1H, dd, *J* = 12.7 and 4.8 Hz), 2.15–1.47 (17H, m, methylene and OH protons), 2.14 (3H, s), 2.13 (3H, s), 2.02 (3H, s), 2.00 (3H, s), 1.46 (3H, s), 1.42 (3H, s), 1.27 (3H, s), 1.05 (3H, s), 0.87 (3H, s); ^13^C NMR (75 MHz, CDCl_3_) δ 202.1, 172.4, 170.6 (2C), 170.3, 164.6, 121.6, 84.5, 81.8, 79.4, 68.6, 67.0, 50.9, 49.6, 47.4, 38.3, 37.5, 34.0, 33.6, 31.6, 31.1, 29.2, 26.3, 25.9, 24.6, 23.8, 22.4, 21.1 (4C), 20.6, 20.4, 17.4; (+)-HRESIMS *m*/*z* [M + Na]^+^ 671.3408 (calcd for C_35_H_52_O_11_, 671.3402).

### Makisterone A-2,3,22,25-tetraacetate (**19**)

This was prepared according to GP-C using makisterone A (**4**) and acetyl chloride; FC: *n*-hexane–EtOAc, 1:1; yield: 43.0 mg, 65%; white solid; mp 111.1–111.9 °C; [α]^20^_D_ +62.3 (*c* 1.03, MeOH); ^1^H NMR (400 MHz, CDCl_3_) δ 5.90 (1H, d, br, *J* = 2.4 Hz), 5.38 (1H, m, br), 5.09 (1H, dt, br, *J* = 12.0 and 3.7 Hz), 4.95 (1H, d, br, *J* = 10.3 Hz), 3.14 (1H, m, br), 2.42 (1H, dd, *J* = 13.4 and 3.9 Hz), 2.35 (1H, t, *J* = 9.4 Hz), 2.22–1.10 (17H, m, methylene, methyne, and OH protons), 2.14 (3H, s), 2.13 (3H, s), 2.03 (3H, s), 2.00 (3H, s), 1.46 (3H, s), 1.37 (3H, s), 1.26 (3H, s), 1.06 (3H, s), 0.95 (3H, d, *J* = 6.8 Hz), 0.88 (3H, s, br); ^13^C NMR (75 MHz, CDCl_3_) δ 202.0, 172.8, 172.5, 170.5, 170.2, 164.4, 121.6, 84.9, 84.5, 77.0, 76.8, 68.6, 67.0, 50.9, 49.4, 47.5, 38.4, 37.2, 34.0, 33.6, 32.0, 31.8, 31.1, 29.2, 24.2, 23.8, 22.8, 22.6, 22.5, 21.1 (3C), 20.4 (2C), 17.5, 14.4; (+)-HRESIMS *m*/*z* 685.3564 [M + Na]^+^ (calcd for C_36_H_54_O_11_, 685.3558).

### Ecdysone 2,3,22,25-tetraacetate (**20**)

This was prepared according to GP-C using ecdysone (**3**) and acetyl chloride; FC: *n*-hexane–EtOAc, 1:1.5; yield: 48.5 mg, 77%; white solid; mp 101.1–101.9 °C; [α]^20^_D_ +58.3 (*c* 1.0, MeOH); ^1^H NMR (300 MHz, CDCl_3_) δ 5.90 (1H, d, br, *J* = 2.1 Hz), 5.39 (1H, m, br), 5.10 (1H, dt, br, *J* = 12.0 and 3.2 Hz), 4.87 (1H, m, br), 3.13 (1H, t, br, *J* = 8.6 Hz), 2.42 (1H, dd, *J* = 13.4 and 4.1 Hz), 2.20–1.14 (19H, m, methylene, methyne, and OH protons), 2.13 (3H, s), 2.08 (3H, s), 2.03 (3H, s), 2.01 (3H, s), 1.47 (3H, s), 1.46 (3H, s), 1.06 (3H, s), 0.96 (3H, d, *J* = 6.5 Hz), 0.69 (3H, s); ^13^C NMR (75 MHz, CDCl_3_) δ 202.7, 171.6, 171.2 (2C), 170.8, 164.8, 122.2, 85.2, 82.7, 77.6, 69.3, 67.6, 51.6, 48.0, 47.6, 39.5, 39.1, 38.2, 34.7, 34.4, 32.8, 31.2, 30.0, 26.9, 26.7, 26.0, 24.6, 22.4, 22.1, 21.8 (3C), 21.1, 16.4, 14.0; (+)-HRESIMS *m*/*z* 655.3458 [M + Na]^+^ (calcd for C_35_H_52_O_10_, 655.3453).

### Ajugasterone C-2,3,11,22-tetraacetate (**21**)

This was prepared according to GP-C using ajugasterone C (**5**) and acetyl chloride; FC: *n*-hexane–EtOAc, 1:9; yield: 59.0 mg, 91%; white solid; mp 106.4–107.3 °C; [α]^20^_D_ +44.6 (*c* 1.05, MeOH); ^1^H NMR (400 MHz, CDCl_3_) δ 5.94 (1H, d, br, *J* = 2.6 Hz), 5.42 (1H, m, br), 5.34–5.25 (2H, m, br), 4.83 (1H, dd, br, *J* = 10.5 and 1.8 Hz), 3.44 (1H, dd, *J* = 8.5 and 2.3 Hz), 2.44–2.33 (3H, m), 2.17–1.09 (16H, m, methylene and OH protons), 2.13 (3H, s), 2.12 (3H, s), 2.00 (3H, s), 1.97 (3H, s), 1.25 (3H, s), 1.12 (3H, s), 0.91 (3H, s), 0.90 (3H, d, *J* = 6.5 Hz), 0.89 (3H, d, *J* = 6.5 Hz); ^13^C NMR (101 MHz, CDCl_3_) δ 201.9, 173.1, 171.1, 170.9, 170.8, 161.9, 123.6, 84.6, 79.9, 77.4, 71.9, 69.2, 67.4, 52.2, 50.0, 47.9, 40.0, 38.9, 38.1, 36.5, 36.3, 32.3, 29.9, 28.6, 28.5, 24.5, 23.6, 22.8, 21.9, 21.8 (3C), 21.6, 21.2, 18.7; (+)-HRESIMS *m*/*z* 671.3412 [M + Na]^+^ (calcd for C_35_H_52_O_11_, 671.3402).

### 20-Hydroxyecdysone 2,3,22,25-tetrabenzoate (**22**)

This was prepared according to GP-C using 20-hydroxyecdysone (**1**) and benzoyl chloride; FC: *n*-hexane–EtOAc, 1:1.5; yield: 53.0 mg, 59%; white solid; mp 126.9–127.7 °C; [α]^20^_D_ +13.9 (*c* 1.04, MeOH); ^1^H NMR (400 MHz, CDCl_3_) δ 8.17–8.06 (4H, m), 8.01 (2H, d, br, *J* = 7.5 Hz), 7.91 (2H, d, br, *J* = 7.5 Hz), 7.67–7.39 (10H, m), 7.35 (2H, t, br, *J* = 7.6 Hz), 5.95 (1H, s, br), 5.70 (1H, m, br), 5.44 (1H, d, br, *J* = 11.6 Hz), 5.20 (1H, d, br, *J* = 10.0 Hz), 3.32 (1H, m, br), 2.64–2.37 (1H, m), 2.58 (1H, dd, *J* = 13.2 and 3.0 Hz), 2.51 (1H, m), 2.34–1.57 (17H, m), 1.62 (3H, s), 1.59 (3H, s), 1.41 (3H, s), 1.13 (3H, s, br), 0.91 (3H, s); ^13^C NMR (101 MHz, CDCl_3_) δ 202.0, 167.6, 165.9 165.7 (2C), 164.9, 133.3 (2C), 133.2, 132.7, 131.7, 130.2, 130.1, 129.9, 129.8 (2C), 129.7 (2C), 129.6 (2C), 129.5 (2C), 128.6 (3C), 128.4 (5C), 121.6, 84.4, 82.5, 80.1, 77.2, 69.7, 68.1, 51.4, 49.7, 47.6, 38.6, 38.5, 34.6, 33.7, 31.6, 31.2, 29.4, 26.3, 25.9, 25.0, 24.0, 21.6, 20.7, 20.5, 17.5; (+)-HRESIMS *m*/*z* 919.4019 [M + Na]^+^ (calcd for C_55_H_60_O_11_, 919.4028).

### 20-Hydroxyecdysone 2,3,22-trihexanoate (**23**)

This was prepared according to GP-C using 20-hydroxyecdysone (**1**) and hexanoyl chloride; FC: *n*-hexane–EtOAc, 1:1.5; yield: 52.5 mg, 68%; white solid; foam; [α]^20^_D_ +31.4 (*c* 0.99, MeOH); ^1^H NMR (400 MHz, CDCl_3_) δ 5.89 (1H, d, br, *J* = 2.0 Hz), 5.39 (1H, m, br), 5.10 (1H, dt, br, *J* = 12.0 and 3.5 Hz), 4.88 (1H, d, br, *J* = 10.5 Hz), 3.15 (1H, t, br, *J* = 8.1 Hz), 2.62–2.31 (7H, m), 2.27–1.17 (47H, m), 1.05 (3H, s), 0.98–088 (9H, m), 0.87 (3H, s); ^13^C NMR (101 MHz, CDCl_3_) δ 202.8, 176.1, 173.9, 173.6, 165.1, 122.3, 85.3, 80.1, 77.8, 71.3, 69.2, 67.4, 51.7, 50.2, 48.2, 41.0, 39.1, 35.2, 35.1, 34.9, 34.8, 34.2, 32.4, 32.0, 31.9 (2C), 31.8, 30.8, 30.0, 29.5, 25.5, 25.4 (2C), 25.1, 24.5, 23.0 (3C), 21.9, 21.2, 21.0, 18.2, 14.6 (3C); (+)-HRESIMS *m*/*z* 797.5166 [M + Na]^+^ (calcd for C_45_H_74_O_10_, 797.5174).

### 20-Hydroxyecdysone 2,3,22-trilaurate (**24**)

This was prepared according to GP-C using 20-hydroxyecdysone (**1**) and lauryl chloride; FC: *n*-hexane–EtOAc, 1:1.5; yield: 58.5 mg, 57%; colorless thick oil; [α]^20^_D_ +25.0 (*c* 0.55, MeOH); ^1^H NMR (400 MHz, CDCl_3_) δ 5.89 (1H, d, br, *J* = 2.0 Hz), 5.39 (1H, m, br), 5.10 (1H, dt, br, *J* = 12.3 and 3.5 Hz), 4.88 (1H, d, br, *J* = 10.5 Hz), 3.15 (1H, t, br, *J* = 7.9 Hz), 2.44–2.32 (8H, m), 2.25 (1H, t, *J* = 7.7 Hz), 2.20–1.25 (75H, m), 1.25 (3H, s), 1.23 (3H, s), 1.04 (3H, s), 0.99 (9H, t, *J* = 6.8 Hz), 0.87 (3H, s); ^13^C NMR (101 MHz, CDCl_3_) δ 202.7, 176.1, 173.8, 173.5, 165.1, 122.3, 85.2, 80.1, 77.7, 71.3, 69.2, 67.4, 51.7, 50.2, 48.2, 41.0, 39.1, 35.3, 35.2, 35.0, 34.9, 34.2, 32.6 (3C), 32.4, 31.7, 30.8, 30.3–29.8 (19C), 29.5, 25.8 (2C), 25.5, 25.4 (1C), 24.5, 23.4 (3C), 21.9, 21.2, 21.0, 18.1, 14.8 (3C); (+)-HRESIMS *m*/*z* 1049.7998 [M + Na]^+^ (calcd for C_63_H_110_O_10_, 1049.7991).

### Ponasterone A-2,3,22-trilaurate (**25**)

This was prepared according to GP-C using ponasterone A (**2**) and lauryl chloride; FC: *n*-hexane–EtOAc, 1:1.5; yield: 60.0 mg, 59%; colorless thick oil; [α]^20^_D_ +15.4 (*c* 0.51, MeOH); ^1^H NMR (400 MHz, CDCl_3_) δ 5.90 (1H, d, br, *J* = 2.4 Hz), 5.39 (1H, m, br), 5.11 (1H, dt, br, *J* = 12.0 and 3.5 Hz), 4.87 (1H, dd, *J* = 10.5 and 2.0 Hz), 3.16 (1H, t, br, *J* = 8.3 Hz), 2.45–2.33 (6H, m), 2.26 (2H, t, br, *J* = 7.6 Hz), 2.21–1.14 (76H, m), 1.05 (3H, s), 0.91 (6H, d, *J* = 6.6 Hz), 0.90 (9H, t, *J* = 6.9 Hz), 0.88 (3H, s); ^13^C NMR (101 MHz, CDCl_3_) δ 202.0, 17.5, 173.2, 172.9, 164.3, 121.7, 84.7, 79.1, 77.2, 68.5, 66.7, 51.1, 49.6, 47.5, 38.5, 35.7, 34.5, 34.2, 33.9 (2C), 33.6, 31.9 (4C), 31.1, 29.7–29.1 (20C), 27.7, 25.1, 24.7 (2C), 23.9, 26.6, 22.7 (3C), 22.2, 21.1, 20.5, 20.4, 17.5, 14.1 (3C); (+)-HRESIMS *m*/*z* 1033.8031 [M + Na]^+^ (calcd for C_63_H_110_O_9_, 1033.8042).

### 20-Hydroxyecdysone 2,3,22-tricinnamate (**26**)

This was prepared according to GP-C using 20-hydroxyecdysone (**1**) and cinnamoyl chloride; FC: *n*-hexane–EtOAc, 3:7; yield: 48.0 mg, 55%; white solid; mp 157.2–158 °C; [α]^20^_D_ – 47.9 (*c* 1.01, MeOH); ^1^H NMR (400 MHz, CDCl_3_) δ 7.74 (1H, d, *J* = 15.9 Hz), 7.73 (1H, d, *J* = 15.9 Hz), 7.65 (1H, d, *J* = 16.1 Hz), 7.59–7.53 (4H, m), 7.50–7.45 (2H, m), 7.44–7.33 (9H, m), 6.54 (1H, d, *J* = 15.9 Hz), 6.51 (1H, d, *J* = 15.9 Hz), 6.37 (1H, d, *J* = 16.1 Hz), 5.93 (1H, d, br, *J* = 2.4 Hz), 5.60 (1H, m, br), 5.29 (1H, dt, br, *J* = 11.7 and 3,6 Hz), 5.05 (1H, dd, br, *J* = 10.7 and 1.7 Hz), 3.24 (1H, t, br, *J* = 9.4 Hz), 2.53 (1H, dd, *J* = 13.2 and 3.9 Hz), 2.47 (1H, m), 2.27–1.39 (19H, m), 1.36 (3H, s), 1.26 (3H, s), 1.25 (3H, s), 1.11 (3H, s, br), 0.91 (3H, s); ^13^C NMR (101 MHz, CDCl_3_) δ 202.3, 168.3, 166.2, 166.1, 164.9, 145.7, 145.5 (2C), 134.3, 134.2 (2C), 130.5 (2C), 130.4, 129.0 (4C), 128.9 (2C), 128.2 (6C), 121.6, 118.0, 117.9, 117.8, 84.5, 80.1, 77.1, 70.7, 69.1, 67.5, 51.2, 49.7, 47.8, 40.3, 38.6, 34.4, 33.7, 31.5, 31.2, 30.3, 29.4, 28.6, 25.0, 23.9, 21.5, 20.6, 20.5, 17.5; (+)-HRESIMS *m*/*z* 893.4229 [M + Na]^+^ (calcd for C_54_H_62_O_10_, 893.4235).

### 20-Hydroxyecdysone 2,3,22-trioleate (**27**)

This was prepared according to GP-C using 20-hydroxyecdysone (**1**) and oleyl chloride; FC: *n*-hexane–EtOAc, 1:1.5; yield: 56.0 mg, 44%; pale yellow thick oil; [α]^20^_D_ +5.88 (*c* 1.08, MeOH); ^1^H NMR (400 MHz, CDCl_3_) δ 5.89 (1H, d, br, *J* = 2.1 Hz), 5.44–5.30 (7H, m, br), 5.10 (1H, dt, br, *J* = 12.3 and 3.4 Hz), 4.88 (1H, d, br, *J* = 8.8 Hz), 3.15 (1H, t, br, *J* = 8.1 Hz), 2.44–2.32 (6H, m, br), 2.24 (2H, t, *J* = 7.4 Hz), 2.21–1.26 (100H, m), 1.25 (3H, s), 1.23 (3H, s), 1.04 (3H, s, br), 0.89 (9H, t, *J* = 7.1 Hz), 0.87 (3H, s); ^13^C NMR (101 MHz, CDCl_3_) δ 202.7, 173.8, 173.5, 176.0, 165.1, 130.7 (3C), 130.4 (3C), 122.3, 85.2, 80.1, 77.8, 71.3, 69.2, 67.4, 51.7, 50.2, 48.2, 41.0, 39.1, 35.2, 35.1, 35.0, 34.9, 34.2, 32.3, 31.8, 30.9, 30.5–29.8 (30C), 30.2, 29.4, 27.9 (6C), 25.8 (3C), 25.4, 24.5, 21.9, 21.2, 21.1, 18.1, 14.8 (3C); (+)-HRESIMS *m*/*z* 1296.0348 [M + Na]^+^ (calcd for C_81_H_140_O_10_, 1296.0339).

### 20-Hydroxyecdysone 2,3,22-tri(2-(1*H*-indol-3-yl)acetate (**28**)

This was prepared as follows: *N,N′*-Dicyclohexylcarbodiimide (144 mg, 0.7 mmol) was added in portions to a solution of 2-(1*H*-indol-3-yl)acetic acid (123 mg, 0.7 mmol) in dry dioxane (3 mL) under a nitrogen atmosphere. After 1 h, the white solid was filtered off and the filtrate was added to a suspension of 20-hydroxyecdysone (**1**) (48 mg, 0.1 mmol) in dry dioxane (0.5 mL) and *N,N′*-dimethylaminopyridine (6.0 mg, 0.05 mmol). The reaction mixture was stirred, at 40 °C, for 22 h under a nitrogen atmosphere and then diluted with EtOAc (10 mL). The resulting solution was washed with a 5% aqueous NaHCO_3_, 5% aqueous H_3_PO_4_, and then brine. The organic phase was dried over Na_2_SO_4_, filtered, and concentrated under reduced pressure to dryness. The resulting crude product was purified by flash column chromatography on SiO_2_ (*n*-hexane–EtOAc, 1.5:1); yield: 79.0 mg, 83%; pale yellow solid; mp 157–168 °C; [α]^20^_D_ +26.4 (*c* 1.00, MeOH); ^1^H NMR (400 MHz, CDCl_3_) δ 8.38 (2H, s, br), 8.29 (1H, s, br), 7.65 (1H, d, *J* = 7.7 Hz), 7.59 (1H, d, *J* = 7.8 Hz), 7.49 (1H, d, *J* = 7.8 Hz), 7.36–7.28 (3H, m), 7.23–7.09 (7H, m), 7.04 (1H, s), 6.93 (1H, s), 5.73 (1H, d, br, *J* = 1.8 Hz), 5.33 (1H, m, br), 5.00 (1H, d, br, *J* = 12.3 Hz), 4.80 (1H, d, br, *J* = 10.2 Hz), 3.85 (1H, d, *J* = 15.2 Hz), 3.81 (1H, d, *J* = 15.2 Hz), 3.72 (1H, d, *J* = 15.9 Hz), 3.69 (1H, d, *J* = 15.9 Hz), 3.51 (2H, s, br), 3.01 (1H, t, br, *J* = 9.1 Hz), 2.29–2.21 (1H, m), 2.17 (1H, dd, *J* = 12.9 and 3.8 Hz), 2.01–1.05 (19H, m, methylene and OH protons), 1.16 (3H, s), 1.02 (3H, s), 0.96 (3H, s), 0.83 (3H, s), 0.72 (3H, s); ^13^C NMR (101 MHz, CDCl_3_) δ 202.9, 174.1, 172.3, 172.2, 165.2, 136.8, 136.7, 136.5, 127.9 (2C), 127.8, 124.0, 123,8, 123.7, 123.0, 122.8, 122.7, 122.1, 120.4, 120.3, 120.2, 119.6, 119.5, 119.4, 112.0–111.9 (3C), 109.1, 108.9, 108.5, 84.9, 80.7, 77.4, 71.1, 69.9, 68.2, 51.5, 50.1, 48.0, 40.5, 38.8, 34.4, 34.2, 32.5, 32.0, 31.9, 31.7 (2C), 30.4, 29.8, 29.0, 25.4, 24.2, 21.9, 21.1, 21.0, 18.0; (+)-HRESIMS *m*/*z* 974.4558 [M + Na]^+^ (calcd for C_57_H_65_ N_3_O_10_, 974.4562).

### Cell Cultures

The CCRF-CEM and LoVo cell lines were purchased from the American Type Culture Collection and were grown in RPMI and Ham’s F12, respectively. CEM^Vbl100^ and LOVO^Doxo^ cells in RPMI were a kind gift of Dr. S. Arancia (Istituto Superiore di Sanità, Rome, Italy). CEM^Vbl-100^ are a multi-drug-resistant line selected against vinblastine,^35^ and they grow in RPMI in the presence of 100 ng/mL vinblastine. LoVo^Doxo^ cells are a doxorubicin-resistant subclone of LoVo cells^36^ and were grown in complete Ham’s F12 medium supplemented with doxorubicin (100 ng/mL). All media were supplemented with 10% fetal bovine serum (FBS), glutamine (2 mM), penicillin (100 U/mL), and streptomycin (100 μg/mL) (all from Thermo Fisher Scientific, Waltham, MA, USA).

The medulloblastoma cell line DAOY was purchased from the American Type Culture Collection. Cells were cultured in RPMI 1640 or αMEM (Life Technologies, Italy) supplemented with 10% FBS, glutamine (2 mM; Life Technologies, Italy), penicillin (100 U/mL; Life Technologies, Italy), and streptomycin (100 μg/mL; Life Technologies, Italy) and maintained at 37 °C in a humidified atmosphere with 5% CO_2_.

### Evaluation of Cytotoxicity in Peripheral Blood Lymphocytes

Peripheral blood lymphocytes were obtained from human peripheral blood (leucocyte-rich plasma-buffy coats) from healthy donors using Lymphoprep (Fresenius KABI Norge AS) gradient density centrifugation. Buffy coats were collected and provided by the Blood Transfusion Service of Azienda Ospedaliera di Padova only for research purposes, without identifier. The samples were not obtained specifically for this study, and for this reason ethical approval was not required. Informed consent was obtained from blood donors according to Italian law no. 219 (October 21, 2005). Data have been treated by the Blood Transfusion Service according to Italian law on personal management “Codice in materia di protezione dati personali” (Testo Unico D.L. giugno 30, 2003 196). The experimental procedures were carried out in strict accordance with approved guidelines.

After extensive washing, cells were resuspended (1.0 × 10^6^ cells/mL) in RPMI-1640 with 10% FBS and incubated overnight. For cytotoxicity evaluations in proliferating PBL cultures, nonadherent cells were resuspended at 5 × 10^5^ cells/mL in growth medium, containing 2.5 μg/mL PHA (Irvine Scientific). Different concentrations of the test compounds were added, and viability was determined 72 h later by the MTT test. For cytotoxicity evaluations in resting PBL cultures, nonadherent cells were resuspended (5 × 10^5^ cells/mL) and treated for 72 h with the test compounds.

### Flow Cytometric Analysis of Rhodamine 123

Functional activity of P-glycoprotein was measured with the fluorescent dye Rho123 (Pierce, Rockford IL, USA), which is a substrate of P-gp. Briefly, after different times of treatment, the cells were collected by centrifugation and resuspended in Hank’s balanced salt solution (HBSS) containing 0.1 μM Rho 123. The cells were then incubated for 20 min at 37 °C, centrifuged, and resuspended in HBSS. The fluorescence was directly recorded by flow cytometry with a Coulter Cytomics FC500 (Beckman Coulter).

### Flow Cytometric Analysis of Side Population

The protocol of SP analysis was based on Goodell and co-workers.^37^ Briefly, cells (10^6^/mL) were incubated in αMEM containing 2% FBS (Life Technologies) and 5 μg/mL Hoechst 33342 dye (Sigma-Aldrich) for 90 min at 37 °C, either alone or in the presence of 50 μM verapamil (Sigma). At the end of incubation, cells were washed and then incubated in PBS supplemented with 2% FCS and 2 μg/mL propidium iodide (Sigma), at 4 °C for 10 min, to discriminate dead cells. The cells were then analyzed in a MoFlo XDP (Beckman Coulter, USA) equipped with a 355 UV laser to measure both Hoechst blue fluorescence and Hoechst red fluorescence. Based on the Hoechst double emission the SP profile appears as a small fraction of cells forming a tail extending from non-SP populations. A gate on PI-negative cells was used to exclude dead cells, and side population was analyzed on a Hoechst red vs Hoechst blue plot: if present, the SP appears as a dim tail with respect to non-SP. A minimum of 30 000 live cells events was acquired.

### P-Glycoprotein Activity Assay

P-gp ATPase activity after ecdysteroid treatment was estimated by a P-gp-Glo assay system (Promega, Madison, WI, USA) following the manufacturer’s instructions. This method relies on the ATP dependence of the light-generating reaction of firefly luciferase, where ATP consumption is detected as a decrease in luminescence. In a 96-well plate, recombinant human P-gp (25 μg) was incubated with P-gp-Glo assay buffer (20 μL), verapamil (200 μM) as positive control, sodium orthovanadate (100 μM) as a P-gp ATPase inhibitor, and the test compounds (1–10 μM). The reaction was initiated by addition of MgATP (10 mM), then stopped 40 min later by addition of 50 μL of firefly luciferase reaction mixture (ATP detection reagent), which initiated an ATP-dependent luminescence reaction. Signals were measured 60 min later by a Victor^3TM^ 1420 multilabel counter (PerkinElmer, Waltham, MA, USA).

### Drug Treatment

Cells were grown to 60% confluence and then treated with test compounds at a stock concentration of 10 mM. Cells were treated for 72 h using scalar dilutions of P-gp inhibitors **9** and **14**, combined with vinblastine, doxorubicin, vincristine at a stock concentration of 10 mM, and cisplatin at a stock concentration of 5 mM (Sigma-Aldrich) and then used at different concentrations. Doxorubicin, vincristine, and cisplatin were added to drug solutions at fixed combination ratios, while vinblastine was added at fixed concentrations of 1 μM. The effectiveness of various drug combinations was analyzed by the Calcusyn version 2.1 software (Biosoft). The combination index was calculated according to the Chou–Talalay method.^19,20^ A combination index of 1 indicates an additive effect of the two drugs. Combination index values less than 1 indicate synergy, and combination index values more than 1 indicate antagonism.

### MTT Assay

Proliferation was assessed by an MTT ((3-(4,5-dimethylthiazol-2-yl)-2,5-diphenyl tetrazolium bromide) assay after treatment. Equal concentrations of cells were plated in triplicate in a 96-well plate and incubated with 10 μL of MTT (Sigma-Aldrich, St. Louis, MO, USA) for 4 h. Absorbance was measured at 560 nm using a Victor3 1420 multilabel counter (PerkinElmer). The growth inhibition (GI_50_ = compound concentration required to inhibit cell proliferation by 50%) was calculated by plotting the data as a logarithmic function of (*x*) when viability was 50%. DMSO-treated cell viability was set to 100%.^38^

### RNA Isolation and Reverse Transcriptase Polymerase Chain Reaction

Total cellular RNA from cell lines and patient bone marrow was extracted with TRIzol reagent (Invitrogen). RNA quality was controlled using a Nanodrop spectrophotometer. Subsequently, 1 μg of total RNA was reversely transcribed using random hexamers and Superscript II (Invitrogen), according to the manufacturer’s instructions.

### Real-Time PCR

Real-time quantitative PCR was performed on an Applied Biosystems 7900 HT sequence detection system using SYBR Green PCR master mixture reagents (Applied Biosystems; Forest City, CA, USA). Primer used for analysis of the ABCB1 gene (p-glycoprotein) F: 5′-CACCAAGGCCCTGCGCTACC-3′, R: 5′-ACACCCGGTACCCGCGATGA-3′ and for the GUS gene F: 5′-GAAAATATGTGGTTGGAGAGC-3′, R: 5′-CGAGTGAAGATCCCCTTTTTA-3′.

### Molecular Docking

A homology model of the human P-glycoprotein was built on the crystal structure of mouse P-glycoprotein; PDB code: 3G60. The model was generated with the Yasara software with the default parameters (www.yasara.org). The selected ligands were first submitted to a Monte Carlo conformational search with the MMFF94 force field in vacuo with Spartan ’08.^39^ The obtained conformers were used for docking studies. Docking was performed using AutoDock^40^ using the default docking parameters supplied with AutoDock in the “examples” subdirectory, and point charges were initially assigned according to the AMBER03 force field^41^ and then damped to mimic the less polar Gasteiger charges used to optimize the AutoDock scoring function. The setup was done with the YASARA molecular modeling program.^42^ For each ligand 75 Autodock LGA runs were executed. Results were sorted by binding energy (more positive energies indicate stronger binding, and negative energies mean no binding). After clustering the 75 runs, the resulting complex conformations were originated and clustered (they all differed by at least 5.0 A heavy atom RMSD). Binding energies are reported in kcal/mol, and predicted dissociation constants in pM units. Contacting receptor residues are also listed. After the clustering the energy spread [average and standard deviation] was calculated: the dissociation constant has been recalculated from the average binding energy.^43^

### Statistical Analysis

All experiments were performed with a minimum of three technical and three biological replicates, and values reported are the mean of the three biological replicates, unless otherwise indicated. Error bars represent the standard error of the mean (SEM), unless otherwise indicated. All statistical analyses were performed using the GraphPad Prism software (version 7.0).

## Supporting Information Available

^1^H NMR and ^13^C NMR spectra of all synthesized compounds (PDF)
