# Supplementary material for: Ecdysteroid Derivatives that Reverse P-Glycoprotein-Mediated Drug Resistance
Source: J Nat Prod. 2020 Aug 13;83(8):2434–46. doi: 10.1021/acs.jnatprod.0c00334 (PMC8009596; doi:10.1021/acs.jnatprod.0c00334)

Supporting Information

for

Novel ecdysteroids derivatives significantly reverse P-glycoprotein mediated multidrug resistance

[1]*Dipartimento di Salute della Donna e del Bambino, Laboratorio di Oncoematologia, Università degli Studi di Padova via Giustiniani 2, Padova, 35128, Italy.*

[2]*Istituto di Ricerca Pediatrica (IRP) Corso Stati Uniti 4, Padova 35129, Italy*

[3]*Dipartimento di Chimica, Università di Milano via Golgi 19, Milano, 20133, Italy.,*

[4]*Politecnico di Milano, Dipartimento di Chimica, Materiali ed Ing. Chimica 'Giulio Natta', Piazza Leonardo da Vinci 32, Milano, 20133, Italy*

Table of contents

^{1}H and ^{13}C NMR spectra for all new compounds

Compound 8: ^{1}H NMR (400 MHz, $CDCl_3$)

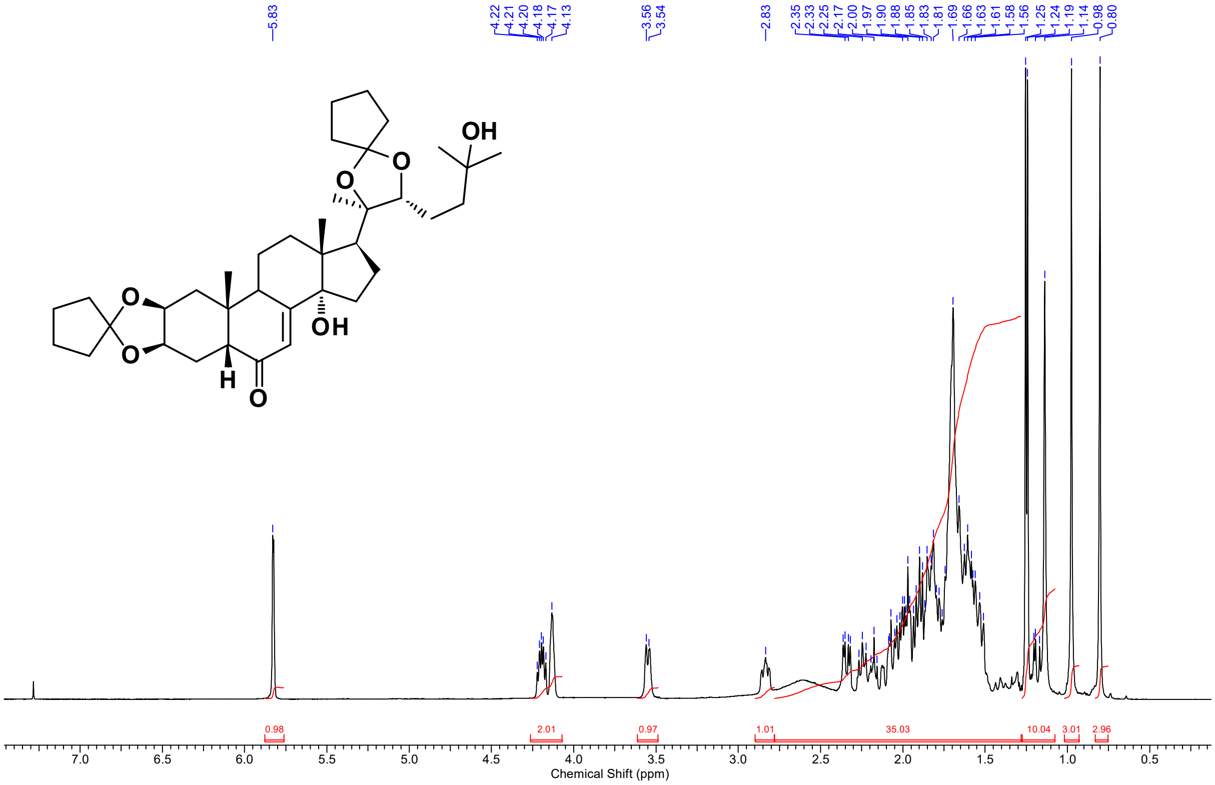

Compound 8: ^{13}C NMR (75 MHz, $CDCl_3$)

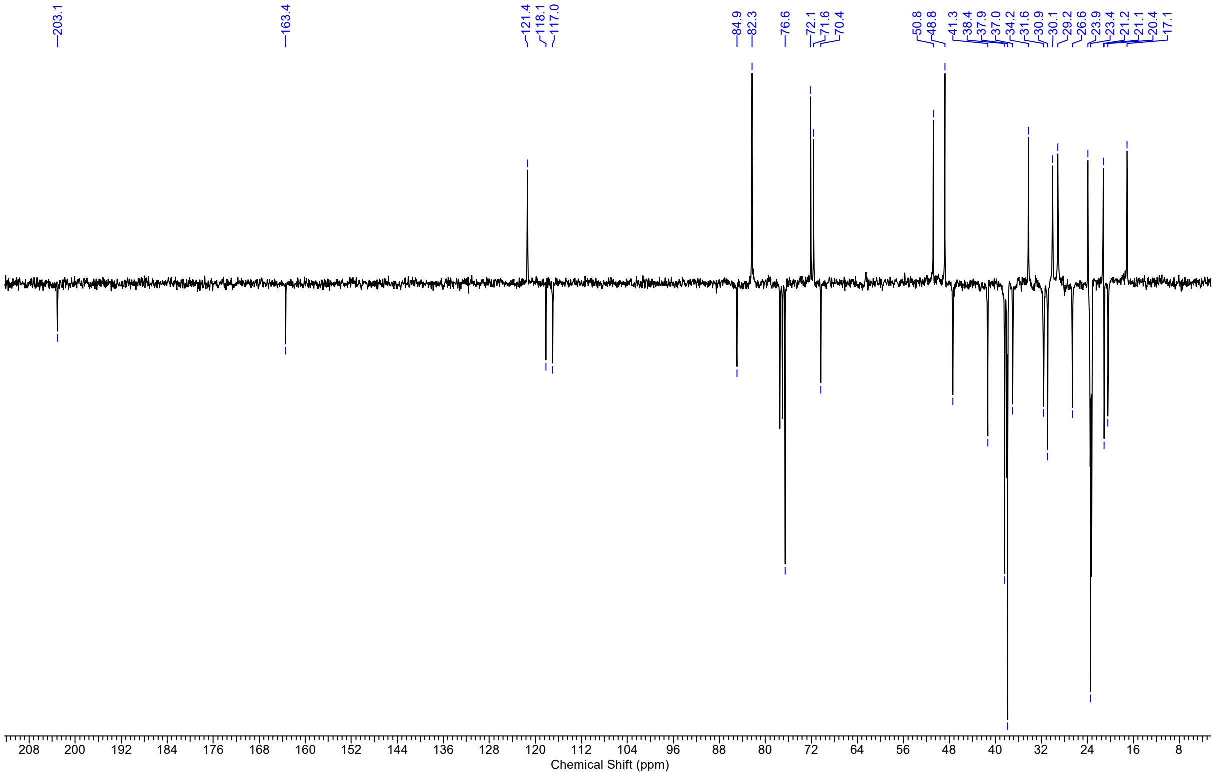

Compound 9: ^{1}H NMR (400 MHz, $CDCl_3$)

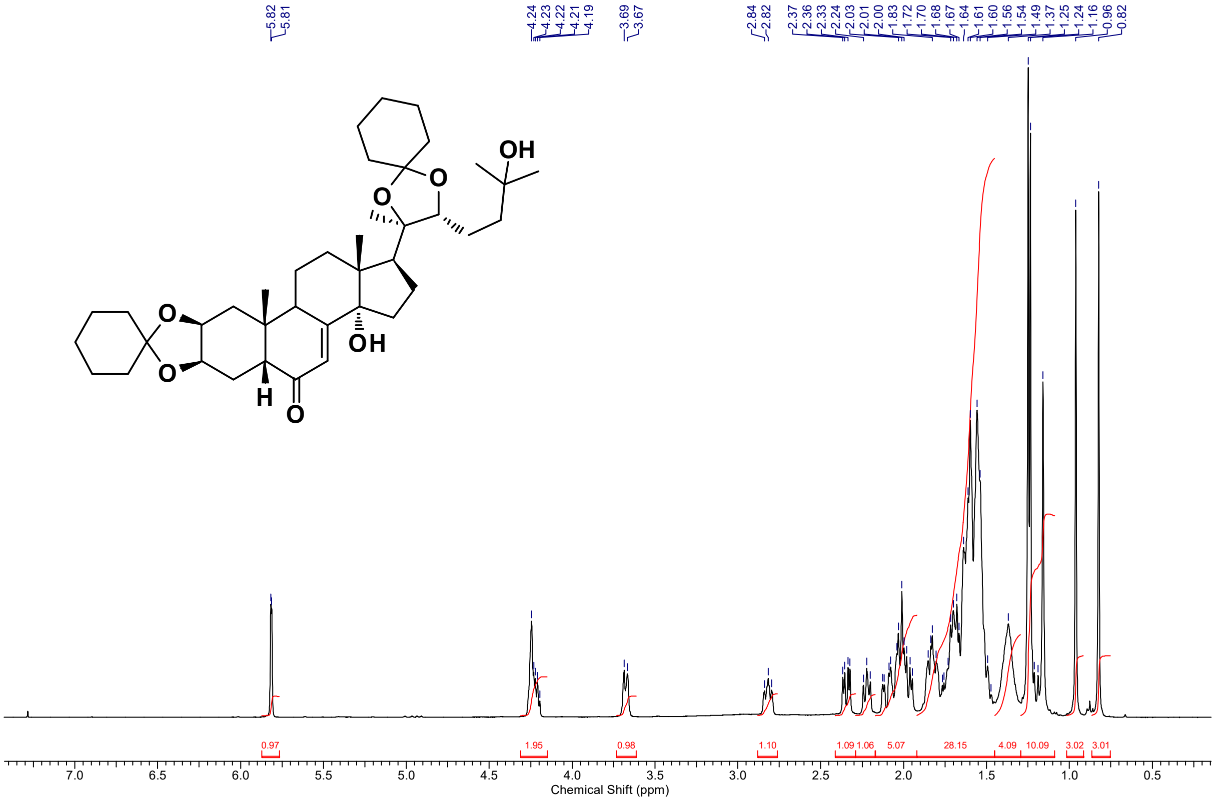

Compound 9: ^{13}C NMR (75 MHz, $CDCl_3$)

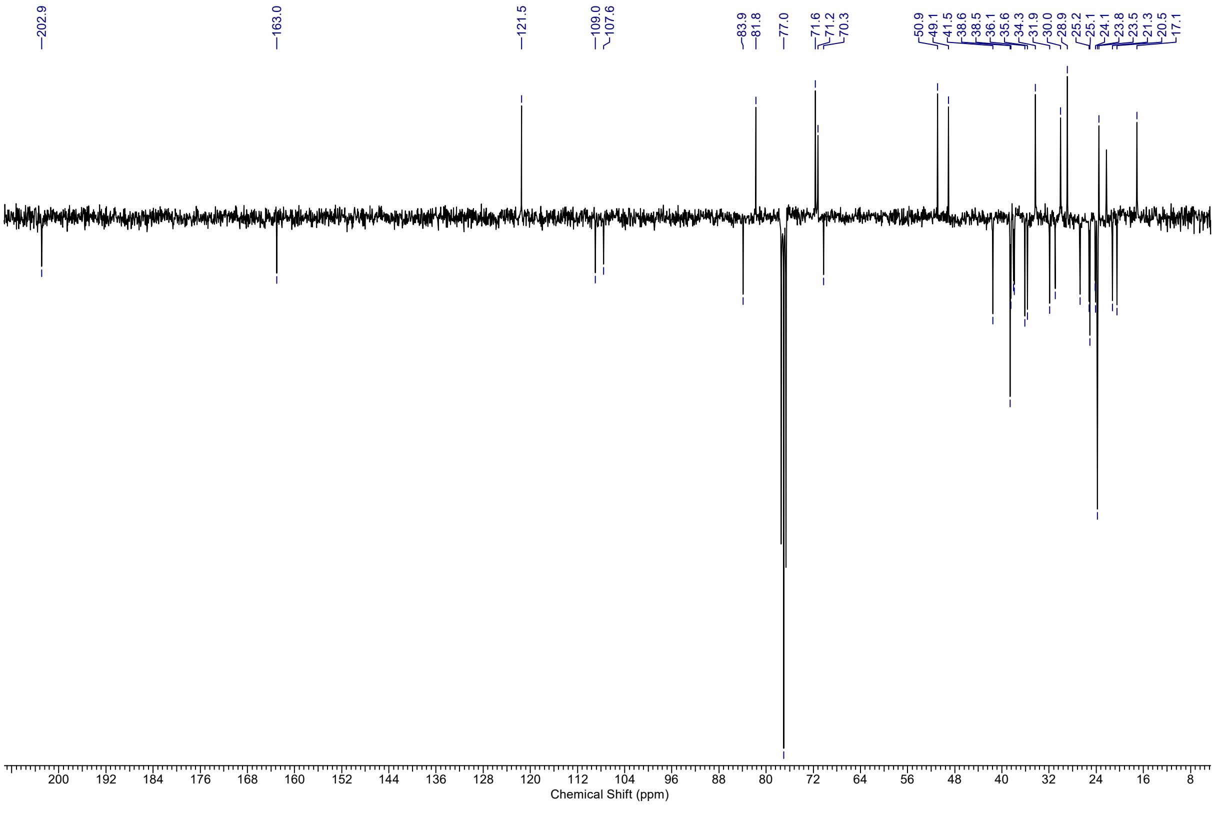

Compound 11: ^{1}H NMR (400 MHz, $CDCl_3$)

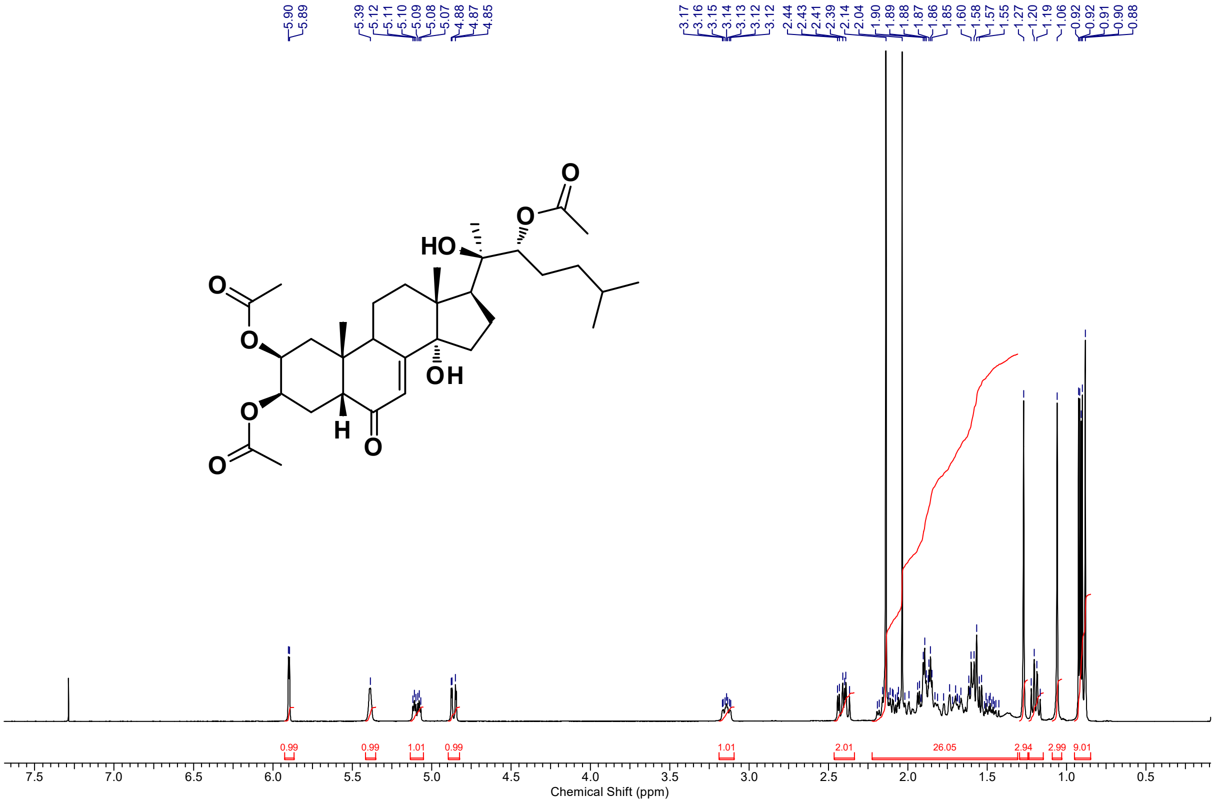

Compound 11: ^{13}C NMR (101 MHz, $CDCl_3$)

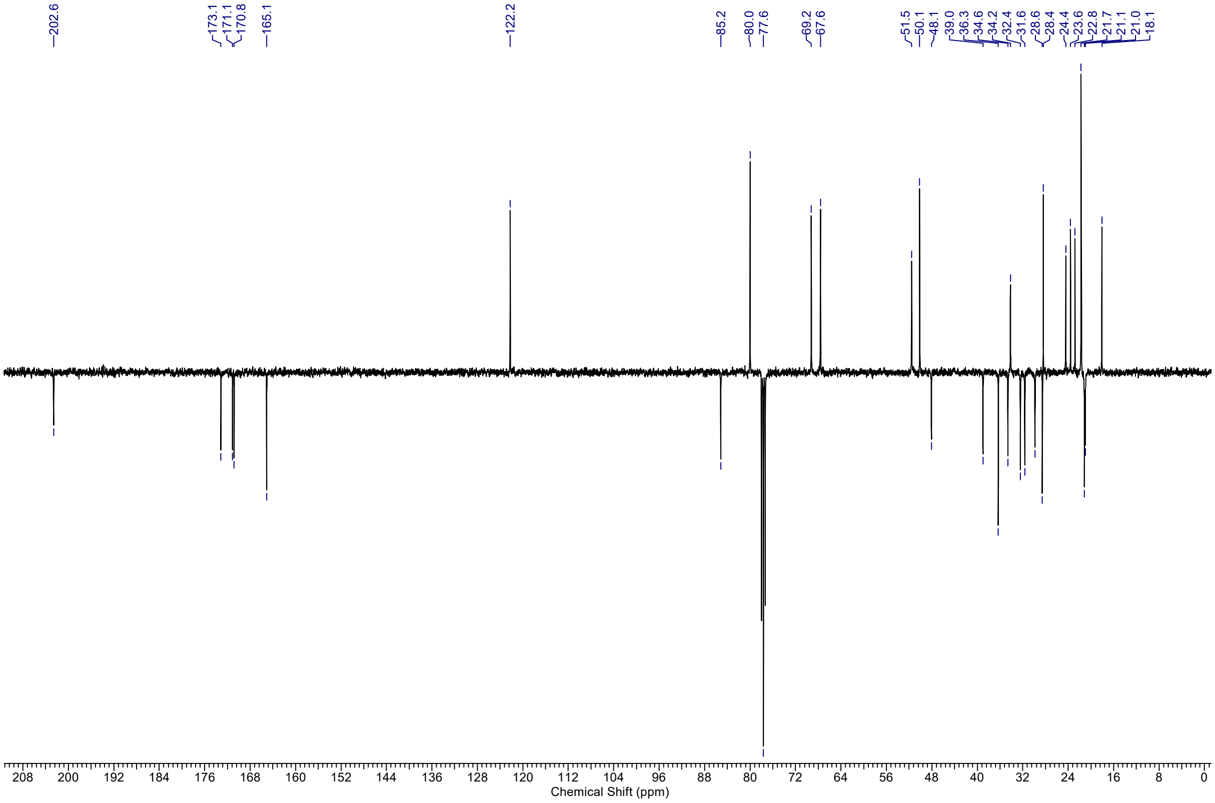

Compound 12: ^{1}H NMR (400 MHz, $CDCl_3$)

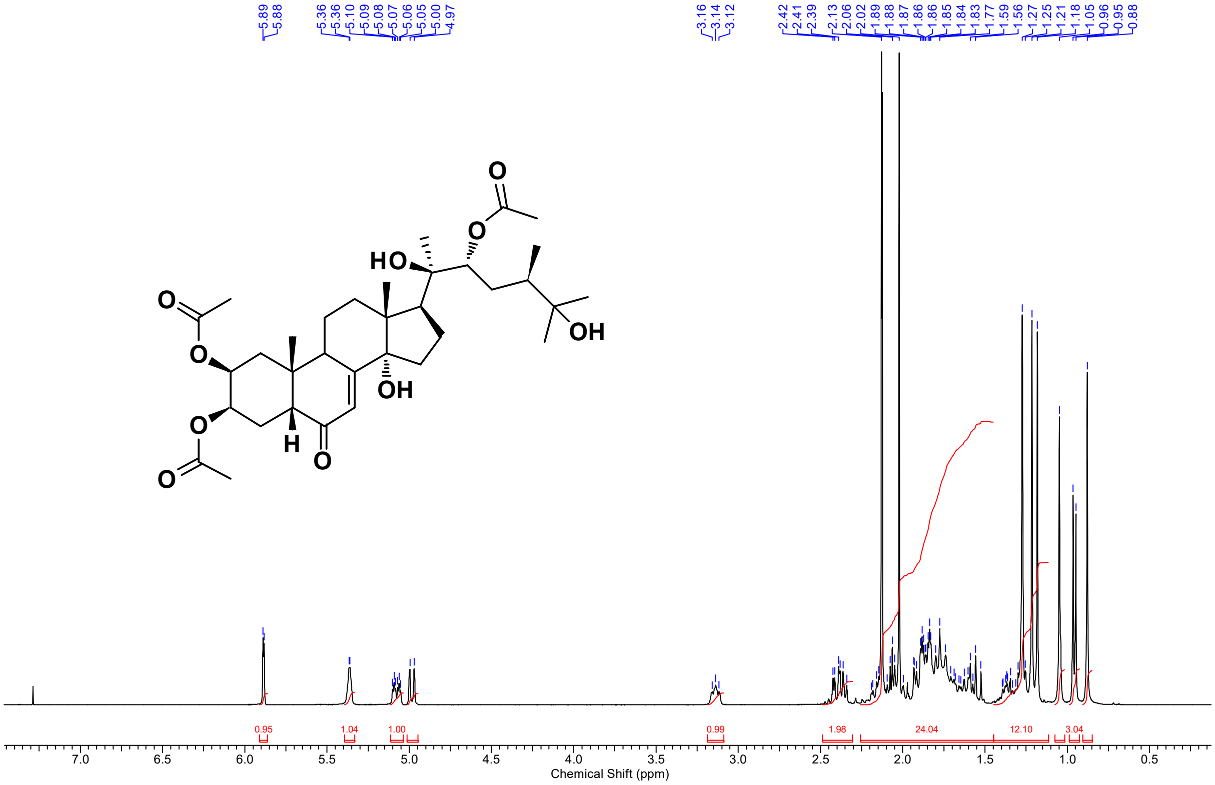

Compound 12: ^{13}C NMR (101 MHz, $CDCl_3$)

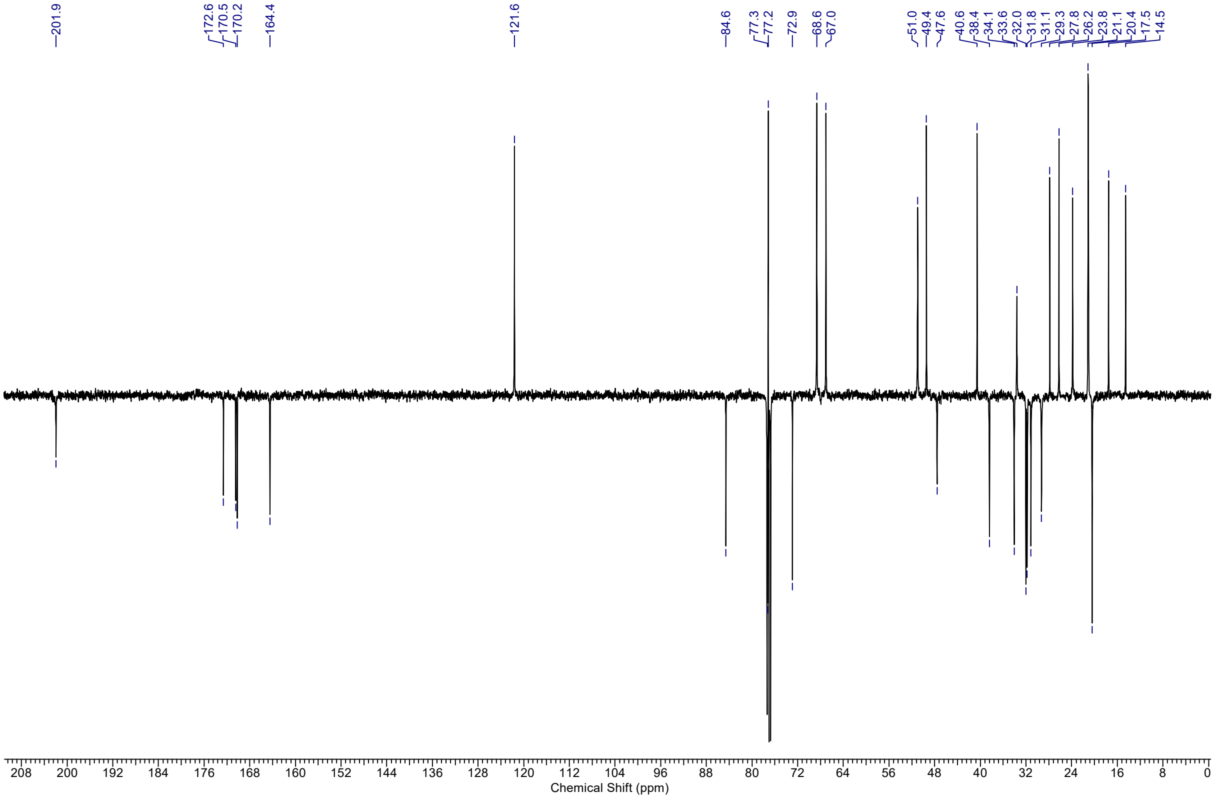

Compound 13: ^{1}H NMR (400 MHz, $CDCl_3$)

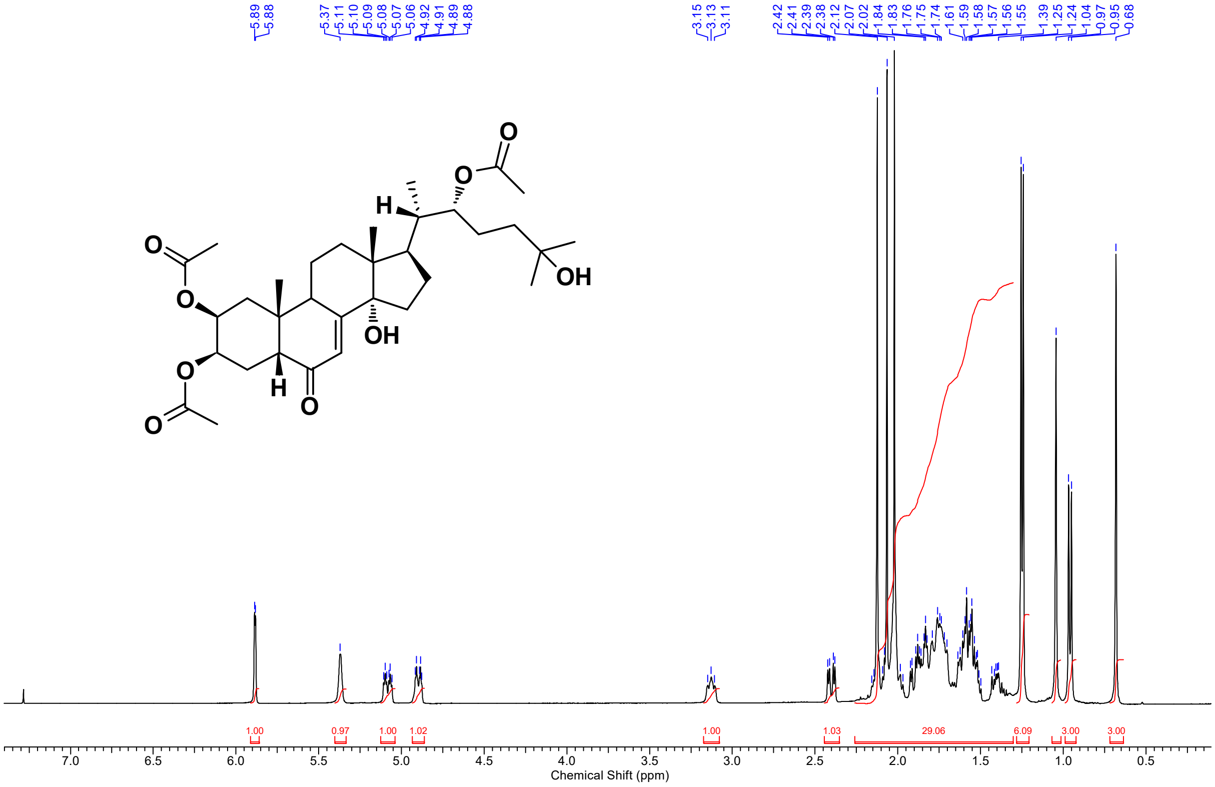

Compound 13: ^{13}C NMR (101 MHz, $CDCl_3$)

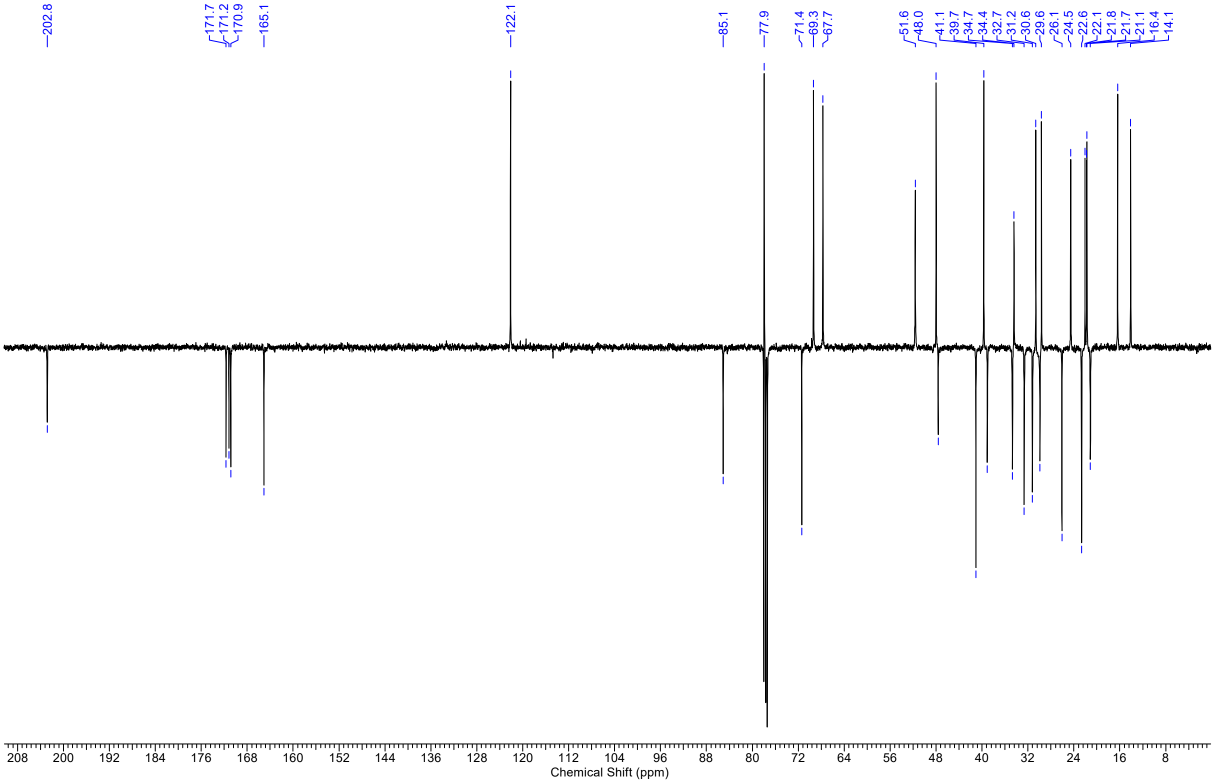

Compound 14: ^{1}H NMR (400 MHz, CDCl$_3$)

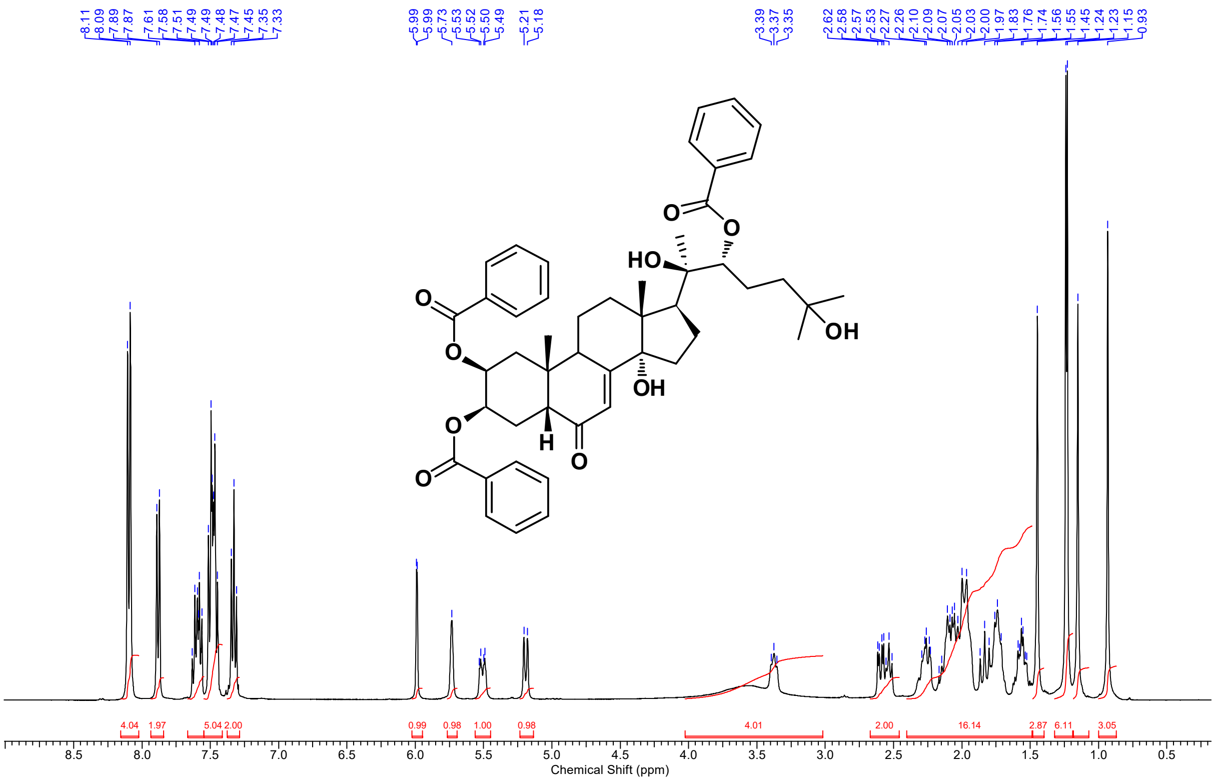

Compound 14: ^{13}C NMR (101 MHz, CDCl$_3$)

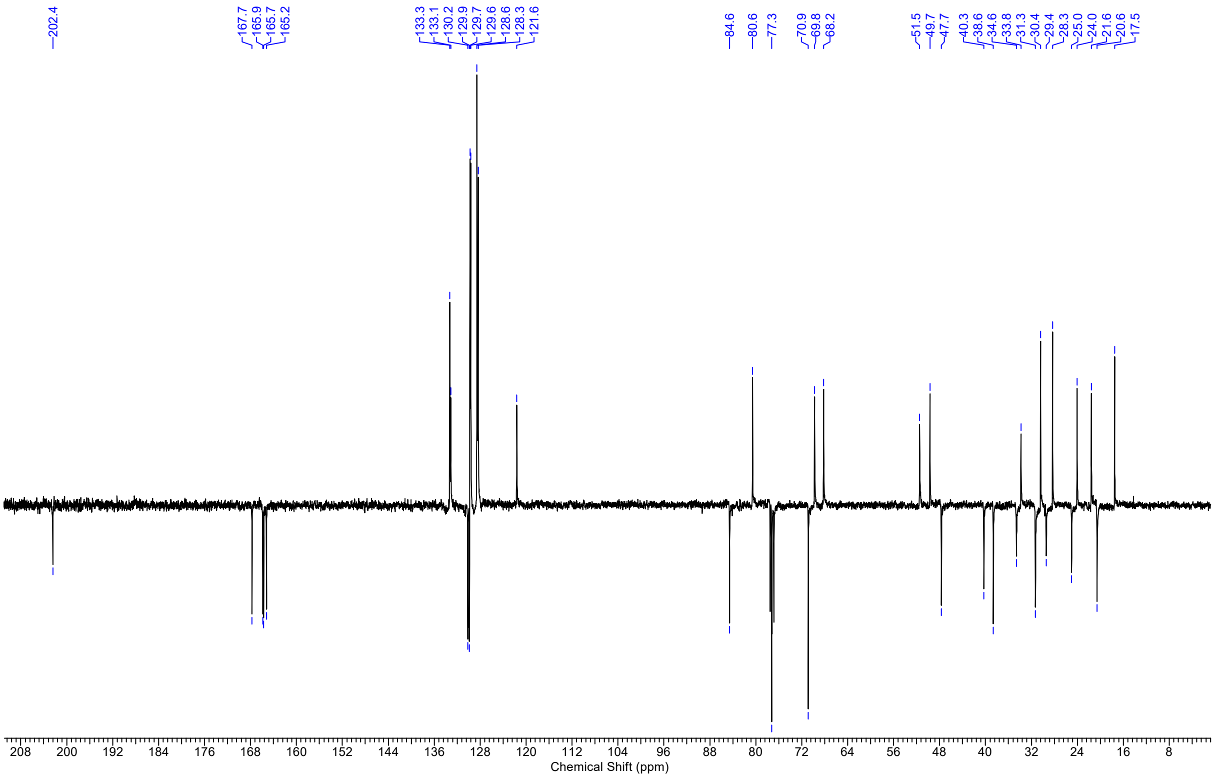

Compound 15: ^{1}H NMR (400 MHz, $CDCl_3$)

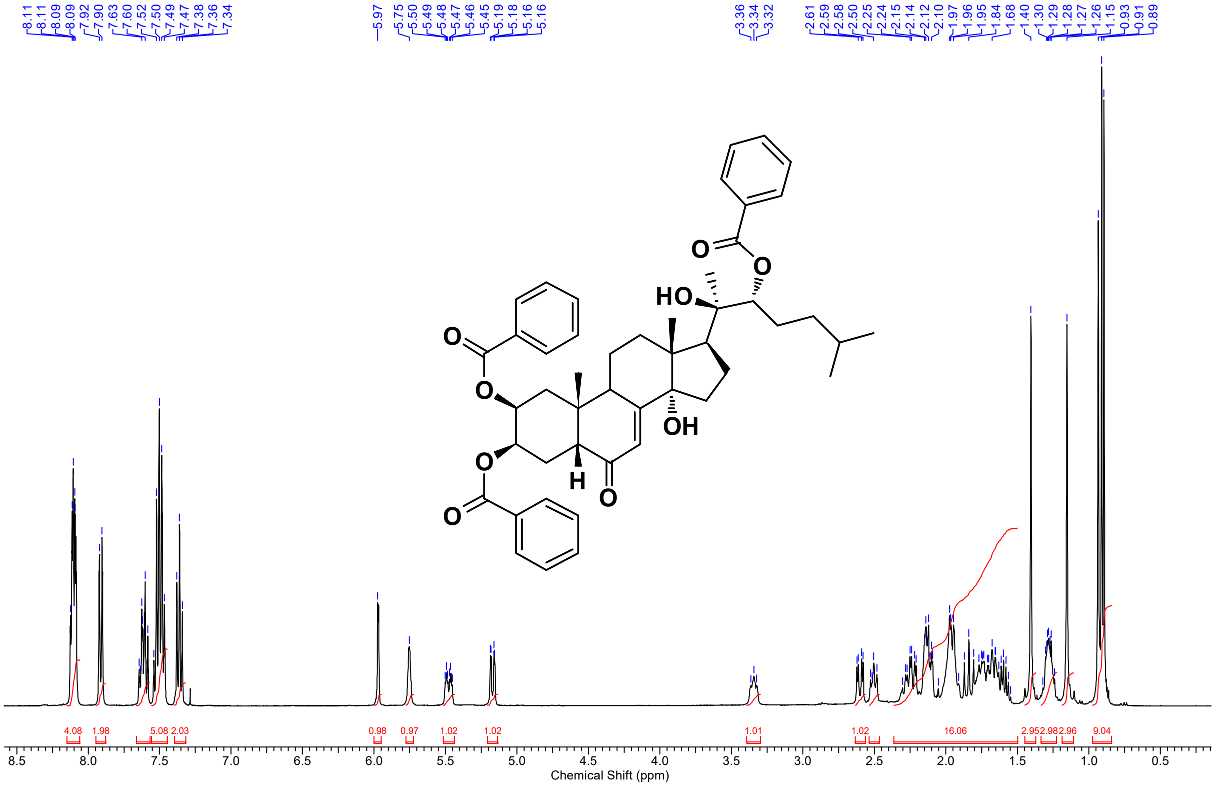

Compound 15: ^{13}C NMR (101 MHz, $CDCl_3$)

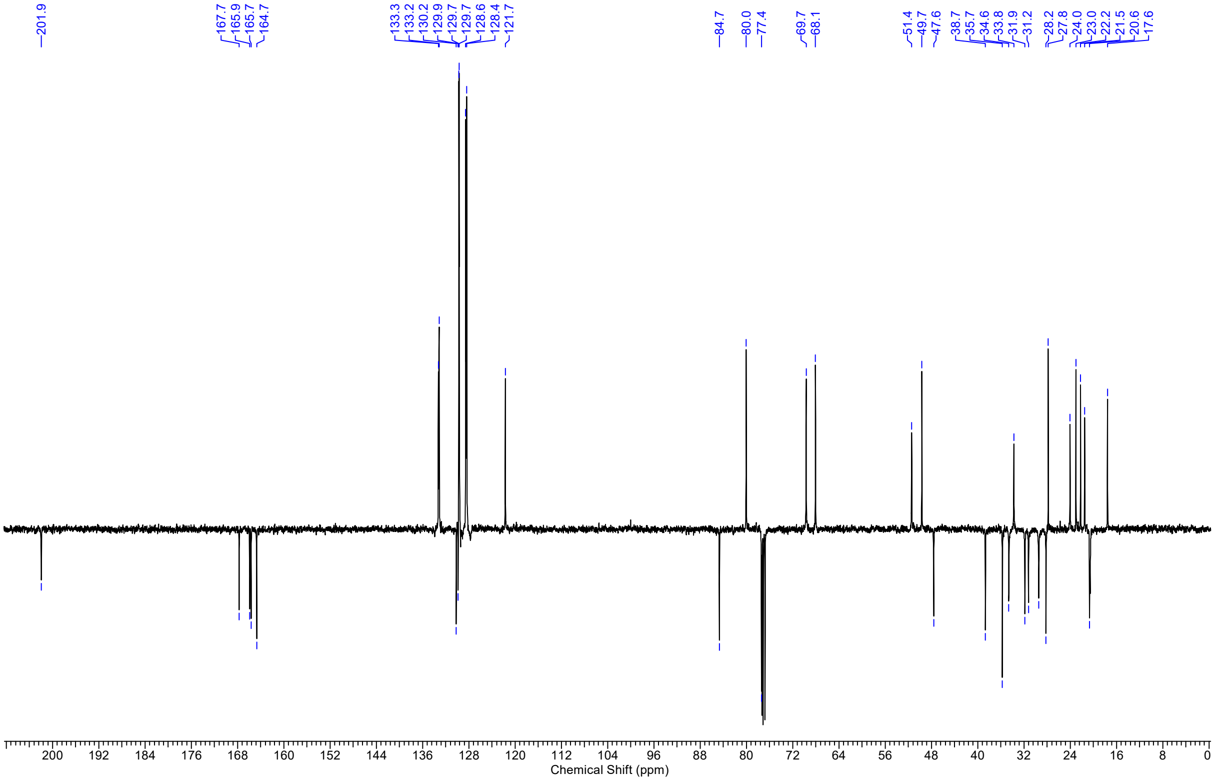

Compound 16: ^{1}H NMR (400 MHz, $CDCl_3$)

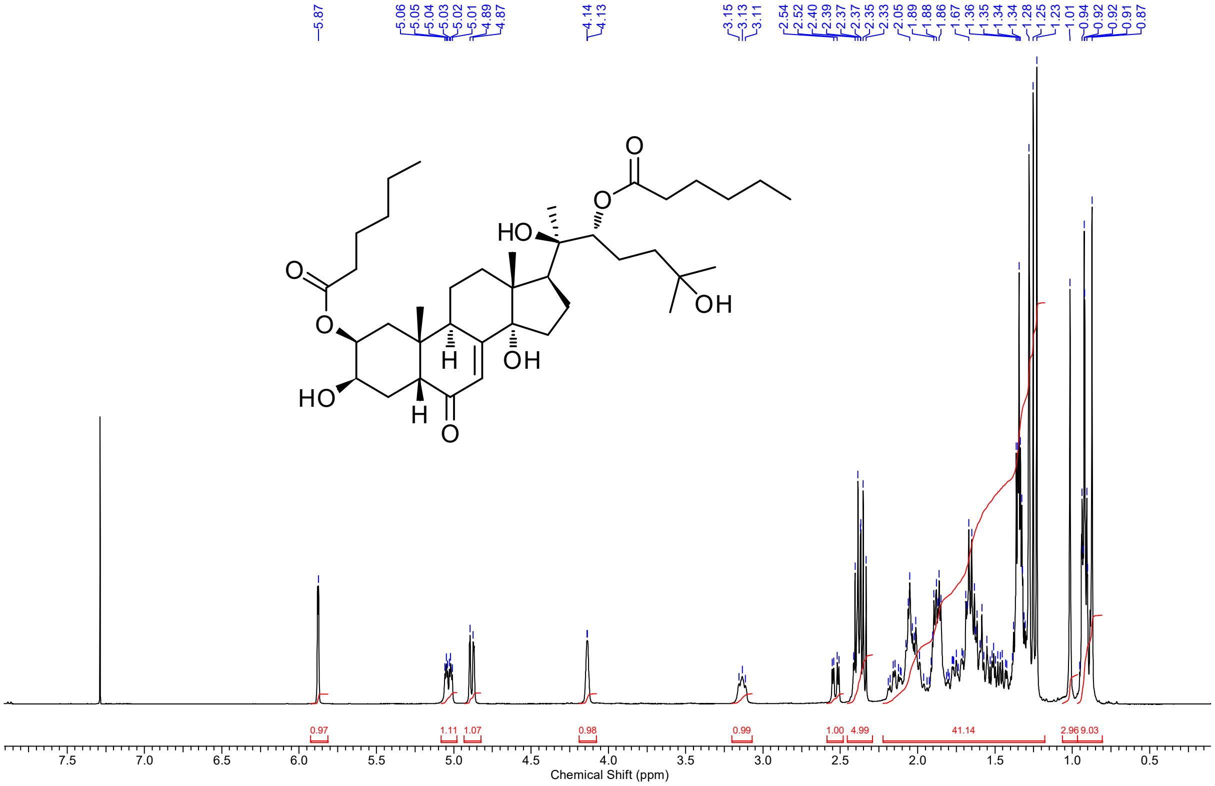

Compound 16: ^{13}C NMR (101 MHz, $CDCl_3$)

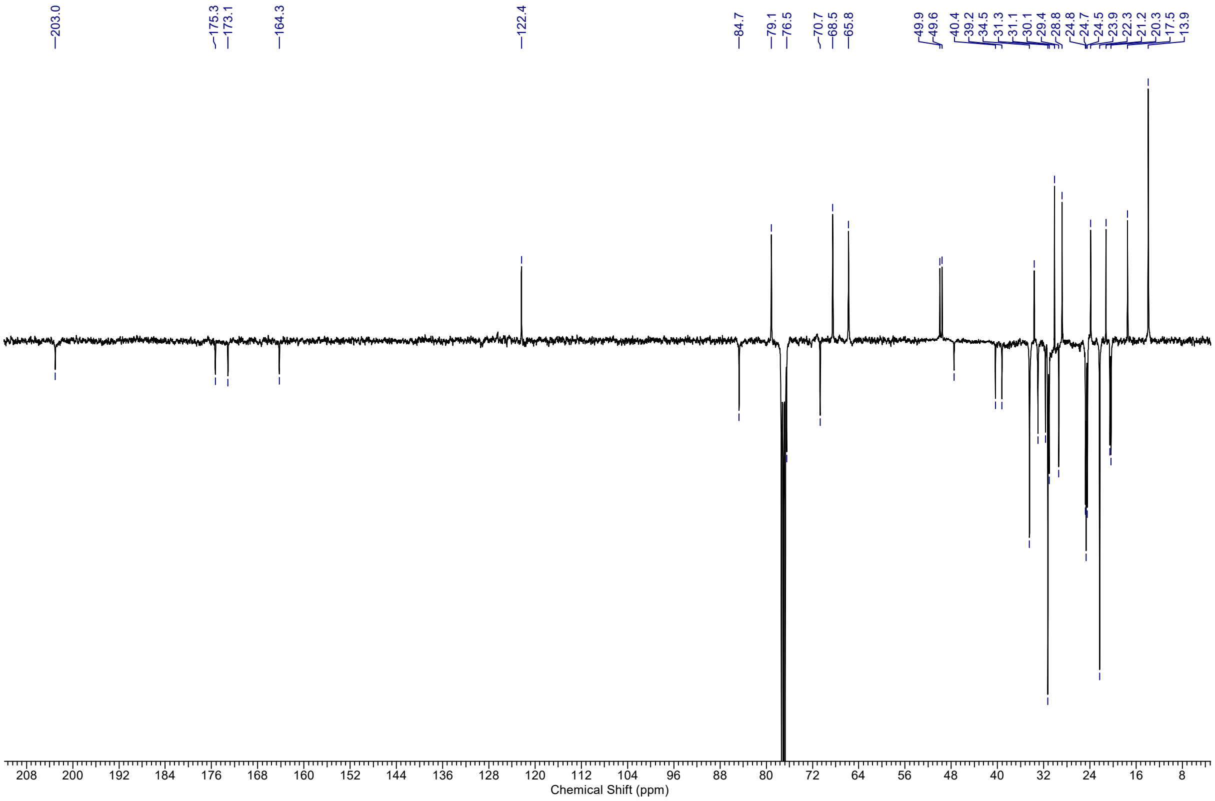

Compound 17: ^{1}H NMR (400 MHz, $CDCl_3$)

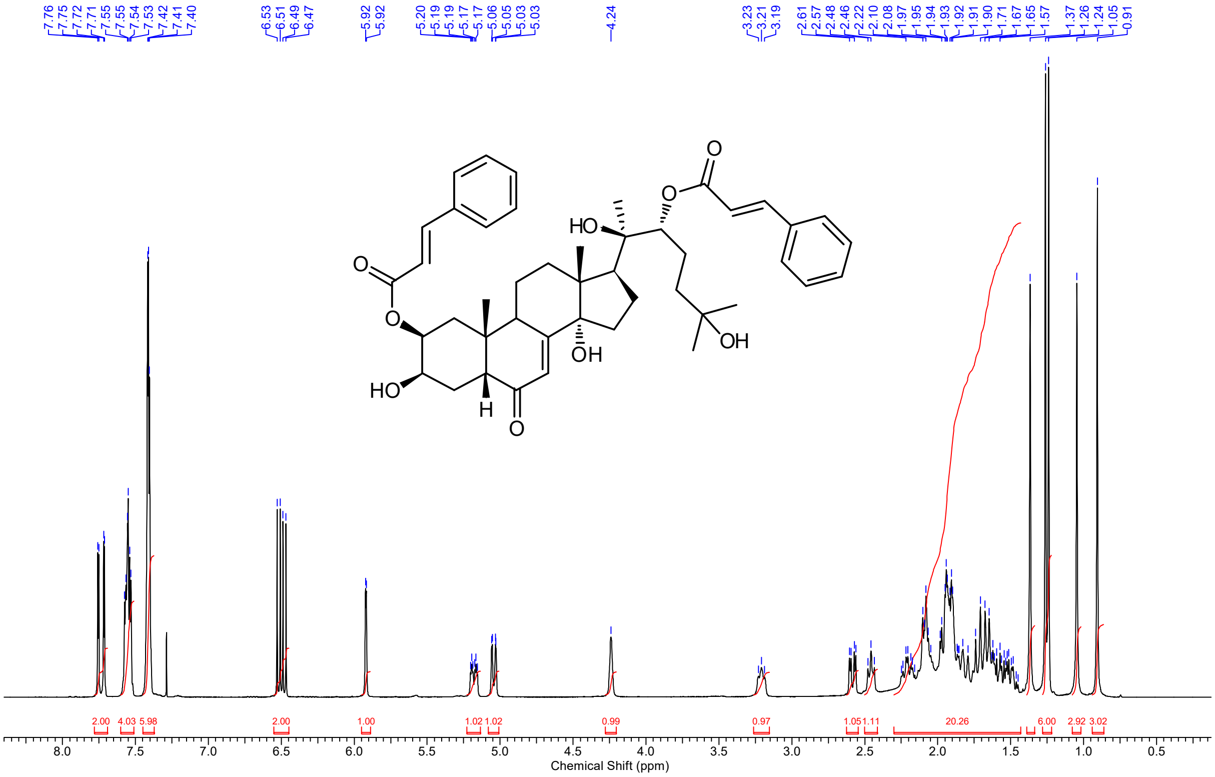

Compound 17: ^{13}C NMR (101 MHz, $CDCl_3$)

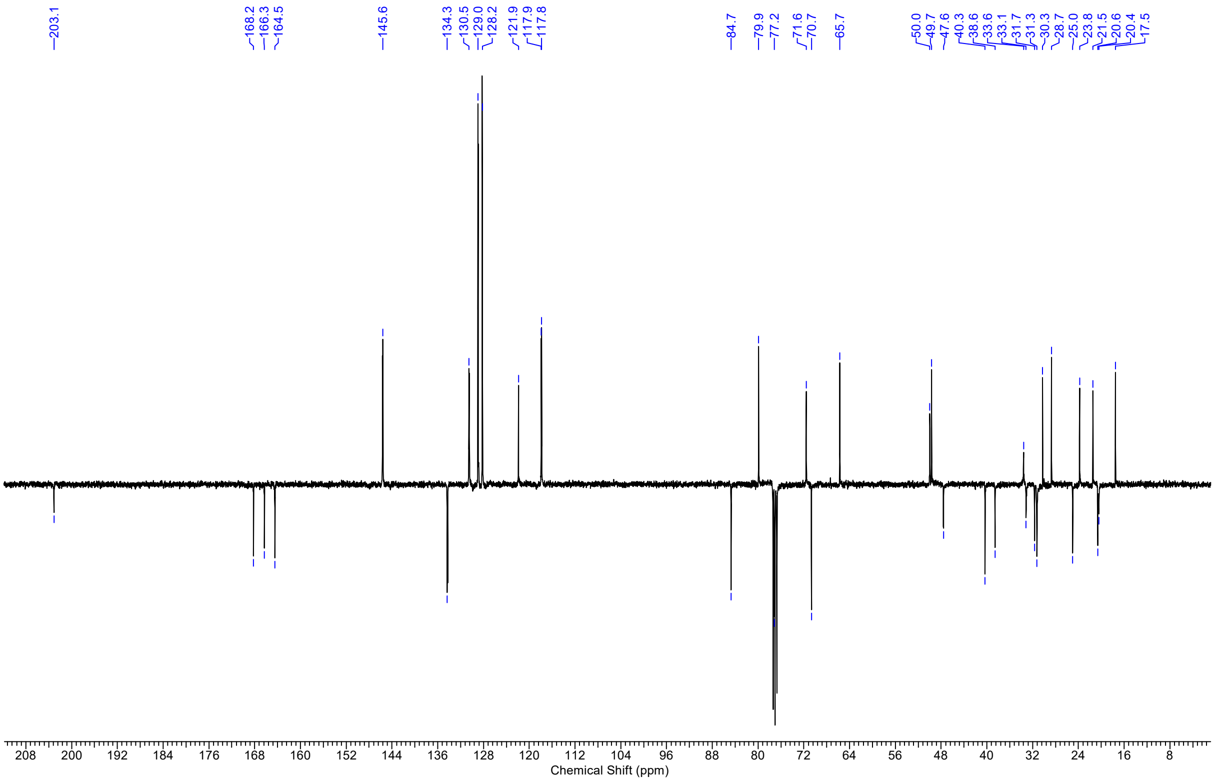

Compound 18: ^{1}H NMR (400 MHz, $CDCl_3$)

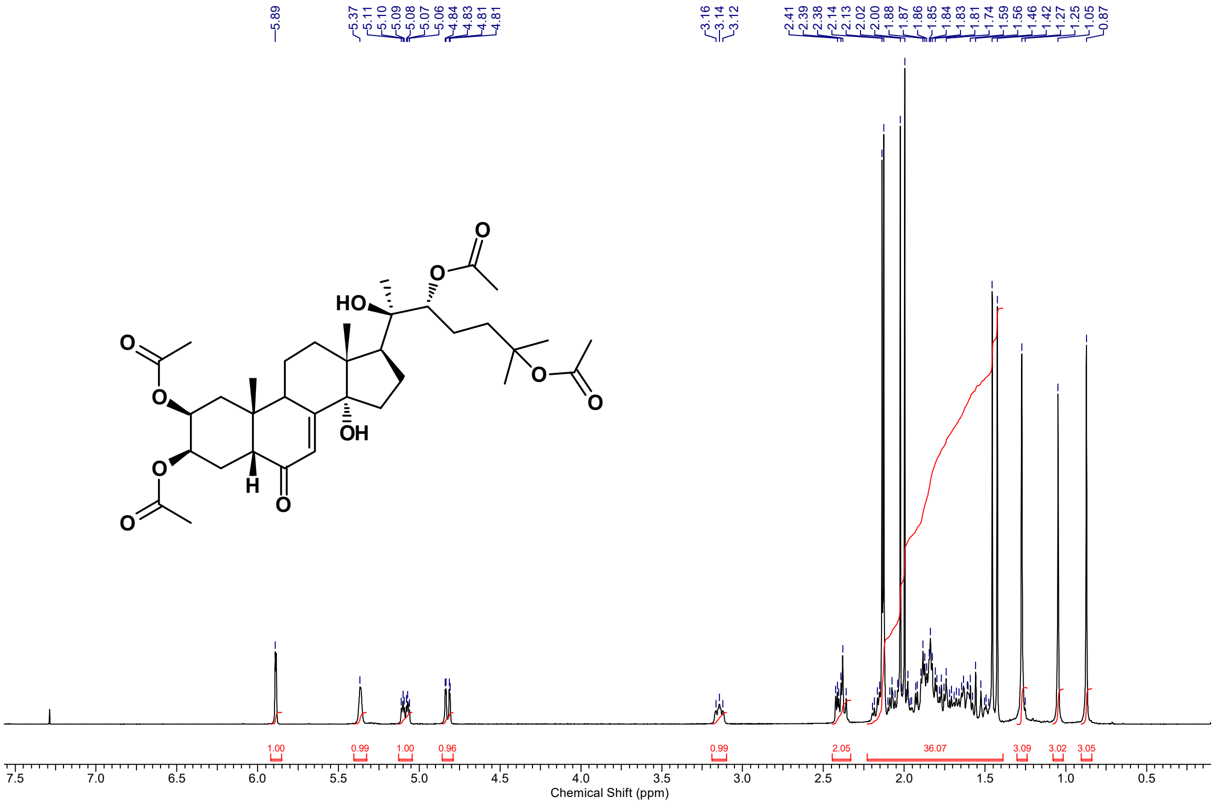

Compound 18: ^{13}C NMR (75 MHz, $CDCl_3$)

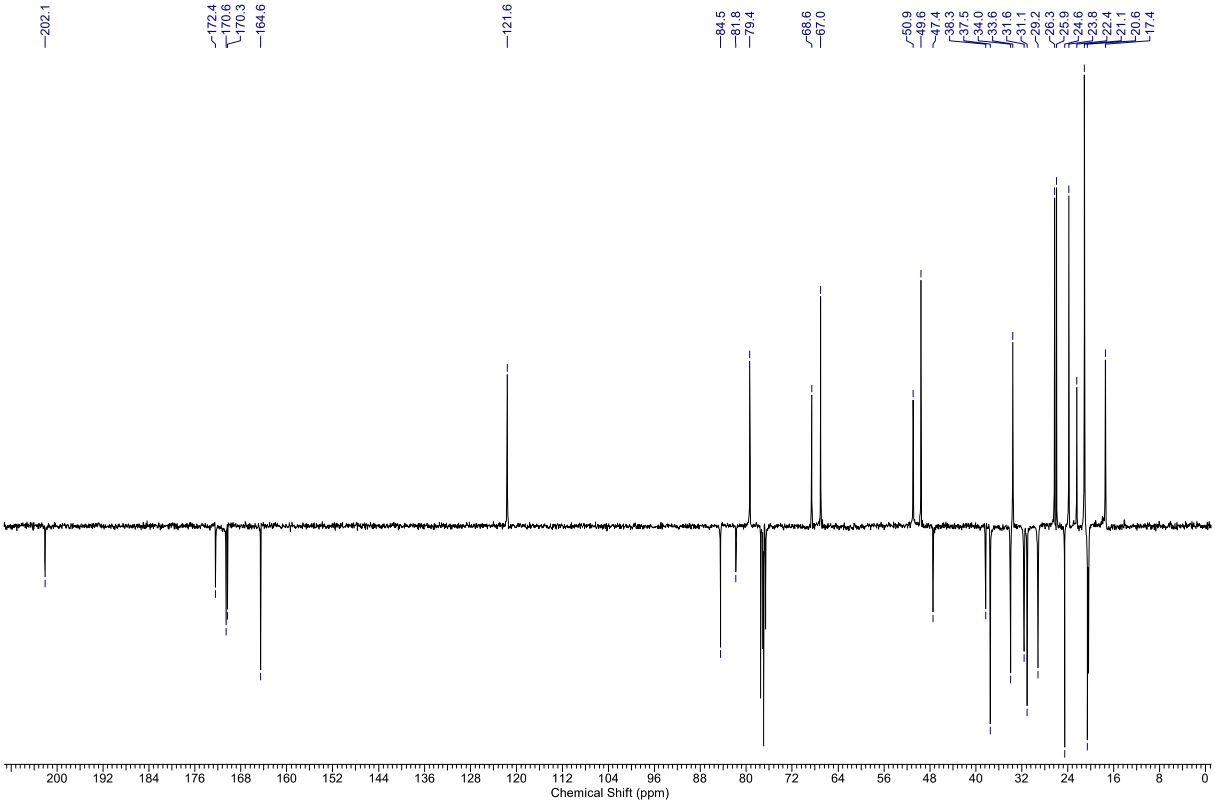

Compound 19: ^{1}H NMR (400 MHz, $CDCl_3$)

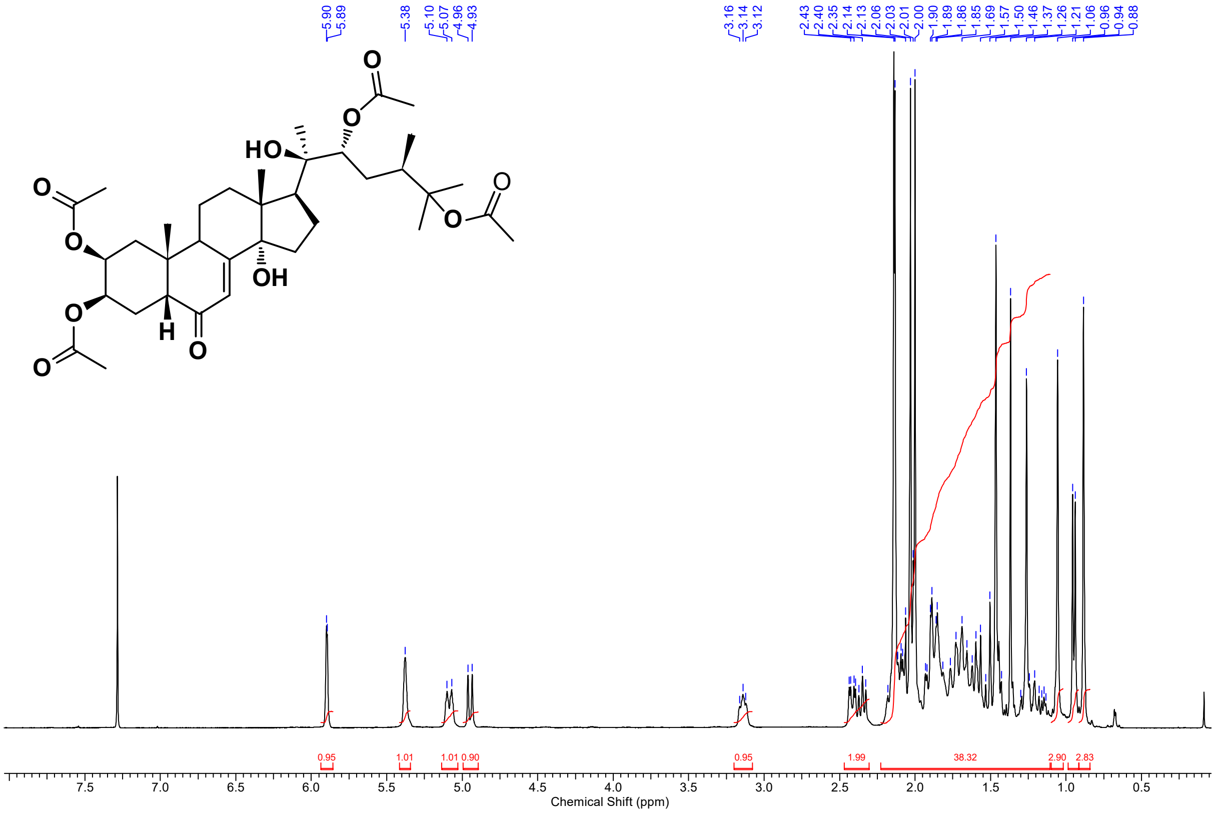

Compound 19: ^{13}C NMR (75 MHz, $CDCl_3$)

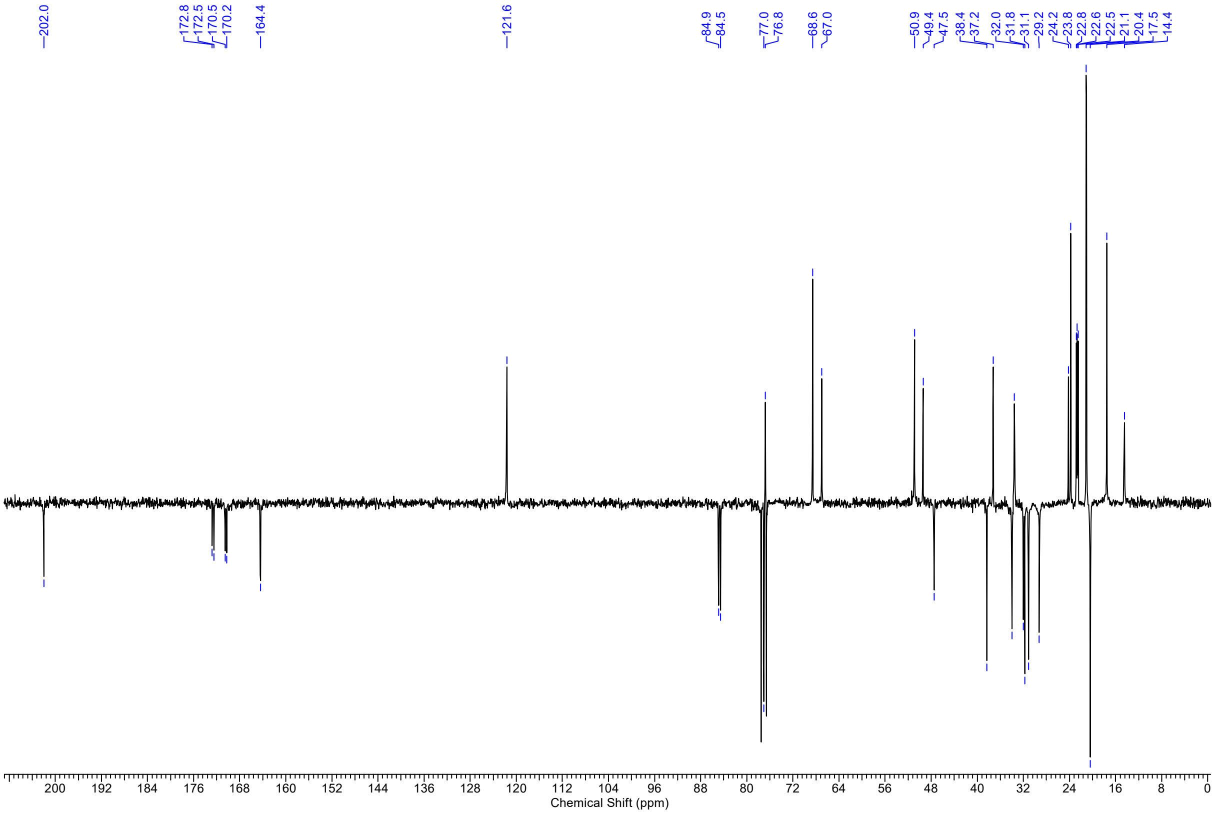

Compound 20: ^{1}H NMR (400 MHz, $CDCl_3$)

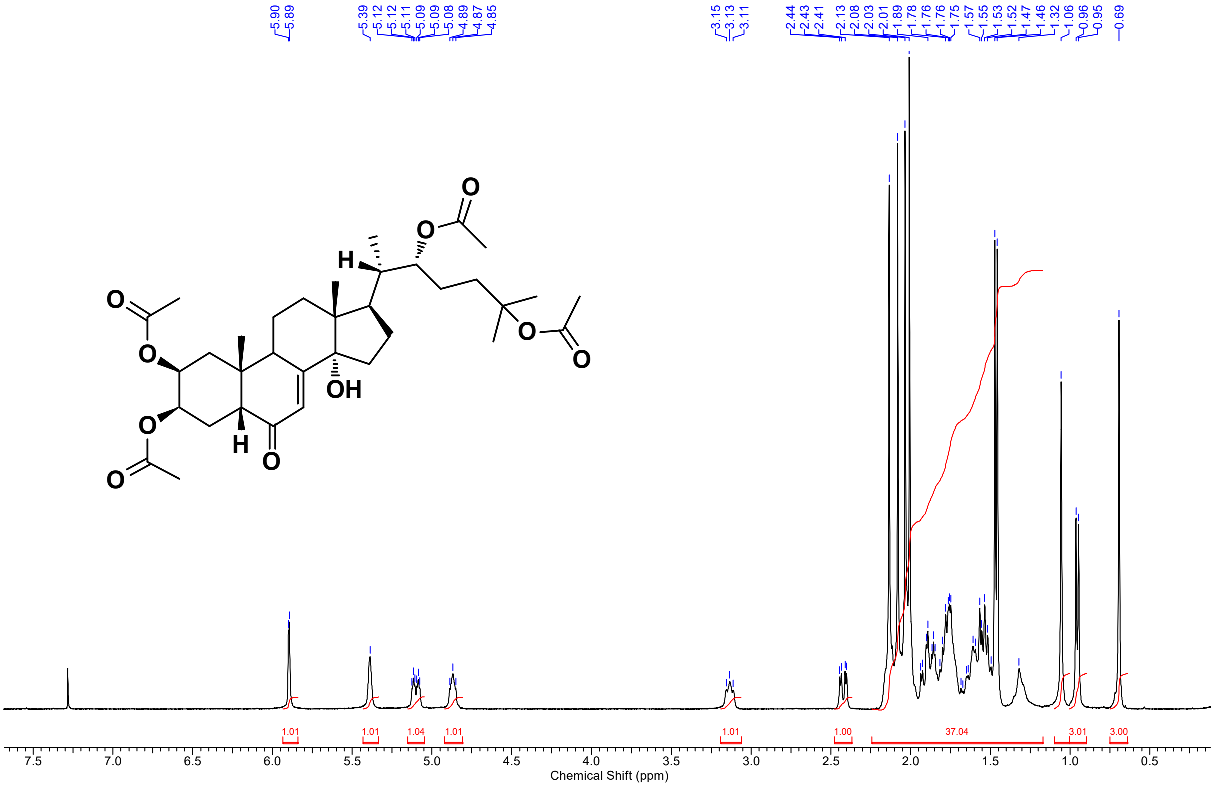

Compound 20: ^{13}C NMR (101 MHz, $CDCl_3$)

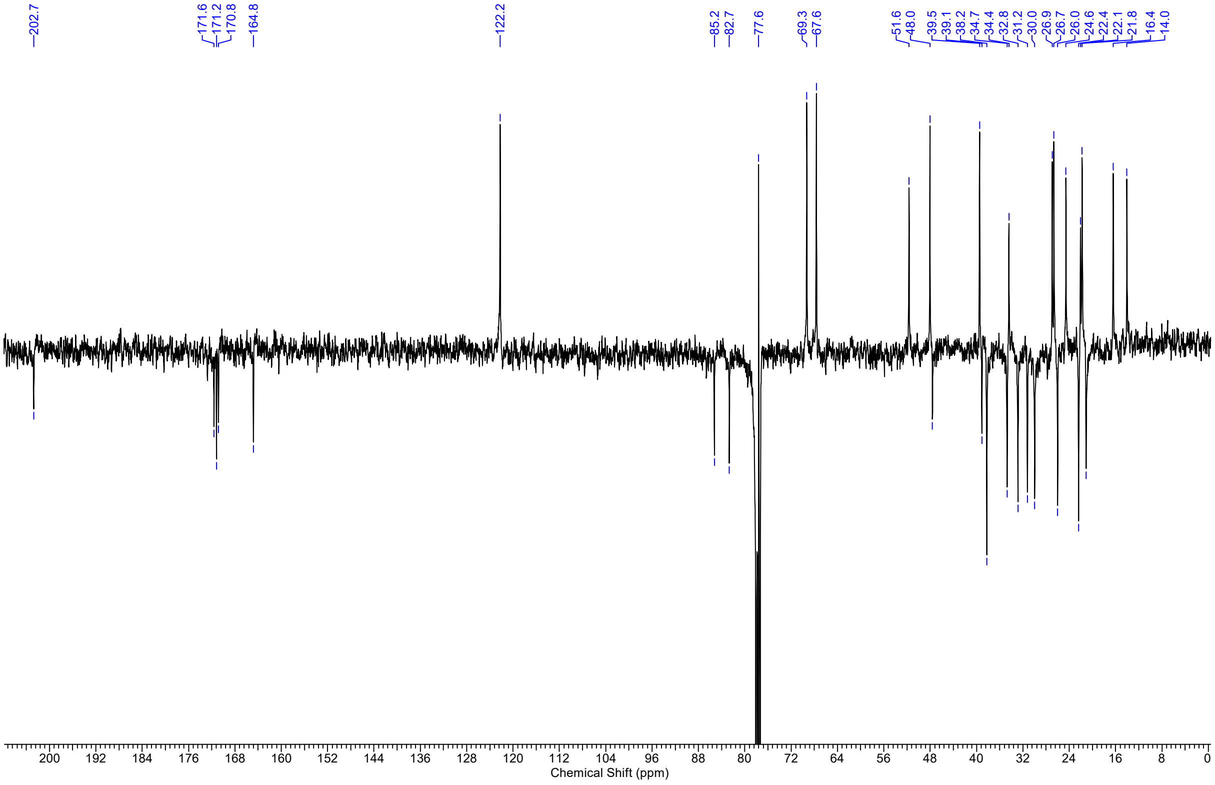

Compound 21: ^{1}H NMR (400 MHz, $CDCl_3$)

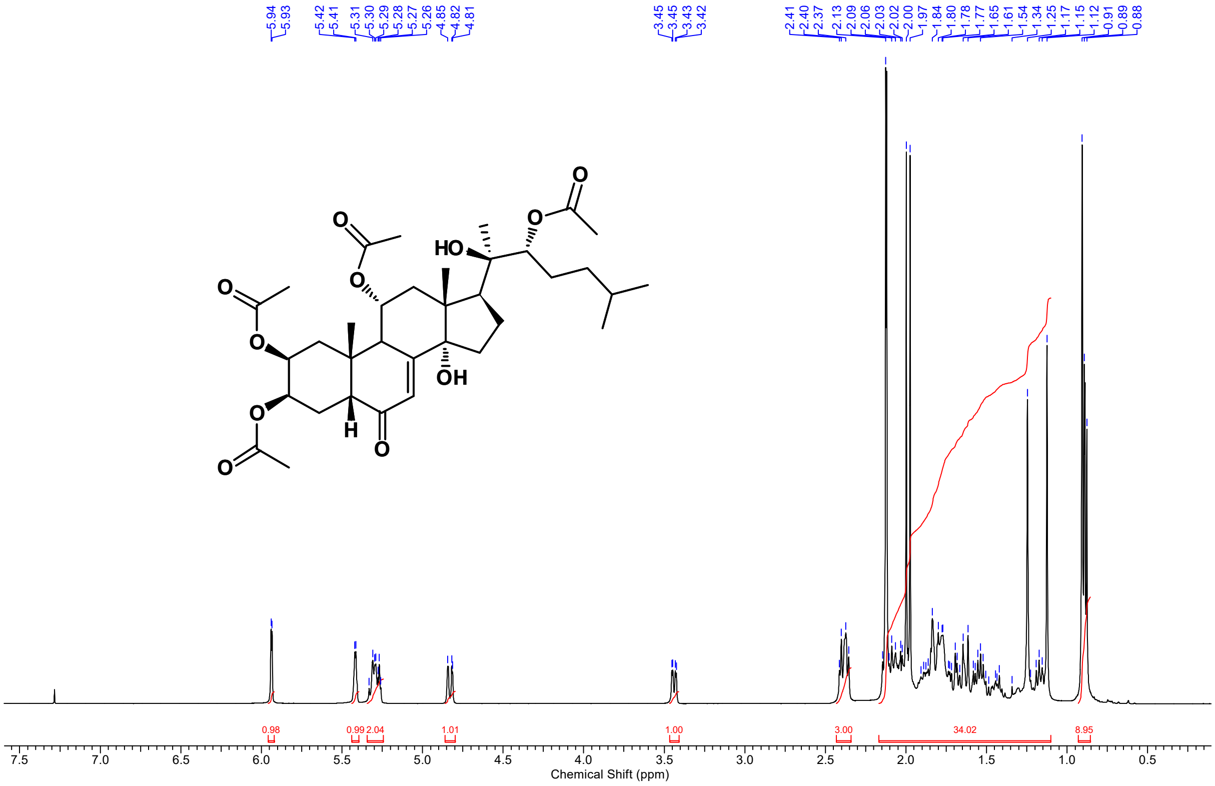

Compound 21: ^{13}C NMR (101 MHz, CDCl$_3$)

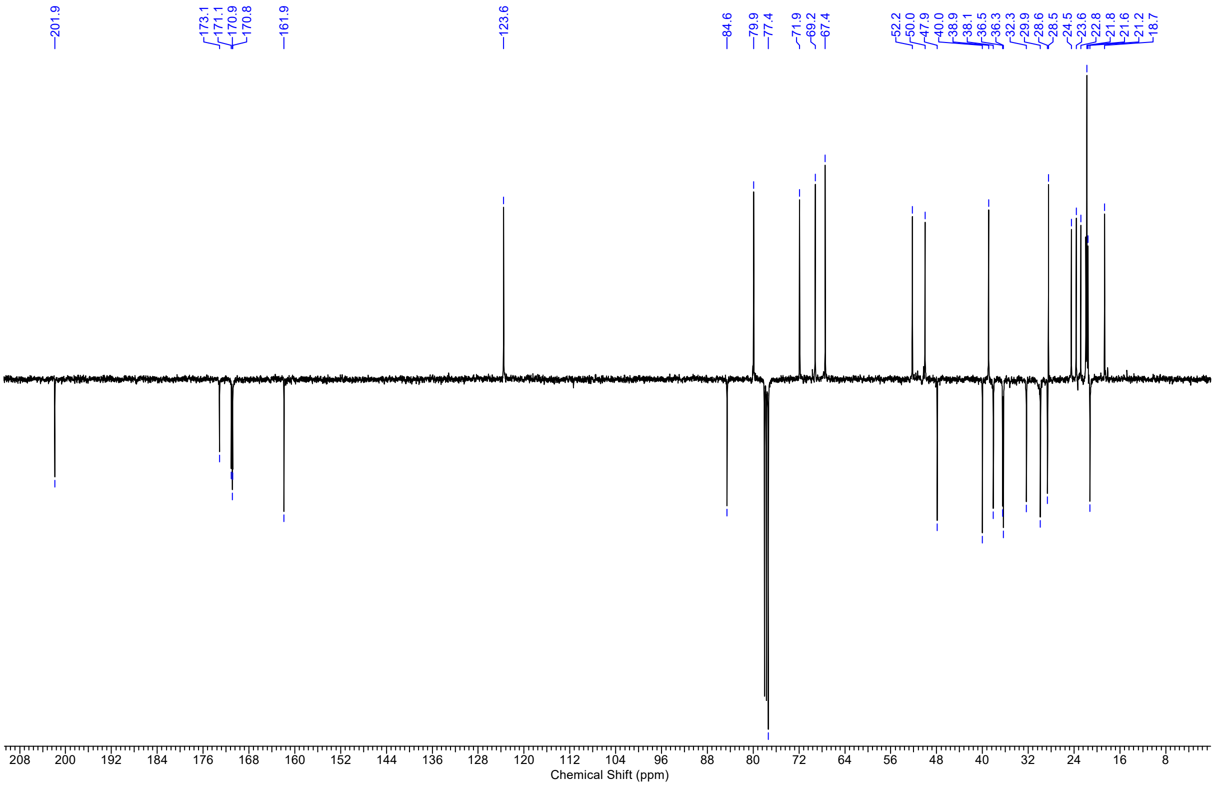

Compound 22: ^{1}H NMR (400 MHz, CDCl$_3$)

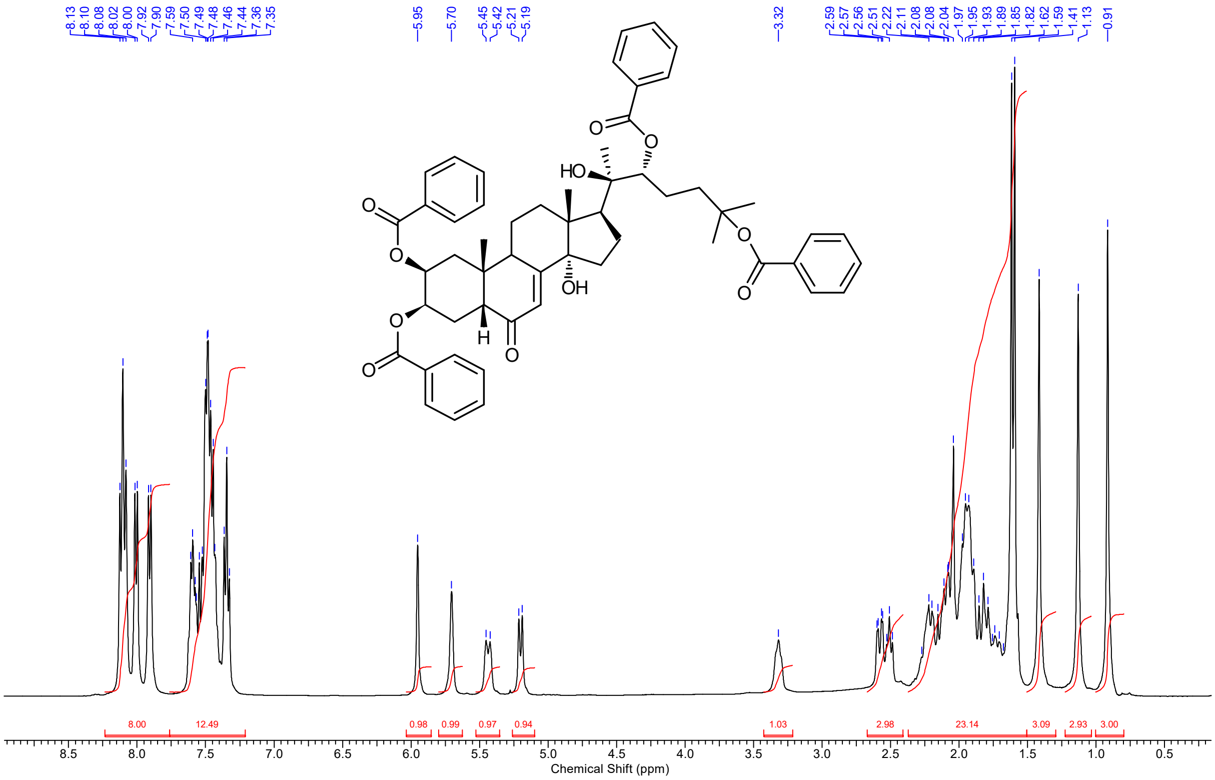

Compound 22: ^{13}C NMR (101 MHz, $CDCl_3$)

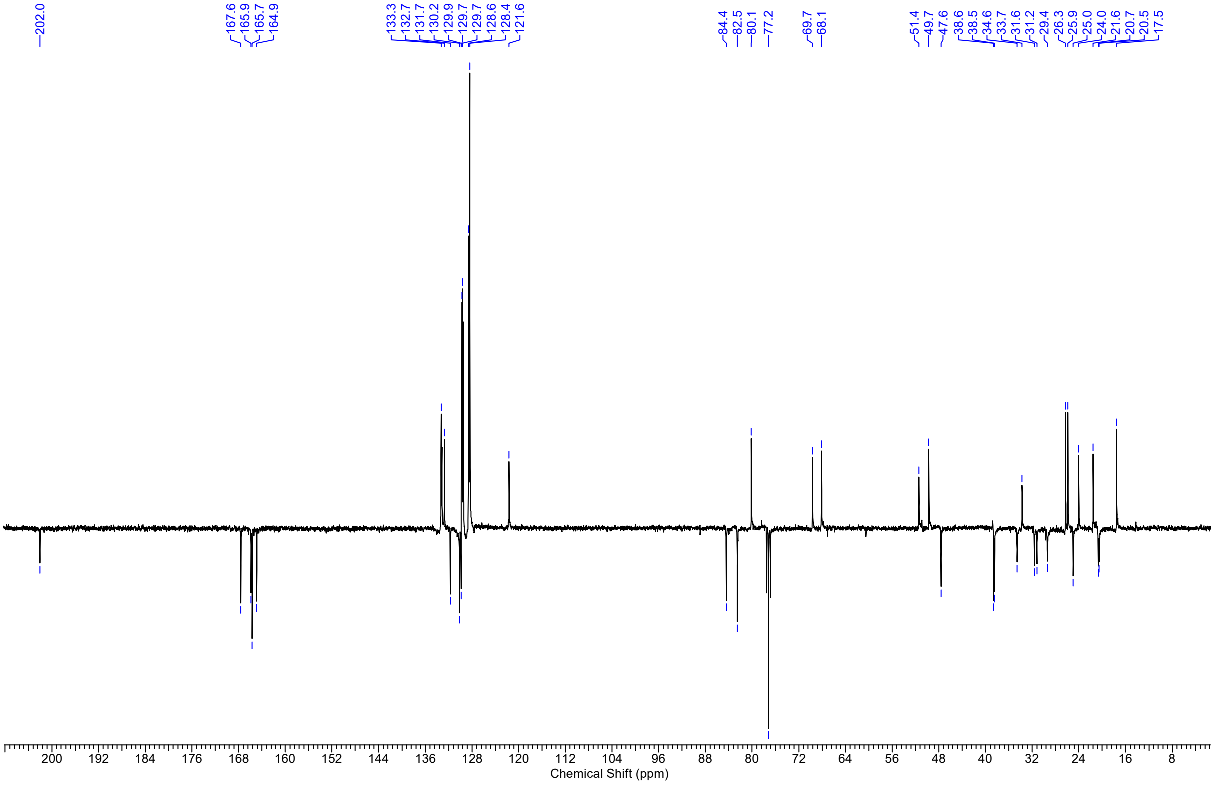

Compound 23: ^{1}H NMR (400 MHz, $CDCl_3$)

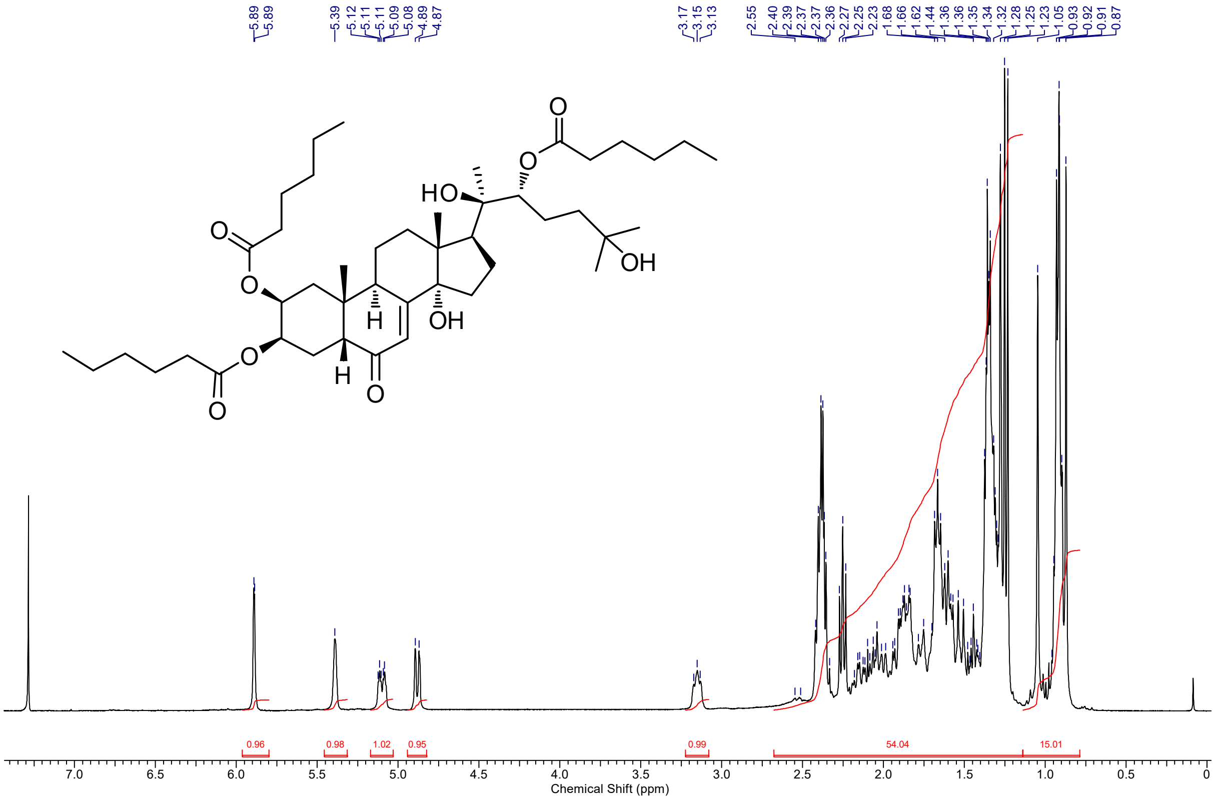

Compound 23: ^{13}C NMR (101 MHz, CDCl$_3$)

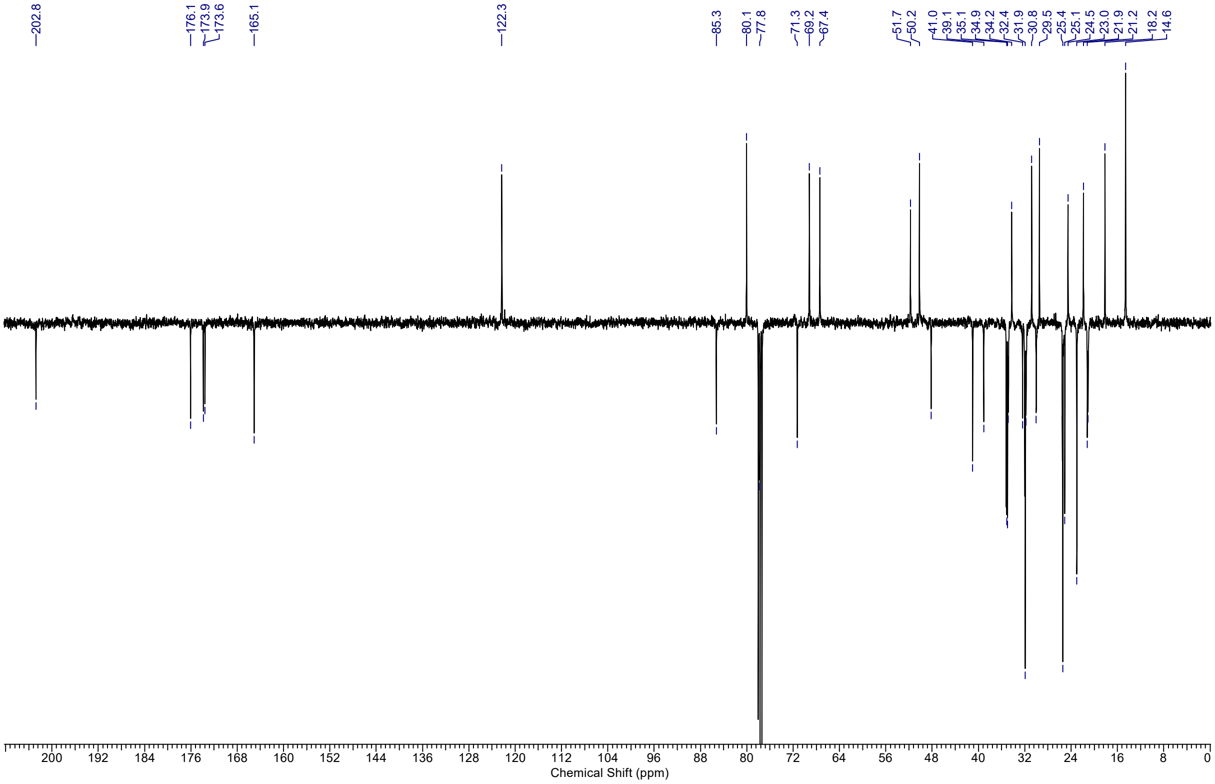

Compound 24: ^{1}H NMR (400 MHz, CDCl$_3$)

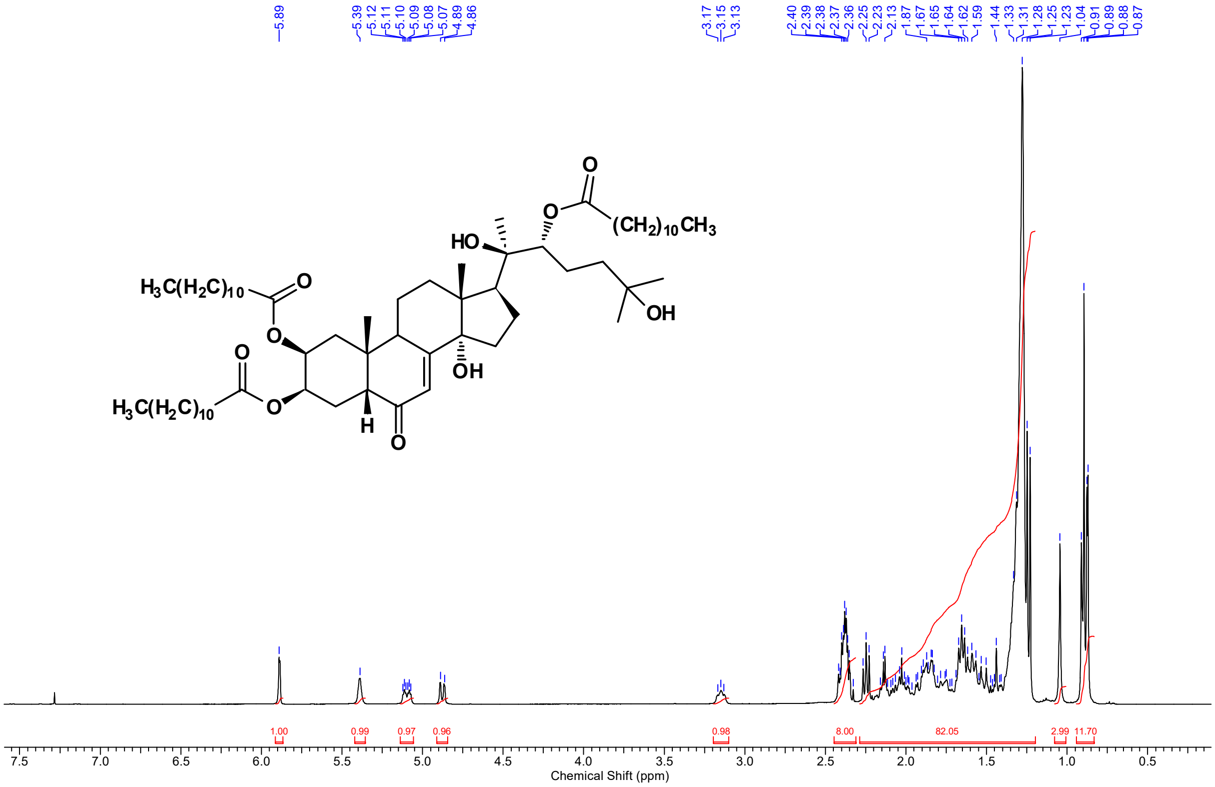

Compound 24: ^{13}C NMR (101 MHz, $CDCl_3$)

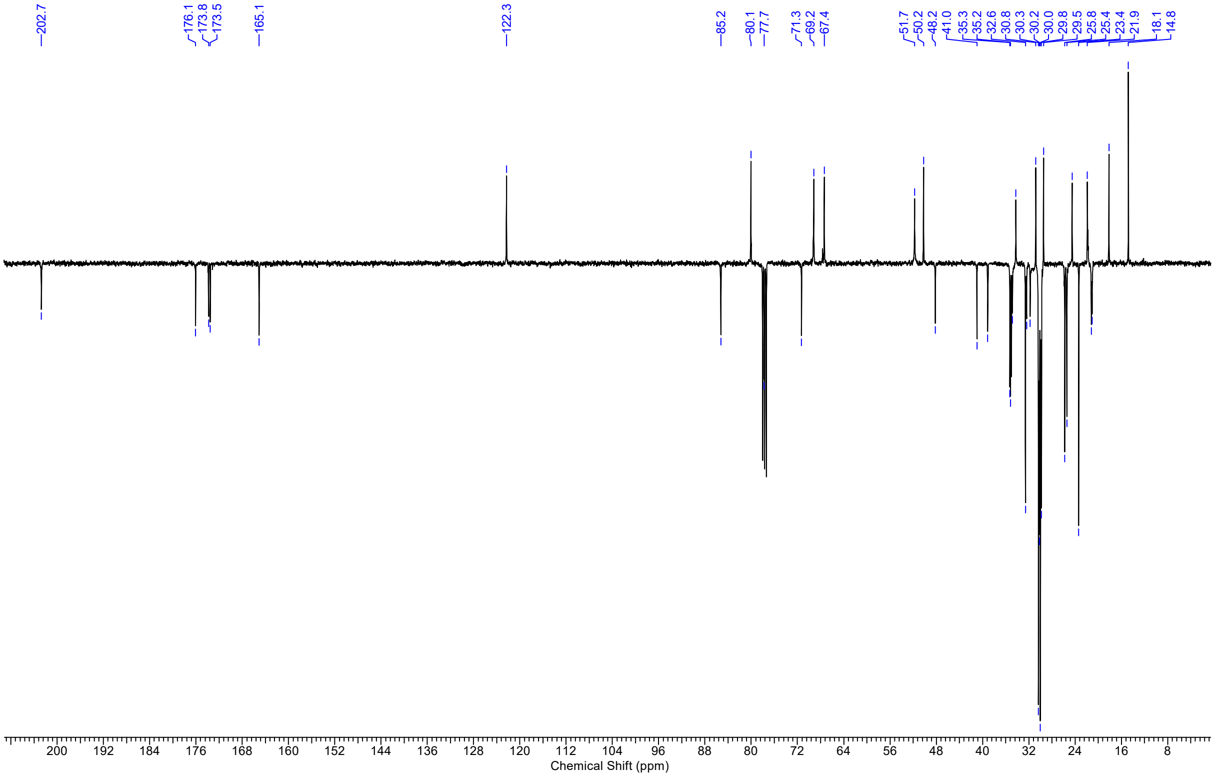

Compound 25: ^{1}H NMR (400 MHz, $CDCl_3$)

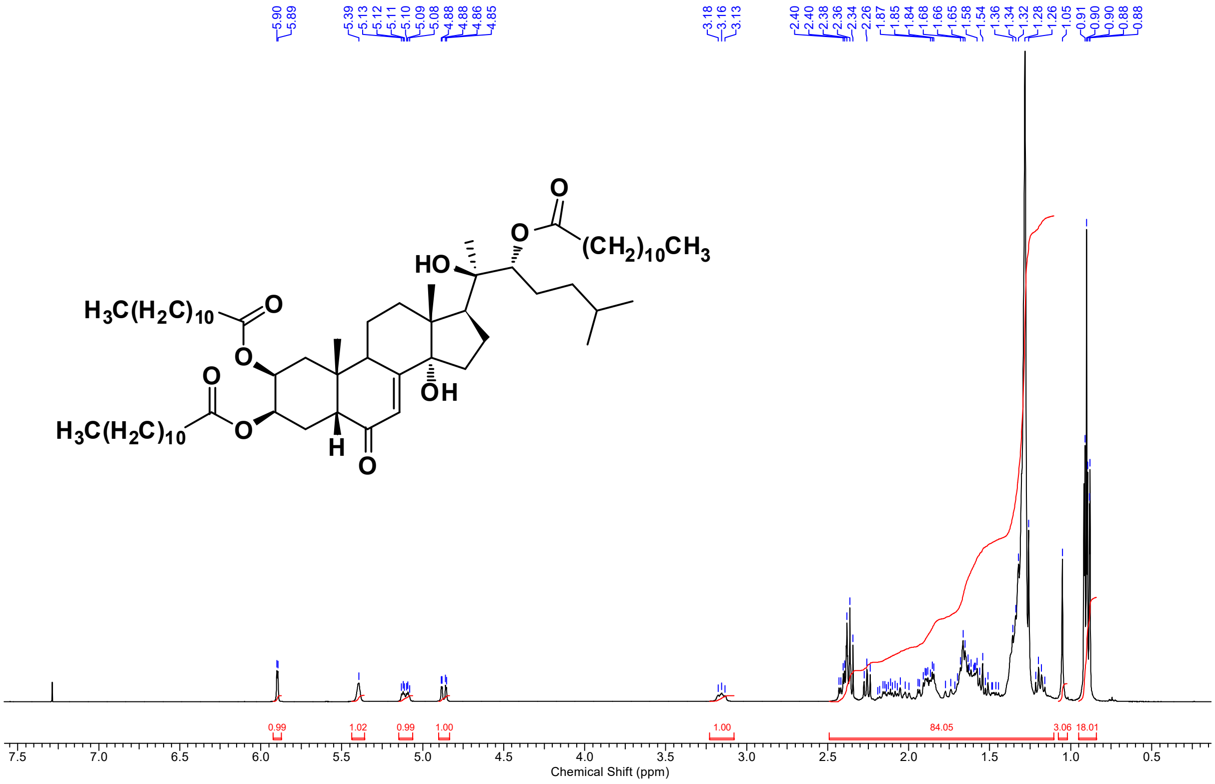

Compound 25: ^{13}C NMR (101 MHz, $CDCl_3$)

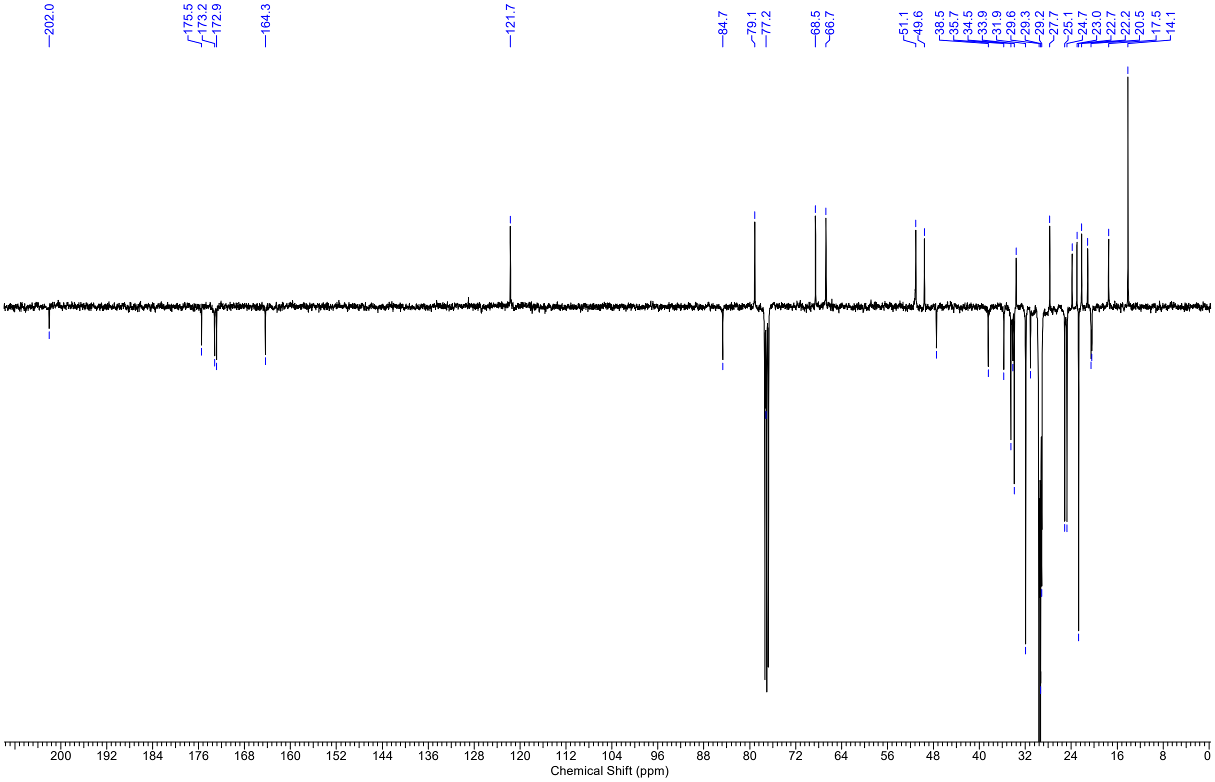

Compound 26: ^{1}H NMR (400 MHz, $CDCl_3$)

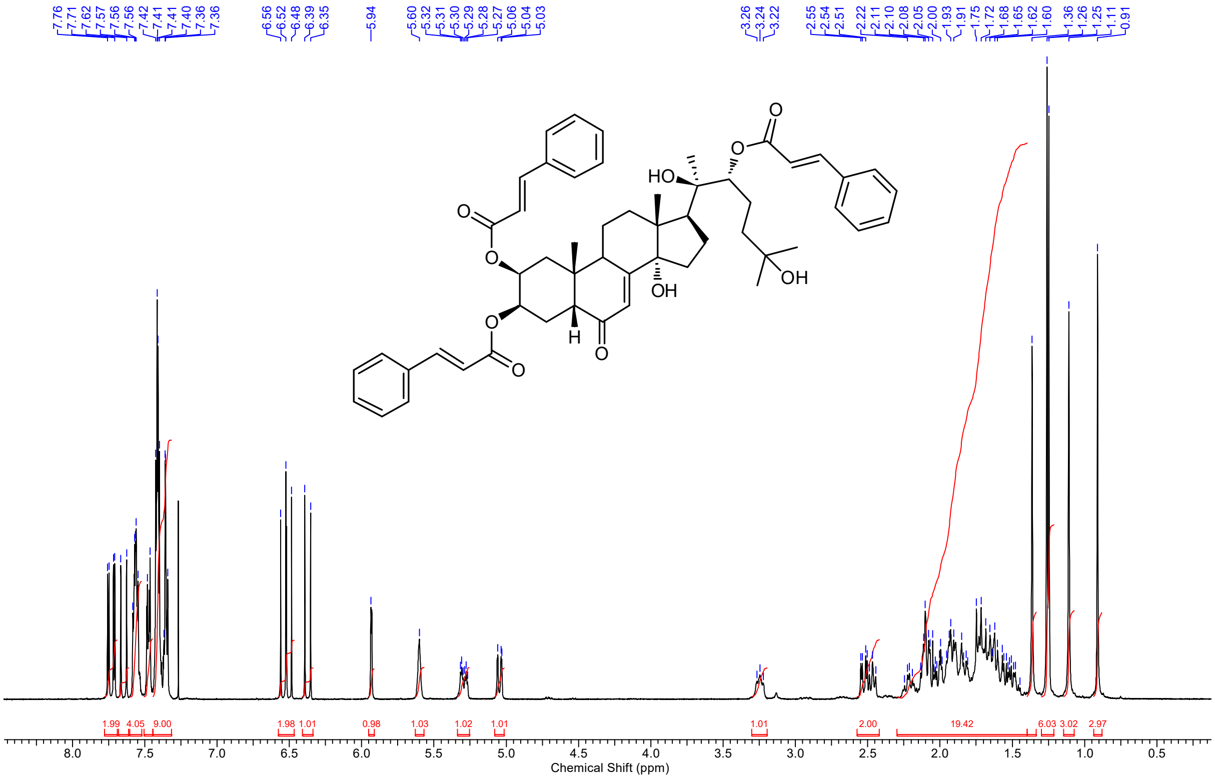

Compound 26: ^{13}C NMR (101 MHz, $CDCl_3$)

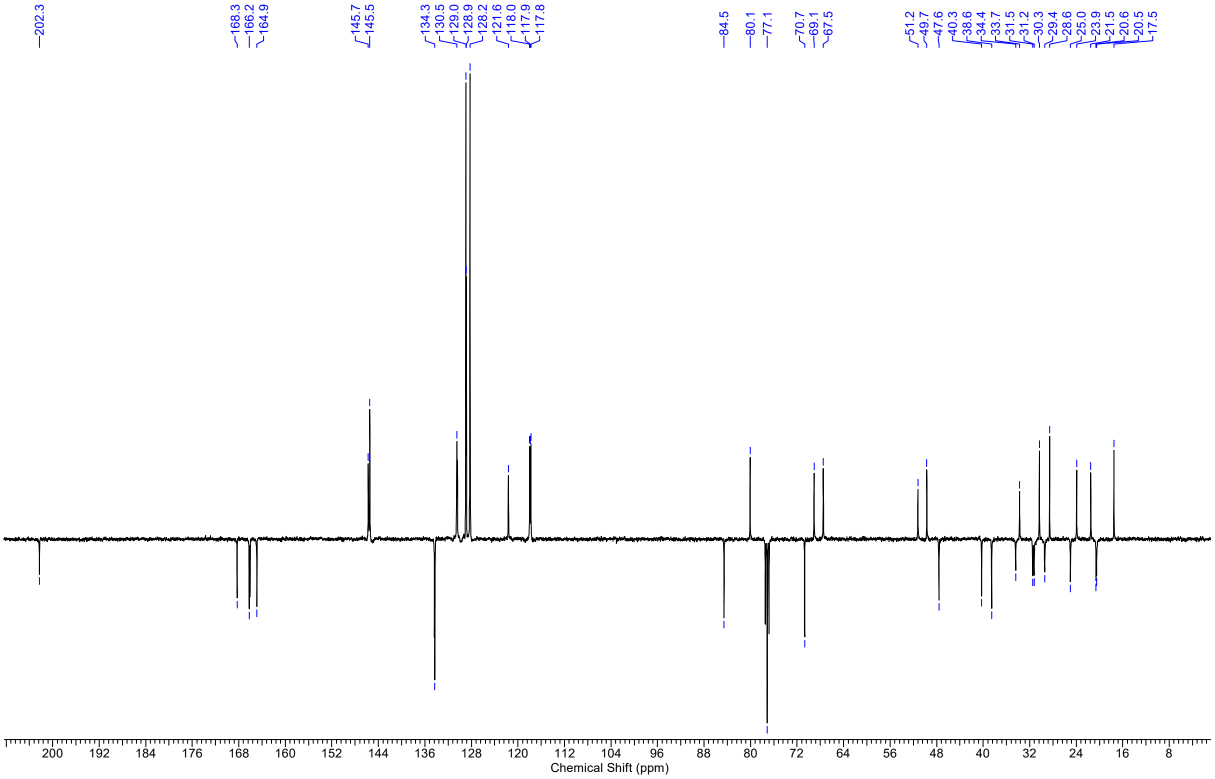

Compound 27: ^{1}H NMR (400 MHz, $CDCl_3$)

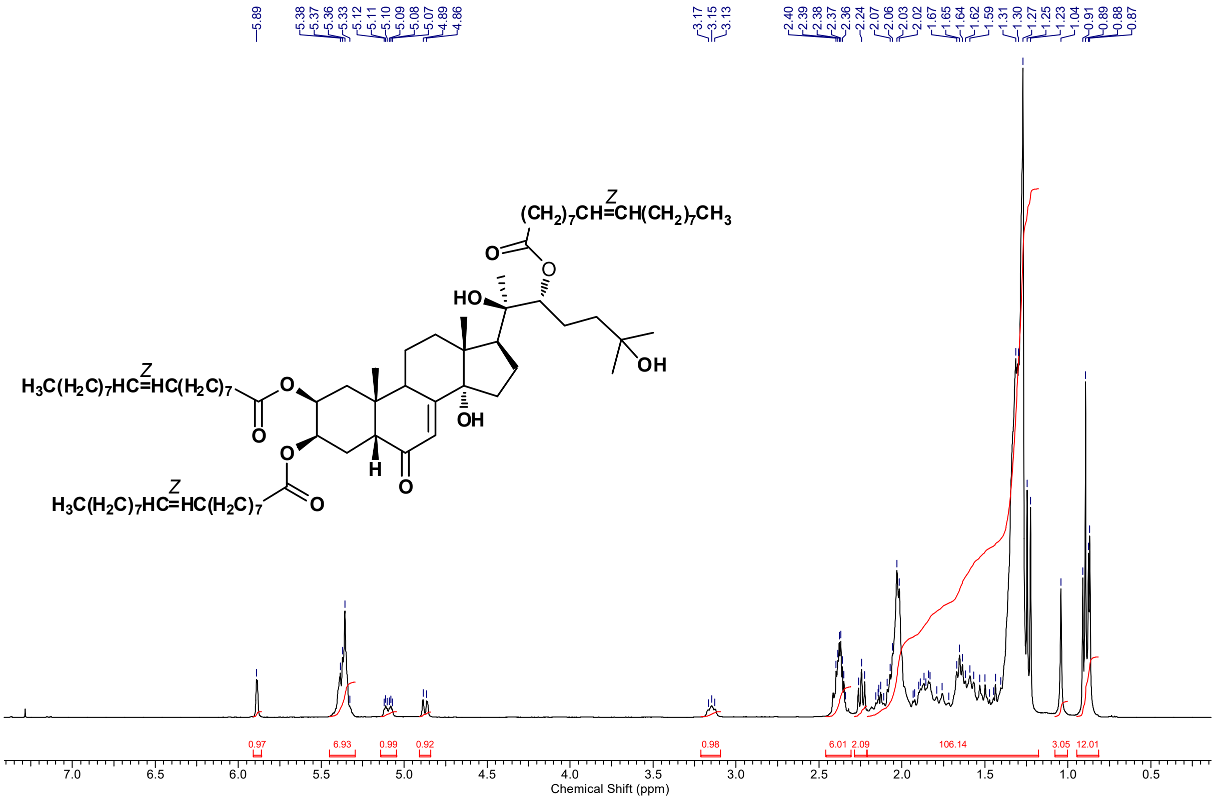

Compound 27: ^{13}C NMR (101 MHz, $CDCl_3$)

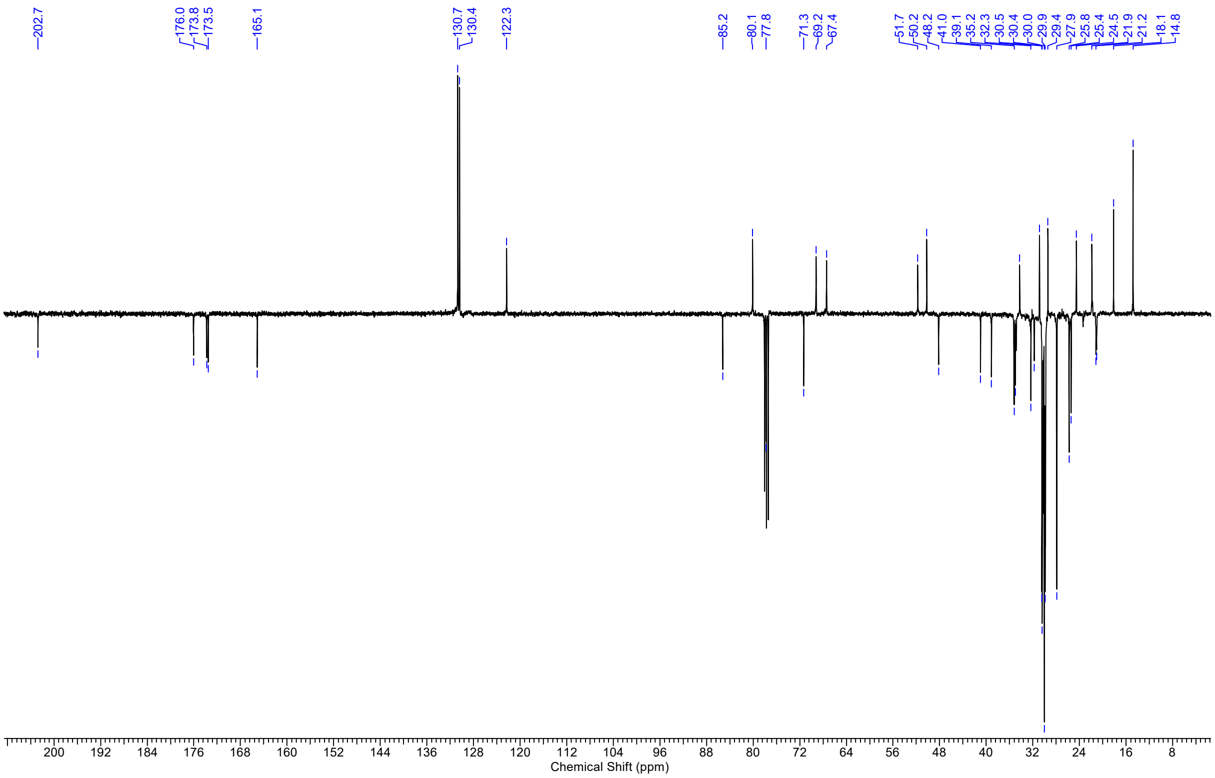

Compound 28: ^{1}H NMR (400 MHz, $CDCl_3$)

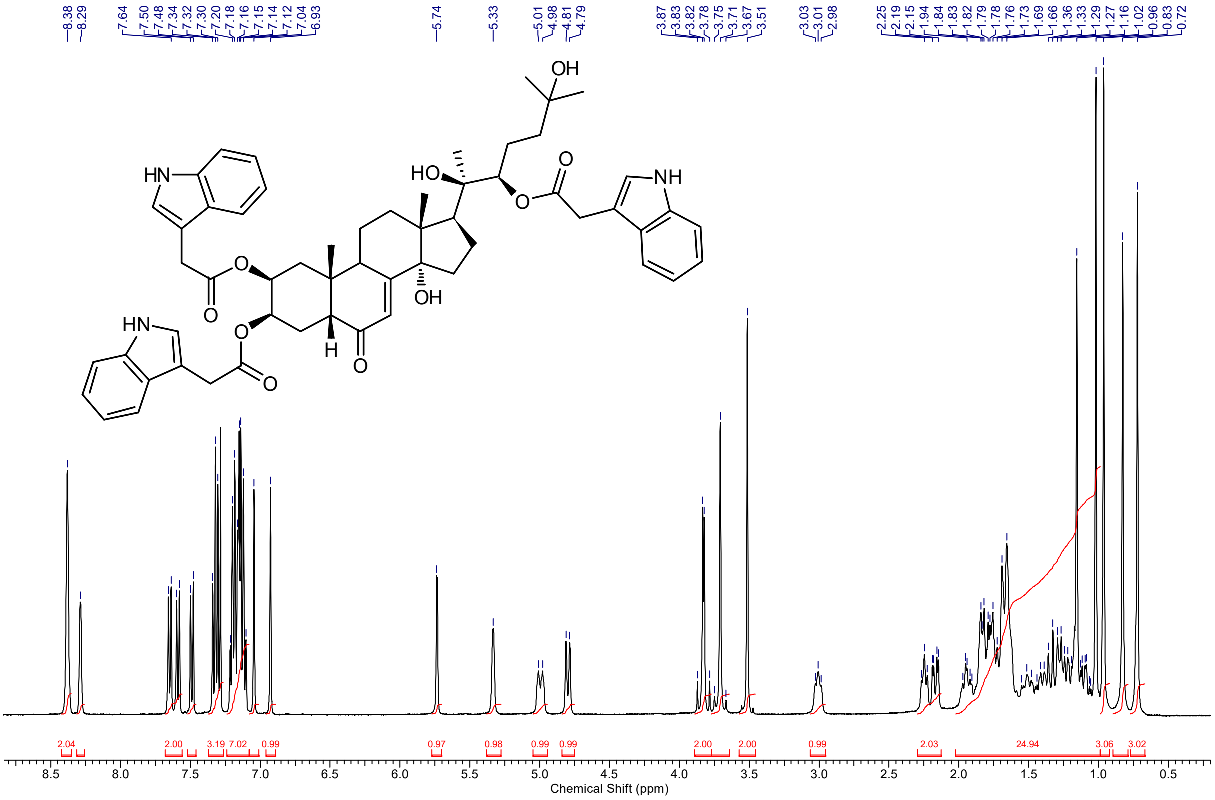

Compound 28: ^{13}C NMR (101 MHz, CDCl$_3$)

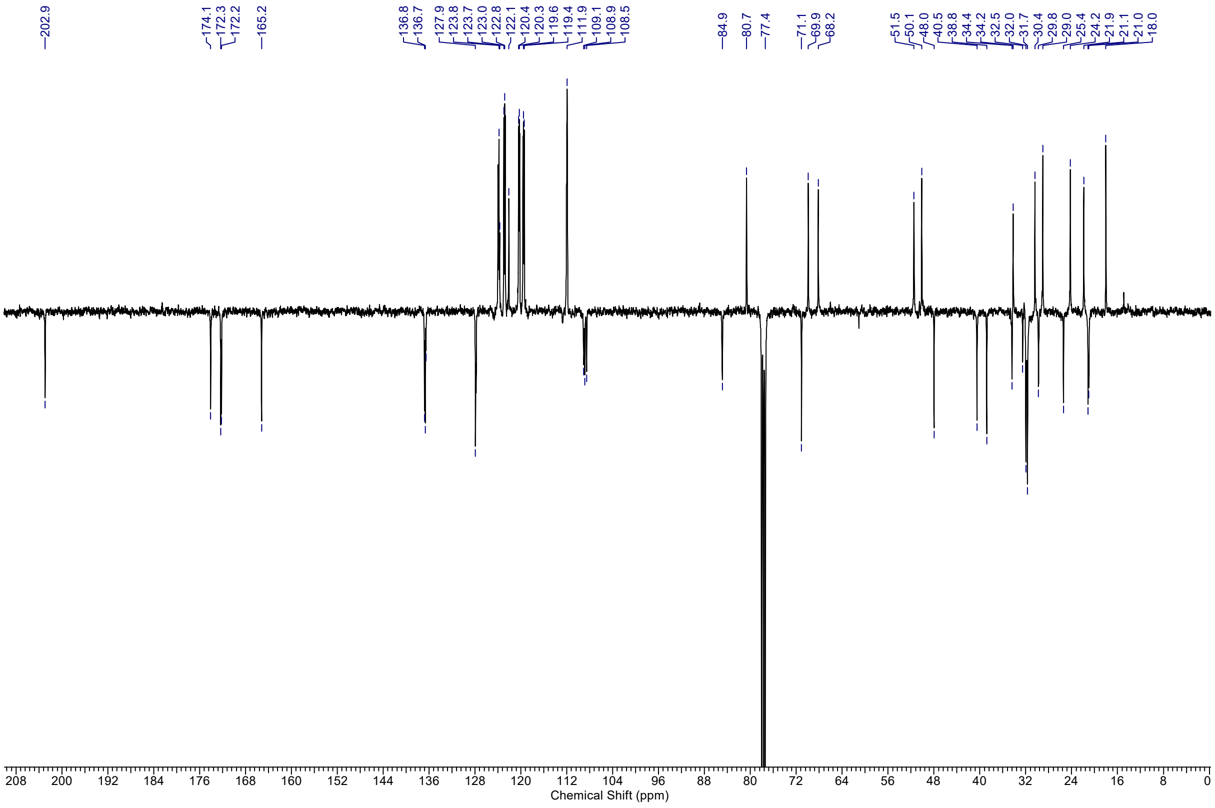

Supplement: Supplementary file 1 — np0c00334_si_001.pdf [file np0c00334_si_001.pdf]
